# Synergistic chiral iminium and palladium catalysis: Highly regio- and enantioselective [3 + 2] annulation reaction of 2-vinylcyclopropanes with enals

**DOI:** 10.3762/bjoc.12.127

**Published:** 2016-06-29

**Authors:** Haipan Zhu, Peile Du, Jianjun Li, Ziyang Liao, Guohua Liu, Hao Li, Wei Wang

**Affiliations:** 1 State Key Laboratory of Bioengineering Reactor, Shanghai Key Laboratory of New Drug Design and School of Pharmacy, East China University of Science and Technology, 130 Mei-long Road, Shanghai 200237, China; 2 Department of Chemistry, Shanghai Normal University, Shanghai 200234, China; 3 Department of Chemistry and Chemical Biology, University of New Mexico, Albuquerque, NM 87131-0001, USA

**Keywords:** [3 + 2] annulation, enals, synergistic catalysis, vinylcyclopropanes

## Abstract

A cooperative catalytic strategy of chiral iminium catalysis by regioselective activation of the C=C bond in enals and a transition metal promoting to open the 2-vinylcyclopropanes for highly regio- and enantioselective [3 + 2] cycloaddition reaction of 2-vinylcyclopropanes with α,β-unsaturated aldehydes has been developed.

## Introduction

**Scheme 1:**
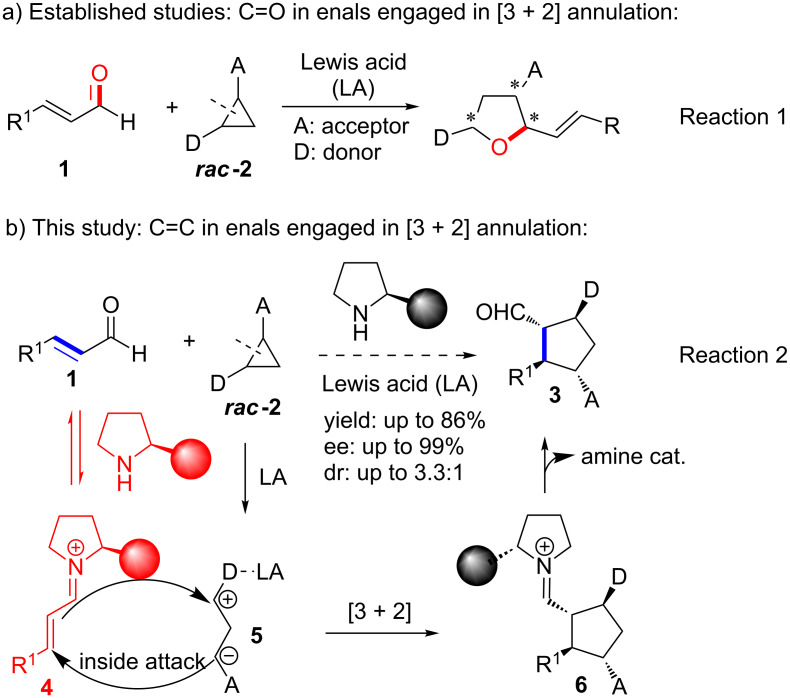
Catalytic regio- and enantioselective [3 + 2] annulation reactions of 2-vinylcyclopropanes with enals.

The power of “donor–acceptor” (D–A) cyclopropanes as versatile 1,3-dipolar components is fuelled by its capacity of serving a complementary approach to a wide array of 5-membered ring structures, which are difficult or impossible to access by classic [3 + 2] cycloaddition reactions [1–34]. In recent years, significant efforts have been devoted to developing a catalytic enantioselective version of the processes. In this context, the D–A cyclopropanes have been applied for the reaction with highly active dipolarophiles, such as electrophilic C=O [35], e.g., aldehydes [36–38], ketones [38–39], and imines [40], and nucleophilic enol ethers [38,41], enamides [42], and indoles [43]. Nonetheless, the reactions with the α,β-unsaturated aldehydes and ketones face important challenges. To the best of our knowledge, so far merely two catalyst manifolds have been realized to effect the transformations with C=C double bonds instead of C=O in the α,β-unsaturated systems. Tsuji described the first organometallic promoted non-asymmetric reaction between D–A cyclopropanes and methyl vinyl ketone and α,β-unsaturated esters [44]. Trost and co-workers orchestrated the only example of the enantioselective reaction of D–A cyclopropanes with C=C double bonds with Meldrum’s acid and alkylidenes or azlactone alkylidenes, catalyzed by the chiral Trost Pd(0)-complexes [45]. However, it is difficult to apply the catalytic system for the regio-controlled reaction with C=C bonds in α,β-unsaturated carbonyl compounds, particularly enals. The highly active aldehyde functionality reacts more favorably with the D–A cyclopropane resulting 1,3-dipoles, as elegantly demonstrated by Johnson and Waser for the formation of chiral tetrahydrofurans (Scheme 1, reaction 1) [36,38]. Achieving a regioselective control at the C=C bond rather than at C=O in enals represents a challenge and has not been reported.

Synergistic catalysis is a very important and useful strategy in organic synthesis by offering power for improving reaction efficiency and/or realizing impossible processes [46–55]. Recently, we developed an enantioselective addition of aldehydes to vinylpyridines and vinylarenes catalyzed by synergistic catalysis of iminium catalyst and Brønsted acid [56]. Herein we wish to disclose the first synergistic catalytic enantioselective [3 + 2] annulation reaction between 2-vinylcyclopropanes and enals via 1,4-addition (Scheme 1, reaction 2). The process proceeds highly regio- and enantioselectively with C=C bonds in enals. Notably, a synergistic catalytic system is implemented and makes this previously inaccessible [3 + 2] annulation transformation possible.

## Results and Discussion

To render the [3 + 2] annulation reaction to selectively act on the C=C double bond rather than on the aldehyde in enals **1**, we proposed a new cooperative iminium and Lewis acid (LA) catalysis strategy (Scheme 1, reaction 2) [49–50,57–76]. The iminium catalysis plays an important dual role in the process. The formed iminium ion **4** derived from aldehyde **1** and an amine catalyst activates the C=C bond and sterically blocks the attack of the C=N iminium ion functionality posed by the bulky amine catalyst. In parallel, a LA promotes to open the D–A cyclopropanes **2**. The cooperative activation of two independent substrates by respective iminium and Lewis acid catalysis may enable an unprecedented catalytic regio- and enantioselective [3 + 2] annulation process, which offers a new approach to synthetically important heavily functionalized chiral cyclopentane structures **3**, bearing at least 3 stereogenic centers in this one-pot operation [77–78].

To test the feasibility of the designed [3 + 2] annulation process [79–94], we started our investigation by carrying out the reaction between the commonly used D–A system dimethyl 2-vinylcyclopropane-1,1-dicarboxylate (**1a**) and *trans*-cinnamaldehyde (**2a**) catalyzed in the presence of a LA and chiral amine **I** in CH_2_Cl_2_ at rt for 48 h (Table 1). A series of Lewis acids were initially screened. FeCl_3_ and Cu(OTf)_2_ gave the 1,2-cycloaddition product tetrahydrofuran **4a** (Table 1, entries 1 and 2). It is also disappointing that others Lewis acids, such as CuCl_2_, MgI_2_, ZnBr_2_, ZnCl_2_ and FeCl_2_ failed to promote these processes (Table 1, entries 3–7). Inspired by Trost’s work of Pd(0)-catalyzed annulations of D–A cyclopropanes with C=C double bonds with Meldrum’s acid and alkylidenes or azlactone alkylidenes [45], we probed the Pd_2_(dba)_3_-dppe complex for the 1,4-addition cycloaddition reaction (Table 1, entry 8). It was found that the reaction took place to afford the desired cyclopentane **3a**.

**Table 1:** Screening of lewis acids.^a^

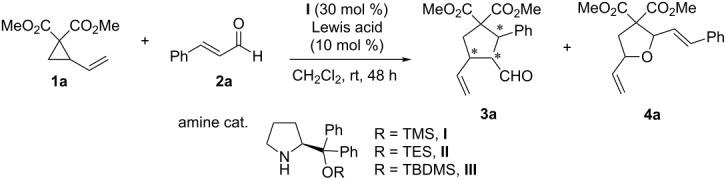			

Entry	LA	Yield (%)^b^, **3a**	Yield (%)^b^, **4a**

1	FeCl_3_	0	53
2	Cu(OTf)_2_	0	47
3	CuCl_2_	0	0
4	MgI_2_	0	0
5	ZnBr_2_	0	0
6	ZnCl_2_	0	0
7	FeCl_2_	0	0
8^c^	Pd_2_(dba)_3_	48	0

^a^The reaction was carried out with **1a** (36.8 mg, 0.2 mmol) and **2a** (26.4 mg, 0.2 mmol) in the presence of 10 mol % LA and 30 mol % amine **I** in 0.8 mL of CH_2_Cl_2_ at rt for 48 h. ^b^Isolated yields; ^c^5 mol % Pd_2_(dba)_3_ and 12.5 mol % dppe was used.

**Scheme 2:**
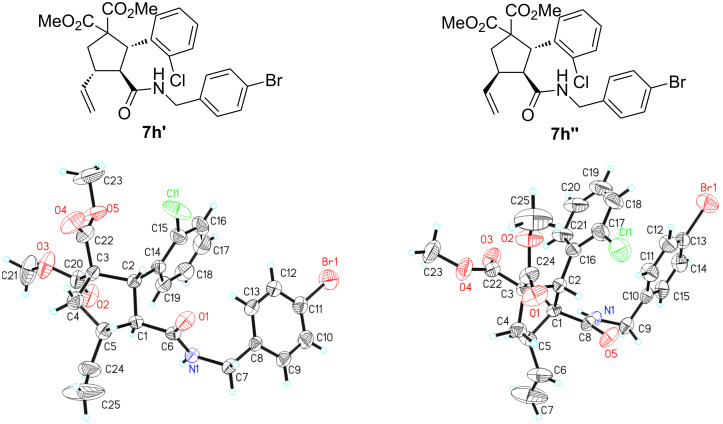
Single X-ray crystal structures of **7h’** and **7h’’**.

Encouraged by this result, we carried out further investigations of the co-catalysts promoted process (Table 2). First, we determined the diastereo- and enantioselectivity of the reaction. The ^1^H NMR of the reaction crude mixture showed three diastereoisomers. The two major diastereoisomers were determined to be (2*S*,3*S*,4*S*)-**3a’** and (2*S*,3*S*,4*R*)-**3a’’** in 2:1 ratio (Table 2, entry 1) based on single X-ray crystallographic analysis (see Scheme 2). Unfortunately, the third diastereoisomer **3a’’’** was too hard to be separated to determine its stereochemistry. The enantioselectivities of two major diastereoisomers are even more encouraging (80 and 76% ee). Further investigations of solvents revealed the medium-dependent effect (Table 2, entries 1–8). No reaction happened in toluene (Table 2, entry 2). Disappointing outcomes were also received in DCE, ether, CH_3_CN and EtOAc (Table 2, entries 3–6). Gratifyingly, in CHCl_3_ this reaction proceeded smoothly to furnish the desired cyclopentanes in 63% yield with 99% ee for major **3a’** and 83% ee for minor **3a’’** with a dr ratio of 1.7:1 (Table 2, entry 7). The reaction performed in THF was interesting: No reaction occurred at rt (Table 2, entry 8), but at 50 °C, 54% yield with high enantioselectivity for both isomers while **3a’’** as the major product (dr: **3a’’**:**3a’** = 5:1, Table 2, entry 9) was obtained. We decided to further optimize the reaction in CHCl_3_ accordingly (Table 2, entries 10–13). A longer reaction time helped to increase the reaction yield (60 h, 76% yield entry 10). More steric hindered amine catalysts with bigger TES and TBDMS groups, **II** and **III**, were then probed and gave rise to the slight drop of enantioseletivity (Table 2, entries 11 and 12). A further optimization of reaction conditions found that the addition of additional 0.5 equiv **1a** into the reaction mixture in 4 portions significantly improved the reaction yield (83%, Table 2, entry 13). In order to improve the diastereoselectivity of this reaction, other cyclopentanes used in Trost’s system were also tested in this reaction [45]. Unfortunately, the reactions proceeded slowly to afford the cycloaddition products in less than 10% yield.

**Table 2:** The optimization of reaction conditions.^a^

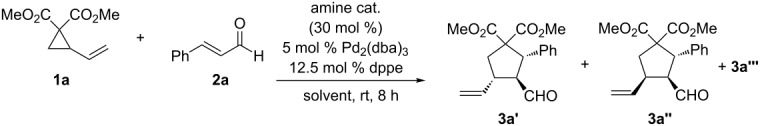					

Entry	Amine cat.	Solvent	Yield (%)^b^	ee (**3a’**, **3a’’**)^c^	dr (**3a’**:**3a’’**)^d^

1	I	CH_2_Cl_2_	48	80, 76	2:1
2	I	toluene	–	–	–
3	I	DCE	< 20	–	–
4	I	ether	< 20	–	–
5	I	CH_3_CN	< 20	–	–
6	I	EtOAc	< 20	–	–
7	I	CHCl_3_	63	99, 83	1.7:1
8	I	THF	–	–	–
9^e^	I	THF	54	90, 90	1:5
10^f^	I	CHCl_3_	76	99, 83	1.7:1
11^f^	II	CHCl_3_	76	96, 80	1.7:1
12^f^	III	CHCl_3_	61	97, 82	1.7:1
13^f,g^	I	CHCl_3_	83	99, 83	1.7:1

^a^The reaction was carried out with **1a** (36.8 mg, 0.2 mmol) and **2a** (26.4 mg, 0.2 mmol) in the presence of 5 mol % Pd_2_(dba)_3_, 12.5 mol % dppe and 30 mol % organic catalyst in 0.8 mL of solvent at rt for 48 h. ^b^Isolated yields. ^c^Determined by HPLC analysis. ^d^Determined by ^1^H NMR spectroscopy of the crude mixture. ^e^The reaction was run at 50 °C. ^f^The reaction was stirred for 60 h. ^g^Additional 0.5 equiv **1a** in 0.4 mL of CHCl_3_ was added into the reaction mixture in 4 portions every 12 h.

We then selected the use of co-catalysts of Pd_2_(dba)_3_ and organocatalyst **I** in CHCl_3_ at room temperature to evaluate the generality of this [3 + 2] annulation process by the variation of vinylcyclopropanes and enals (Table 3). The results exhibit that the synergistic catalyzed enantioselective [3 + 2] annulation process serves as a general approach to structurally chiral cyclopentanes bearing 3-consecutive stereogenic centers with high regio- and enantioselectivities. It was found that a wide range of aromatic α,β-unsaturated aldehydes can effectively participate in the process (Table 3, entries 1–12). The aromatic α,β-unsaturated aldehydes tethering electron-neutral, -withdrawing, and -donating substituents at the *para*-position of the phenyl ring gave good to high yields and excellent enantioselectivities for major isomer **3’** and minor **3’’’** products, while the electronic effect on enantioselectivity is more pronounced for minor **3’’** (Table 3, entries 1–5). A similar trend is observed with the aromatic α,β-unsaturated aldehydes with electron-withdrawing at *meta-*position (Table 3, entries 6 and 7). Those with electron-withdrawing, and -donating groups at *ortho-*position furnished excellent enantioselectivities for both **3’** and **3’’** products in cases studied (Table 3, entries 8–11). Moreover, the heteroaromatic furanyl α,β-unsaturated aldehyde **2l** can also be tolerated with good yield and 97% ee for the major product (Table 3, entry 12). More significantly, the more steric demanding D–A cyclopropane bearing a phenyl ring instead of H can effectively participate in the process to deliver the desired product with achieving an excellent level of enantioselectivity albeit a relatively low yield (Table 3, 46%, entry 13). It is noteworthy that although aliphatic enals also can engage in this [3 + 2] annulation reaction. Unfortunately, we could not separate them on chiral HPLC column for the determination of the enantioselectivity by all means we have attempted (data not shown).

**Table 3:** Scope of the [3 + 2] annulation reaction of D–A cyclopropanes with enals.^a^

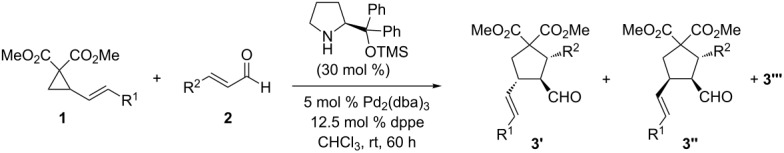				

Entry	R^1^, R^2^, **3**	Yield (%)^b^	ee (**3a’**, **3a’’**,**3a’’’**)^c^	dr (**3a’**:**3a’’**,**3a’’’**)^d^

1	H, Ph, **3a**	83	99, 83, 99	1.7:1:0.7
2	H, 4-ClC_6_H_4_, 3**b**	86	97, 77, 99	2:1:0.4
3	H, 4-BrC_6_H_4_, **3c**	84	94, 70, 99	2.5:1:0.5
4	H, 4-MeC_6_H_4_, **3d**	70	99, 85, 99	2:1:0.7
5	H, 4-MeOC_6_H_4_, **3e**	71	99, 99, 86	2:1:0.5
6	H, 3-FC_6_H_4_, 3**f**	85	97, 99, 76	2.5:1:0.6
7	H, 3-CF_3_C_6_H_4_, **3g**	61	90, 66, 99	3.3:1:0.5
8	H, 2-ClC_6_H_4_, 3**h**	65	98, 88, –	1.7:1:0.6
9	H, 2-BrC_6_H_4_ **3i**	77	99, 90, –	2.5:1:0.8
10	H, 2-MeC_6_H_4_, **3j**	72	99, 91, 99	3.3:1:0.4
11	H, 2-MeOC_6_H_4_, **3k**	81	99, 99, 92	2:1:0.8
12	H, 2-furanyl, **3l**	70	99, 97, 72	1.3:1:0.7
13^e^	Ph, 4-ClC_6_H_4_, **3m**	46	92, 86, 99	1.7:1:0.2

^a^Unless specified, see experimental section for details. ^b^Isolated yields. ^c^Determined by HPLC analysis. ^d^Determined by ^1^H NMR spectroscopy of crude product. ^e^The reaction was run at 50 °C for 120 h.

The absolute configuration of cyclopentanes **3’** and **3’’** were determined based on the derivatives **7h’** and **7h’’** of **3h** (Scheme 2) [95].

**Scheme 3:**
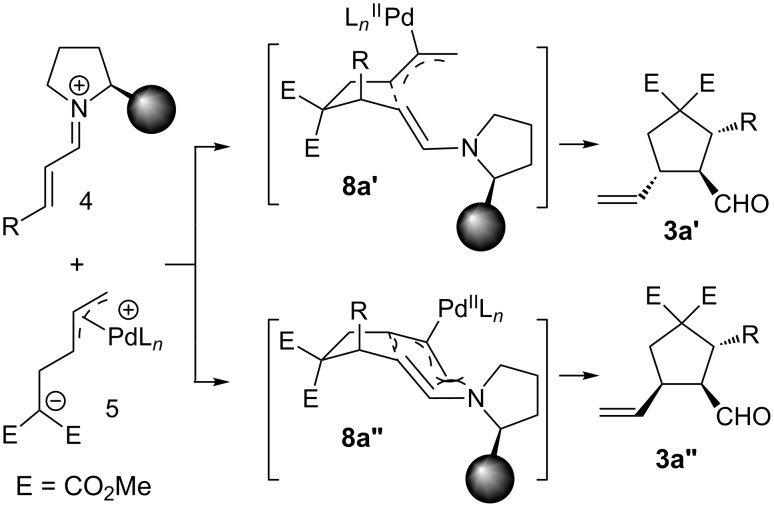
The proposed transition states.

We proposed two possible transition states (TS) **8a’** and **8a”** to rationalize the observed configurations (Scheme 3). The *trans*-C=C double bond in iminium ion **4** dictates the R group at pseudo axial position in the cyclic 5-membered ring TS **8a’** and **8a”**. This orientation avoids the A[1,3] strain induced by the catalyst-derived enamine. The Pd(II)-π3 complex moiety at pseudo axial and equatorial positions leads to respective TS **8a’** and **8a”**, while **8a’** is more stable due to the minimization of the A[1,3] interaction. Therefore, it is observed **3a’** produced from corresponding **8a’** as the major diastereomer whereas **3a”** as minor one.

## Conclusion

We have developed a cooperative catalytic strategy for highly regio- and enantioselective [3 + 2] cycloaddition reactions of vinylcyclopropanes with α,β-unsaturated aldehydes for the first time. The combination of a chiral iminium catalyst, which activates the C=C bond and blocks the C=O bond in enals, and a Lewis acid promoting to open the vinylcyclopropanes enables the annulation process to proceed with the challenging C=C bond. A high level of enantioselectivity could be achieved here. This previously unattainable [3 + 2] annulation transformation serves as a general approach to the preparation of new densely functionalized chiral cyclopentanes. This synergistic catalysis strategy holds great potentials for further exploration of new cycloaddition reactions involving enals and other D–A systems. The endeavor is being pursued in our laboratories.

## Experimental

### General procedure for the [3 + 2] annulation

A mixture of **1a** (0.2 mmol, 36.8 mg), **2a** (0.2 mmol, 26.4 mg), Pd_2_(dba)_3_ (0.01 mmol, 9.2 mg), dppe (0.025 mmol, 10 mg) and **I** (0.06 mmol, 18.5 mg) in 0.8 mL CHCl_3_ was stirred for 60 h at rt. During this period, **1a** (0.1 mmol, 18.4 mg) in 0.4 mL CHCl_3_ was added into the solution for total 4 times every 12 h, the mixture was purified by column chromatography on silica gel, eluted by petroleum ether/EtOAc = 20:1 to 10:1 to give the desired product **3a** in 83% yield as a colorless oil.

## Supporting Information

File 1 Experimental and analytical data.
