# Supplementary material for: Synergistic chiral iminium and palladium catalysis: Highly regio- and enantioselective [3 + 2] annulation reaction of 2-vinylcyclopropanes with enals
Source: Beilstein J Org Chem. 2016 Jun 29;12:1340–7. doi: 10.3762/bjoc.12.127 (PMC4979765; doi:10.3762/bjoc.12.127)
Supplement: File 1 — Experimental and analytical data. [file Beilstein_J_Org_Chem-12-1340-s001.pdf]

Supporting Information

for

Synergistic chiral iminium and palladium catalysis: Highly regio- and enantio-selective [3 + 2] annulation reaction of 2-vinylcyclopropanes with enals

Haipan Zhu[1,2], Peile Du[2], Jianjun Li[1], Ziyang Liao[1], Guohua Liu[2], Hao Li*[1], and Wei Wang*[1,3]

Address: [1]State Key Laboratory of Bioengineering Reactor, Shanghai Key Laboratory of New Drug Design and School of Pharmacy, East China University of Science and Technology, 130 Mei-long Road, Shanghai 200237, China, and [2]Department of Chemistry, Shanghai Normal University, Shanghai 200234, China, and [3]Department of Chemistry and Chemical Biology, University of New Mexico, Albuquerque, NM 87131-0001, USA

Email: Hao Li* - hli77@ecust.edu.cn; Wei Wang* - wwang@unm.edu

*Corresponding author

Experimental and analytical data

Table of Contents

1. **General** 2
2. **Synthesis of compounds 1 and characterization data** 2
3. **General procedure for synthesis of compounds 3 and characterization data** 2
4. **Determination of X-ray crystallographic structure 7h' and 7h'' and characterization data** 10
5. **References** 36
6. **Original ^{1}H and ^{13}C NMR spectra** 37
7. **Chiral HPLC analyses** 51

1. General

The reagents were purchased from commercial sources and used without further purification. ^{1}H NMR spectra were recorded at 400 MHz, ^{13}C NMR spectra were recorded at 100 MHz. ^{1}H NMR spectra were recorded with tetramethylsilane (δ = 0.00 ppm) as internal reference; ^{13}C NMR spectra was recorded with $CDCl_3$ (δ = 77.00 ppm) as internal reference. Chemical shifts were reported in parts per million (ppm, δ) downfield from tetramethylsilane. Proton coupling patterns are described as singlet (s), doublet (d), triplet (t), quartet (q), multiplet (m), and broad (br).

2. Synthesis of compounds 1 and characterization data

Dimethyl 2-vinylcyclopropane-1,1-dicarboxylate (**1a**) and dimethyl 2-styrylcyclopropane-1,1-dicarboxylate (**1b**) was prepared according to the procedure in literature.[1]

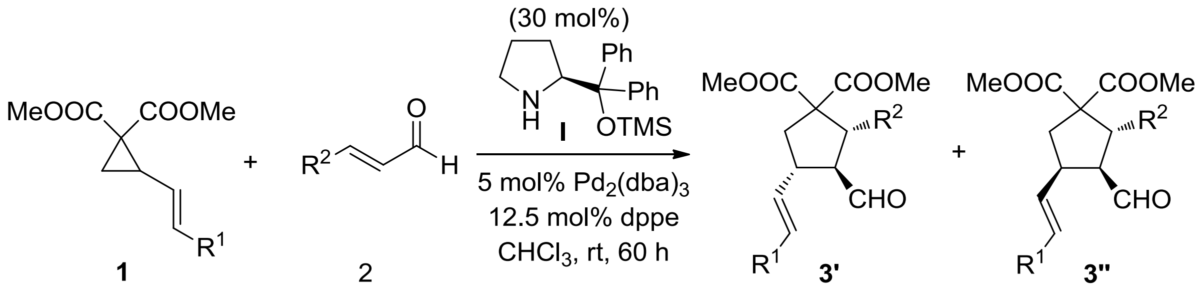

3. Gerneral procedure for the synthesis of compounds 3 (3a as example) and characterization data. A mixture of **1a** (0.2 mmol, 36.8 mg), **2a** (0.2 mmol, 26.4 mg), $Pd_2(dba)_3$ (0.01 mmol, 9.2 mg), dppe (0.025 mmol, 10 mg) and **I** (0.06 mmol, 18.5 mg) in 0.8 mL $CHCl_3$ was stirred 60 h at rt. During this period, **1a** (0.1 mmol, 18.4 mg) in 0.4 mL $CHCl_3$ was added into the solution for total 4 times every 12 h, the mixture was purified by column chromatography on silica gel, eluted by petroleum ether/EtOAc = 20:1 to 10:1 to give the desired product **3a** in 83% yield as a colorless oil.

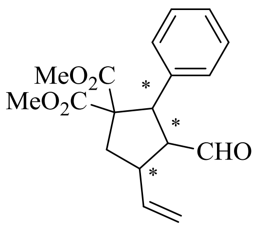

Dimethyl 3-formyl-2-phenyl-4-vinylcyclopentane-1,1-dicarboxylate (3a). 83% yield, colorless oil; ^{1}H NMR (400 MHz, $CDCl_3$): δ 9.63 (d, 1H, J =2.4 Hz), 9.60 (d, 0.6H, J =2.8 Hz), 9.37 (d, 0.4H, J = 0.8 Hz), 7.20-7.29 (m, 9.25 H), 7.08 (m,0.75 H), 5.68-5.96 (m, 2H), 5.02-5.20 (m, 4H), 4.68 (d, 1H, J =1 0 Hz), 4.57 (d, 0.4H, J = 8.8 Hz), 4.45 (d, 0.6H, J = 10 Hz), 3.77 (m, 6H), 3.50-3.62 (m, 2H), 3.31-3.36 (m,

0.4H), 3.24 (s, 1H), 3.17-3.21 (m, 0.4H), 3.14 (s, 4.8H), 3.07-3.10 (m, 0.6H), 2.95-3.00 (m, 1.4H), 2.83-2.90 (m, 0.6H), 2.72-2.78 (m, 0.6H), 2.40-2.45 (m, 0.6H), 2.07-2.14 (m, 1.4H); ^{13}C NMR (100 MHz, $CDCl_3$): δ 201.92, 201.06, 200.73, 172.39, 172.25, 172.20, 170.88, 169.96, 169.09, 140.13, 138.12, 137.79.137.60, 136.81, 136.50, 129.09, 128.70, 128.64, 128.59, 128.33, 128.21, 127.74, 127.63, 127.44, 117.48, 116.75, 115.12, 65.46, 64.92, 62.10, 61.53, 59.55, 53.16, 53.11, 52.89, 52.76, 52.26, 52.16, 51.23, 49.12, 44.68, 43.36, 41.46, 40.48, 39.88, 37.85; HRMS (ESI) m/z calcd for $C_{18}H_{20}O_5Na$ $(M+Na)^+$: 339.1208, found 339.1195 ; HPLC (Chiralpak OD-3, *i*PrOH/hexane = 1.0/99, flow rate = 1.0 mL/min, λ = 210 nm): t_{1major} = 29.10 min, t_{1minor} = 15.07 min, ee = 99%; t_{2major} = 22.23 min, t_{2minor} = 15.07 min, ee = 83%; t_{3major} = 17.689 min, t_{3minor} not observed, ee = 99%.

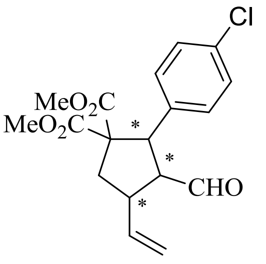

Dimethyl 2-(4-chlorophenyl)-3-formyl-4-vinylcyclopentane-1,1-dicarboxylate (3b). 86% yield, colorless oil; ^{1}H NMR (400 MHz, $CDCl_3$): δ 9.63 (d, 1H, *J* = 2.0 Hz), 9.59 (d, 0.5H, *J* = 2.8 Hz), 9.39 (s, 0.2H), 7.20-7.28 (m, 6.4H), 7.03-7.04 (m, 0.4H), 5.74-5.91 (m, 1.7H), 5.12-5.21 (m, 3.4H), 4.61(d, 1H, *J* = 10.8 Hz), 4.5 (d, 0.2H, *J* = 8.4 Hz), 4.42 (d, 0.5H, *J* = 10.4 Hz), 3.76-3.79 (m, 5.1H), 3.56-3.62 (m, 1.2H), 3.49-3.54 (m, 1.2H), 3.30 (s, 0.6H), 3.24 (s, 3H), 3.21 (s, 1.5H), 3.05-3.10 (m, 0.5H), 2.94-2.99 (m, 1.2H), 2.83-2.90 (m, 0.5H), 2.70-2.76 (m, 0.5H), 2.42-2.47 (m, 0.5H), 2.07-2.12 (m, 1.2H); ^{13}C NMR (100 MHz, $CDCl_3$): δ 201.35, 200.68, 200.26, 172.15, 172.07, 171.98, 170.78, 169.95, 168.93, 139.82, 137.41, 136.46, 136.41, 136.29, 135.38, 133.70, 133.51, 133.27, 130.49, 130.07, 129.99, 128.79, 128.45, 128.30, 117.60, 116.92, 115.33, 65.31, 64.68, 61.83, 61.40, 59.36, 53.20, 53.11, 52.89, 52.37, 52.28, 52.26, 52.11, 50.60, 48.52, 44.25, 43.54, 41.35, 40.48, 39.89, 37.81; HRMS (ESI) m/z calcd for $C_{18}H_{19}ClO_5Na$ $(M+Na)^+$: 373.0819, found 373.0798; HPLC (Chiralpak AS-H, *i*PrOH/hexane = 1.0/99, flow rate = 0.5 mL/min, λ = 210 nm): t_{1major} = 52.08 min, t_{1minor} = 34.72 min, ee = 97%; t_{2major} = 46.37 min, t_{2minor} = 39.25 min, ee = 77%; t_{3major} = 65.22min, t_{3minor} not observed, ee = 99%.

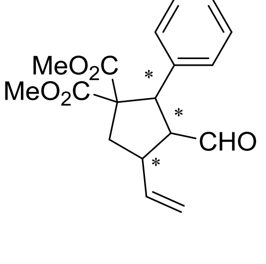

Dimethyl 2-(4-bromophenyl)-3-formyl-4-vinylcyclopentane-1,1-dicarboxylate (3c). 84% yield, colorless oil; ^{1}H NMR (400 MHz, $CDCl_3$): δ 9.54 (s, 1H), 9.50 (s, 0.4H), 9.30 (s, 0.2H), 7.31-7.34 (m, 3.3H), 7.06-7.11 (m, 2.7H), 6.88-6.90 (d, 0.4H, *J* = 8 Hz), 5.59-5.87 (m, 1.6H), 4.94-5.12 (m, 3.2H), 4.50 (d, 1H, *J* = 10.8 Hz), 4.46 (d, 0.2H, *J* = 8.4 Hz), 4.32 (d, 0.4H, *J* = 10 Hz), 3.68 (s, 1.8H), 3.70 (s, 3H), 3.48-3.53 (m, 1.2H), 3.41-3.46 (m, 1H), 3.26-3,28 (m, 0.2H), 3.22 (s, 0.6H), 3.16 (s, 3.0H), 3.13(s, 1.2H), 3.04-3.10 (m, 0.2H), 2.96-3.01 (m, 0.4H), 2.85-2.91 (m, 1H), 2.74-2.82 (m, 0.4H), 2.62-2.68 (m, 0.4H), 2.33-2.39 (m, 0.4H), 1.98-2.06 (m, 1.2H); ^{13}C NMR (100 MHz, $CDCl_3$): δ 201.40, 200.73, 200.29, 172.16, 172.08, 172.00, 170.80, 169.96, 168.91, 139.81, 137.38, 136.39, 136.25, 135.35, 133.69, 133.51, 133.27, 130.46, 130.05, 129.97, 128.81, 128.46, 128.32, 117.65, 116.99, 115.37, 65.28, 64.65, 61.82, 61.41, 59.36, 53.25, 53.17, 52.94, 52.43, 52.34, 52.32, 52.07, 50.57, 48.48, 44.27, 43.59, 41.34, 40.47, 39.88,37.77; HRMS (ESI) m/z calcd for $C_{18}H_{19}BrO_5Na$ $(M+Na)^+$: 417.0314, found: 417.0295; HPLC (Chiralpak AD-3, *i*PrOH/hexane =2.0/98, flow rate = 0.5 mL/min, λ = 210 nm): t_{1major} = 31.83 min, t_{1minor} = 42.60 min, ee = 94%; t_{2major} = 34.33 min, t_{2minor} = 41.18 min, ee = 70%; t_{3major} = 63.19 min, t_{3minor} = 49.55 min, ee = 99%.

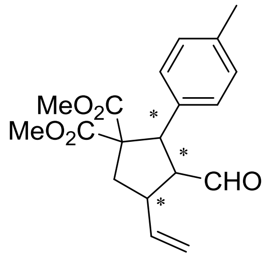

Dimethyl 3-formyl-2-(*p*-tolyl)-4-vinylcyclopentane-1,1-dicarboxylate (3d). in 70% yield, colorless oil; ^{1}H NMR (400 MHz, $CDCl_3$): δ 9.53 (d, 1H, *J* = 2.4 Hz), 9.52 (d, 0.5H, *J* = 2.8 Hz), 9.29 (s, 0.3H), 6.97-7.10 (m, 6.7H), 6.88-6.90 (m, 0.5H), 5.60-5.88 (m, 1.8H), 4.92-5.12 (m, 3.6H), 4.54 (d, 1H, *J* = 10 Hz), 4.45 (d, 0.3H, *J* = 8.4 Hz), 4.32 (d, 0.5H, *J* = 10 Hz), 3.68-3.69 (m, 5.4H), 3.48-3.53 (m, 1.3H), 3.41-3.46 (m, 1H), 3.22-3.27 (m, 0.3H), 3.20 (s, 0.9H), 3.12 (s, 3H), 3.10 (s, 1.5H), 2.98-3.08 (m, 0.8H), 2.86-2.91 (m, 1H), 2.75-2.82 (m, 0.5H), 2.63-2.69 (m, 0.5H), 2.31-2.37 (m. 0.5H), 2.20-2.21 (m, 5.4H), 1.98-2.08 (m, 1.3H); ^{13}C NMR (100 MHz, $CDCl_3$): δ 202.04, 201.14, 200.08, 172.45, 172.27, 172.20, 170.97, 170.03, 169.11, 140.30, 137.73, 137.37, 137.25, 137.03, 136.65, 134.98, 134.54, 133.59, 129.31, 129.00, 128.95, 128.88, 128.56, 128.45,117.36, 116,60, 114.95, 65.43, 64.88, 64.81, 62.14, 61.53, 59.49, 53.10, 53.04, 52.82, 52.42, 52.25, 52.16, 50.99, 48.95, 43.20, 41.28, 40.52, 39.78, 37.73, 21.02; HRMS (ESI) m/z calcd for $C_{19}H_{22}O_5Na$ $(M+Na)^+$: 353.1365, found 353.1342; HPLC (Chiralpak AD-3, *i*PrOH/hexane = 2.0/98, flow rate = 0.5 mL/min, λ = 210 nm): t_{1major} = 27.29 min, t_{1minor} not observed, ee = 99%; t_{2major} = 31.07 min, t_{2minor} = 35.47 min, ee = 85%; t_{3major} = 57.78 min, t_{3minor} not observed, ee = 99%.

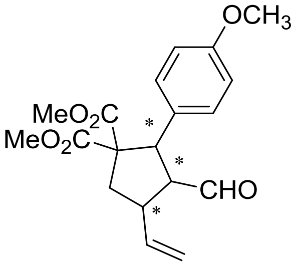

Dimethyl 3-formyl-2-(4-methoxyphenyl)-4-vinylcyclopentane-1,1-dicarboxylate (3e). 71% yield, colorless oil; ^{1}H NMR (400 MHz, $CDCl_3$): δ 9.62 (d, 1H, *J* = 1.6 Hz), 9.59 (d, 0.5H, *J* = 2.4 Hz), 9.39 (s, 0.3H), 7.18-7.28 (m, 2.9H), 7.01-7.03 (m, 0.6H), 6.78-6.82 (m, 3.7H), 5.69-5.90 (m, 1.8H), 5.10-5.21 (m, 3.6H), 4.62 (d, 1H, *J* = 10.4 Hz), 4.54 (d, 0.3H, *J* = 8.4 Hz), 4.40 (d, 0.5H, *J* = 10 Hz), 3.77-3.78 (m, 10.8H), 3.55-3.62 (m, 1.3H), 3.47-3.52 (m, 1H), 3.33-3.35 (m, 0.3H), 3.31 (s, 0,9H), 3.23 (s, 3H), 3.21 (s, 1.5H), 3.05-3.18 (m, 0.8H), 2.94-3.00 (m, 1H), 2.85-2.90 (m, 0.5H), 2.71-2.77 (m, 0.5H), 2.40-2.45 (m, 0.5H), 2.07-2.13 (m, 1.3H); ^{13}C NMR (100 MHz, $CDCl_3$): δ 202.09, 201.20, 200.81, 172.46, 172.32, 172.28, 171.02, 170.12, 169.14, 158.95, 158.89, 158.83, 140.26, 137.69, 136.61, 130.25, 129.78, 129.65, 129.51, 128.63, 117.39, 116.64, 114.98, 113.93, 113.66, 113.56, 65.47, 64.81, 64.73, 62.14, 61.57, 55.20, 55.15, 53.10, 53.04, 52.84, 52.31, 52.27, 52.01, 50.69, 48.61, 44.45, 43.26, 41.22, 40.45, 39.76, 37.69; HRMS (ESI) m/z calcd for $C_{19}H_{22}O_6Na$ $(M+Na)^+$: 369.1314, found: 369.1292; HPLC (Chiralpak IC-3, *i*PrOH/hexane = 2.0/98, flow rate = 1 mL/min, λ = 210 nm): t_{1major} = 100.50 min, t_{1minor} not observed, ee = 99%; t_{2major} = 63.13 min, t_{2minor} not observed, ee = 99%; t_{3major} = 80.07 min, t_{3minor} = 52.03 min, ee = 86%.

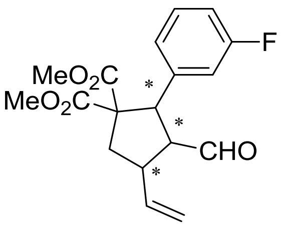

Dimethyl 2-(3-fluorophenyl)-3-formyl-4-vinylcyclopentane-1,1-dicarboxylate (3f). 85% yield, colorless oil; ^{1}H NMR (400 MHz, $CDCl_3$): δ 9.56 (s, 1H), 9.53 (s, 0.4H), 9.32 (s, 0.2H), 7.13-7.20 (m, 1.4H), 6.94-7.00 (m, 3H), 6.74-6.88 (m, 2H), 5.80-5.88 (m, 0.4H), 5.60-5.75 (m, 1.2H), 4.95-5.13 (m, 3.2H), 4.55 (d, 1H, *J* = 10.8 Hz), 4.48 (d, 0.2H, *J* = 8.8 Hz), 4.36 (d, 0.4H, *J* = 9.6 Hz), 3.68-3.71 (m, 4.8H), 3.49-3.54 (m, 1.2H), 3.42-3.47 (m, 1H), 3.26-2.28 (m, 0.2H), 3.23 (s, 0.6H), 3.15 (s, 3H), 3.13 (s, 1.5H), 2.98-3.05 (m, 0.6H), 2.87-2.92 (m, 1H), 2.75-2.82 (m, 0.4H), 2.63-2.70 (m, 0.4H), 2.34-2.39 (m, 0.4H), 1.98-2.07 (m, 1.2H); ^{13}C NMR (100 MHz, $CDCl_3$): δ 201.34, 200.68, 200.23, 172.12, 172.02, 171.93, 170.73, 169.87, 168.85, 163.81, 163.76, 161.32, 139.83, 137.37, 136.38, 130.23, 130.15, 129.81, 129.73, 129.62, 129.54, 124.67, 124.64, 124.24, 124.21, 124.14, 117.63, 116.97, 115.83, 115.74, 115.61, 115.34, 114.66, 114.45, 114.24, 65.29, 64.73, 64.71, 61.79, 61.39, 59.37, 53.24, 53.16, 52.94, 52.39, 52.29, 52.27, 50.79, 48.67, 48.66, 44.30, 43.53, 41.38,

40.47, 39.88, 37.79; HRMS (ESI) m/z calcd for $C_{18}H_{19}FO_5H$ $(M+H)^+$: 335.1295, found: 335.1270; HPLC (Chiralpak AS-H, *i*PrOH/hexane = 5.0/95, flow rate = 0.5 mL/min, λ = 210 nm): t_{1major} = 32.04 min, t_{1minor} = 24.48 min, ee = 97%; t_{2major} = 39.84 min, t_{2minor} not observed, ee = 99%; t_{3major} = 34.63 min, t_{3minor} = 23.04 min, ee = 76%.

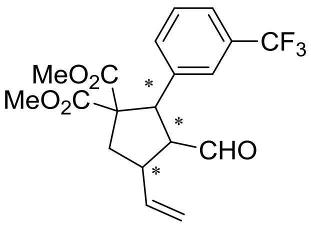

Dimethyl 3-formyl-2-(3-(trifluoromethyl)phenyl)-4-vinylcyclopentane-1,1-dicarboxylate (3g). 61% yield, colorless oil; ^{1}H NMR (400 MHz, $CDCl_3$): δ 9.57 (d, 1H, *J* = 2 Hz), 9.54 (d, 0.3H, *J* = 2.4 Hz), 9.35 (s, 0.2H), 7.194 (m, 6H), 5.82-5.91 (m, 0.3H), 5.65-5.74 (m, 1.2H), 4.96-5.15 (m, 3H), 4.59 (d, 1H, *J* = 10.4 Hz), 4.57 (d, 0.2H, *J* = 8.4 Hz), 4.32 (d, 0.3H, *J* = 10.4 Hz), 3.71 (s, 0.6H), 3.70 (s, 0.9H), 3.68 (s, 3H), 3.54-3.64 (m. 1.2H), 3.49-3.52 (m, 1H), 3.29-3.35 (m, 0.2H), 3.19 (s, 0.6H), 3.13 (s, 3H), 3.09 (s, 0.9H), 3.03-3.06 (m, 0.5H), 2.86-2.91 (m, 1H), 2.79-2.83 (m, 0.3H), 2.64-2.70 (m, 0.3H), 2,36-2.41 (m, 0.3H), 2.00-2.09 (m, 1.2H); ^{13}C NMR (100 MHz, $CDCl_3$): δ 201.00, 200.45, 199.95, 171.97, 171.92, 171.76, 170.67, 169.79, 168.72, 139.77, 139.11, 139.00, 138.01, 137.32, 136.40, 132.61, 132.18, 132.08, 130.64, 130.33, 129.18, 128.85, 128.66, 125.51, 125.40, 124.95, 124.91, 124.46, 124.42, 117.72, 117.06, 115.42, 65.41, 64.75, 61.63, 61.38, 59.26, 53.24, 53.16, 52.91, 54.42, 52.28, 52.18, 50.85, 48.80, 44.13, 43.66, 41.43, 40.57, 39.87; HRMS (ESI) m/z calcd for $C_{19}H_{19}F_3O_5Na$ $(M+Na)^+$: 407.1082, found: 407.1059; HPLC (Chiralpak OD-3, *i*PrOH/hexane = 0.5/99.5, flow rate = 0.5 mL/min, λ = 210 nm): t_{1major} = 47.39 min, t_{1minor} = 35.93 min, ee = 90%; t_{2major} = 50.70 min, t_{2minor} = 37.98 min, ee = 66%; t_{3major} = 44.67 min, t_{3minor} not observed, ee = 99%.

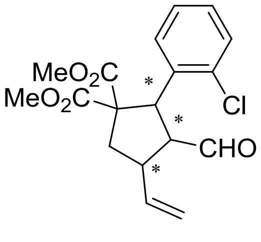

Dimethyl 2-(2-chlorophenyl)-3-formyl-4-vinylcyclopentane-1,1-dicarboxylate (3h). 65% yield, colorless oil; ^{1}H NMR (400 MHz, $CDCl_3$): δ 9.67(d, 1H, *J* = 2 Hz), 9.64 (d, 0.6H, *J* = 2.4 Hz), 9.36 (s, 0.4H), 7.34-7.38 (m, 2H), 7.05-7.23 (m, 6H), 5.81-5.93 (m, 1.6H), 5.68-5.76 (m, 0.4H), 5.30-5.32 (m, 0.4H), 5.09-5.24 (m, 5.3H), 5.01-5.04 (m, 0.3H), 3.79 (s, 1.2H), 3.78 (s, 3H), 3.73 (s, 1.8H), 3.54-3.64 (m, 1.4H), 3.40-3.52 (m, 1H), 3.27 (s, 1.8H), 3.26 (s, 1.2H), 3.20-3.25 (m, 0.4H), 3.17 (s, 3H), 2.86-2.97 (m, 2H), 2.75-2.81 (m, 0.6H), 2.41-2.47 (m, 1.2H), 2.10-2.23 (m, 1.4H); ^{13}C NMR (100 MHz, $CDCl_3$): δ 201.28, 200.29, 199.81, 172.21, 171.84, 171.44, 170.58, 169.33, 169.21, 139.70, 137.50, 137.22, 136.67, 136.20, 135.57, 135.06, 134.96, 134.75,

130.02, 129.85, 129.63, 129.20, 129.06, 128.84, 128.43, 127.08, 126.95, 126.55, 117.68, 116.87, 115.52, 64.98, 64.87, 64.68, 64.65, 61.91, 61.81, 53.32, 53.28, 52.99, 52.31, 52.26, 52.09, 47.62, 45.84, 45.36, 44.87, 42.84, 41.42, 41.12, 40.60, 38.47; HRMS (ESI) m/z calcd for $C_{18}H_{19}ClO_5Na$ $(M+Na)^+$: 373.0819, found: 373.0796; HPLC (Chiralpak OD-3, *i*PrOH/hexane = 1/99, flow rate = 1mL/min, λ = 210 nm): t_{1major} = 40.90 min, t_{1minor} = 24.42 min, ee = 98%; t_{2major} = 32.96 min, t_{2minor} = 20.82 min, ee = 88%.

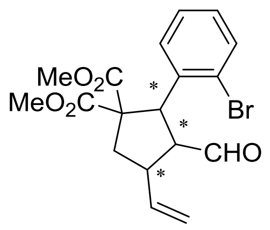

Dimethyl 2-(2-bromophenyl)-3-formyl-4-vinylcyclopentane-1,1-dicarboxylate (3i). 77% yield, colorless oil. ^{1}HNMR (400MHz, $CDCl_3$): δ 9.87 (d, 1H, *J* = 2.0 Hz), 9.83 (d, 0.4H, *J* = 2.4 Hz), 9.54 (s, 0.3H), 7.72-7.76 (m, 1.9H), 7.20-7.46 (m, 5.9H), 5.99-6.10 (m, 1.4H), 5.85-5.93 (m, 0.3H) , 5.19-5.50 (m, 5.1H), 3.97 (s, 1.2H), 3.96 (s, 3H), 3.92 (s, 0.9H), 3.70-3.85 (m, 1.4H), 3.58-3.63 (m, 1H), 3.48-3.49 (m, 0.3H), 3.45 (s, 1.2H), 3.44 (s, 0.9H), 3.34 (s, 3H), 3.06-3.15 (m, 1.7H), 2.92-2.98 (m, 0.4H), 2.59-2.63 (m, 0.8H), 2.28-2.43 (m, 1.3H); ^{13}C NMR (100 MHz, $CDCl_3$): δ 201.20, 200.16, 199.74, 172.18, 171.76, 171.44, 170.53, 169.23, 169.15, 139.70, 139.50, 138.61, 137.20, 136.65, 136.19, 133.45, 133.30, 133.00, 129.21, 129.11, 129.00, 128.93, 128.85, 128.69, 127.75, 127.62, 126.00, 125.86, 117.71, 116.86, 115.54, 65.30, 65.09, 65.05, 64.88, 62.69, 61.93, 53.35, 53.30, 53.03, 52.31, 52.27, 52.06, 50.77, 48.65, 48.04, 44.89, 42.74, 41.37, 41.20; HRMS (ESI) m/z calcd for $C_{18}H_{19}BrO_5Na$ $(M+Na)^+$: 417.0314, found: 417.0296; HPLC (Chiralpak OD-3, *i*PrOH/hexane = 1/99, flow rate = 1mL/min, λ = 210 nm): t_{1major} = 44.43 min, t_{1minor} not observed, ee =99%; t_{2major} = 33.04 min, t_{2minor} = 22.40min, ee = 90%.

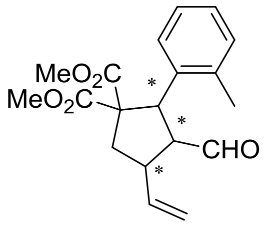

Dimethyl 3-formyl-2-(*o*-tolyl)-4-vinylcyclopentane-1,1-dicarboxylate (3j). 72% yield, colorless oil; ^{1}H NMR (400 MHz, $CDCl_3$): δ 9.67 (s, 1H), 9.66 (s, 0.3H), 9.26 (d, 0.1H, *J* = 1.2 Hz), 7.05-7.16 (m, 5.2H), 6.94-6.99 (m, 0.4H), 5.75-5.98 (m, 1.4H), 5.12-5.25 (m, 2.8H), 5.08 (d, 0.3H, *J* = 8.8 Hz), 5.04 (d, 0.1H, *J* = 10.4 Hz), 4.92 (d, 1H, *J* = 8.8 Hz), 3.79 (s, 0.1H), 3.78 (s, 3H), 3.74 (s, 0.9H), 3.60-3.66 (m, 0.4H), 3.41-3.46 (m, 0.3H), 3.26-3.30 (m, 0.1H), 3.18 (s, 0.3H), 3.12 (s, 0.9H), 3.08 (s, 3H), 2.99-3.05 (m, 1.4H), 2.78-2.87 (m, 2H), 2.60 (s, 0.9H), 2.46 (s, 3.3H), 2.41-2.43 (m, 1H), 2.06-2.16 (m, 1.1H); ^{13}C NMR (100 MHz, $CDCl_3$): δ 201.31, 200.74, 200.63,

172.67, 172.40, 170.68, 169.46, 169.36, 139.92, 138.34, 138.19, 138.07, 137.64, 137.48, 137.09, 136.37, 135.55, 130.73, 130.47, 130.24, 127.67, 127.44, 127.15, 126.94, 126.70, 126.26, 126.01, 125.59, 117.49, 116.81, 115.36, 65.88, 65.33, 65.14, 64.97, 63.55, 61.78, 53.19, 53.14, 52,89, 52.07, 52.03, 51.92, 47.02, 45.37, 44.98, 43.96, 43.39, 42.42, 41.23, 40.76, 38.83, 26.69, 20.55, 20.27; HRMS (ESI) m/z calcd for $C_{19}H_{22}O_5Na$ $(M+Na)^+$: 353.1365, found: 353.1343; HPLC (Chiralpak AD-3, *i*PrOH/hexane = 1/99, flow rate = 1mL/min, λ = 210 nm): t_{1major} = 17.52 min, t_{1minor} not observed, ee = 99%; t_{2major} = 22.89 min, t_{2minor} = 30.20 min, ee = 91%; t_{3major} = 25.19 min, t_{3minor} not observed, ee = 99%.

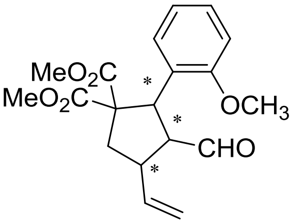

Dimethyl 3-formyl-2-(2-methoxyphenyl)-4-vinylcyclopentane-1,1-dicarboxylate (3k). 81% yield, colorless oil; ^{1}H NMR (400 MHz, $CDCl_3$): δ 9.56 (s, 1.5H), 9.20 (s, 0.5H), 6.97-7.21 (m, 3.9H), 6.71-6.82 (s, 3.9H), 5.75-5.84 (m, 1.5H), 5.63-5.71 (m, 0.5H), 4.92-5.13 (m, 4.5H), 4.85 (d, 1H, *J* = 8.4 Hz), 4.74 (d, 0.5H, *J* = 9.2 Hz), 3.64-3.71 (m, 11.7H), 3.48-3.58 (m, 2H), 3.38-3.45 (m, 0.5H), 3.12 (s, 4.5H), 3.11-3.16 (m, 1H), 3.08 (s, 1.5H), 3.01-3.03 (m, 0.5H), 2.77-2.86 (m, 2H), 2.23-2.32 (m, 0.5H), 1.95-2.02 (m, 1.5H); ^{13}C NMR (100 MHz, $CDCl_3$): δ 202.71, 201.55, 201.06, 172.61, 172.59, 171.99, 170.62, 169.74, 169.66, 157.88, 157.40, 156.72, 140.08, 137.92, 136.63, 130.69, 130.23, 129.58, 128.83, 128.69, 128.53, 127.65, 126.76, 125.41, 120.61, 120.54, 120.49, 117.11, 116.36, 115.15, 110.65, 110.40, 110.32, 64.85, 64.55, 63.94, 62.32, 60.70, 59.28, 55.26, 55.21, 53.10, 53.07, 52.78, 52.15, 51.93, 51.87, 45.84, 45.66, 45.42, 45.08, 43.19, 42.00, 40.88, 40.57, 39.01; HRMS (ESI) m/z calcd for $C_{19}H_{22}O_6Na$ $(M+Na)^+$: 369.1314, found: 369.1301; HPLC (Chiralpak AS-H, *i*PrOH/hexane = 4/96, flow rate = 1mL/min, λ = 210 nm): t_{1major} = 23.86 min, t_{1minor} not observed, ee = 99%; t_{2major} = 34.85 min, t_{2minor} not observed, ee = 99%; t_{3major} = 25.81 min, t_{3minor} = 22.39 min, ee = 92%.

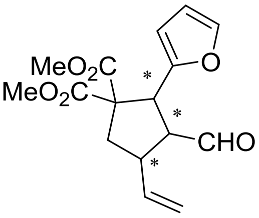

Dimethyl 3-formyl-2-(furan-2-yl)-4-vinylcyclopentane-1,1-dicarboxylate (3l). 70% yield, colorless oil; ^{1}H NMR (400 MHz, $CDCl_3$): δ 9.58 (s, 1.4H), 9.34 (s, 1H), 7.21-7.25 (m, 2.3H), 6.06-6.20 (m, 4.9H), 5.79-5.88 (m, 0.6H), 5.60-5.71 (m, 1.8H), 4.94-5.23 (m, 4.8H), 4.61 (d, 0.8H, *J* = 12 Hz), 4.50 (d, 1H, *J* = 8.4 Hz), 4.42 (d, 0.6H, *J* = 8.8Hz), 3.70 (s, 7.2H), 3.45-3.59 (m, 2.8H), 3.40 (s, 3.0H), 3.36 (s, 4.2H), 2.99-3.14 (m, 2.4H), 2.79-2.85 (m, 0.8H), 2.71-2.78 (m, 0.6H), 2.60-2.67 (m, 0.6H), 2.29-2.34 (m, 0.6H),

1.89-1.98 (m, 1.8H); ^{13}C NMR (100 MHz, $CDCl_3$): δ 201.25, 200.54, 200.40, 171.82, 171.63, 171.45, 170.62, 169.60, 169.07, 151,84, 151.53, 150.54, 142.47, 142.37, 141.83, 139.59, 137.94, 136.51, 117.50, 116.66, 115.50, 110.52, 110.45, 110.41, 109.29, 108.15, 108.10, 64.23, 63.76, 63.21, 60.31, 60.10, 57.59, 53.17, 52.94, 52.86, 52.73, 52.71, 46.07, 44.72, 43.90, 43.37, 42.90, 41.93, 40.07, 39.28, 38.08; HRMS (ESI) m/z calcd for $C_{16}H_{18}O_6Na$ $(M+Na)^+$: 329.1001, found: 329.0978; HPLC (Chiralpak AD-3, *i*PrOH/hexane = 1/99, flow rate = 0.5mL/min, λ = 210 nm): t_{1major} = 46.94 min, t_{1minor} = 42.68 min, ee = 99%, t_{2major} = 68.31 min, t_{2minor} = 40.46 min, ee = 97%; t_{3major} = 77.87 min, t_{3minor} = 37.72 min, ee = 72%.

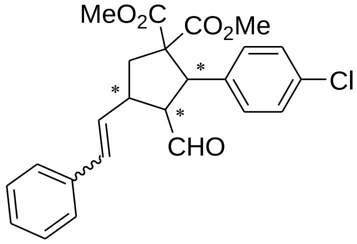

Dimethyl 2-(4-chlorophenyl)-3-formyl-4-((*E*)-styryl)cyclopentane-1,1-dicarboxylate (3m). 46% yield, colorless oil; ^{1}H NMR (400 MHz, $CDCl_3$): δ 9.66 (d, 0.6H, *J* = 1.6 Hz), 9.62 (d, 1H, *J* = 2.4 Hz),9.40 (s, 0.1H), 7.22-7.38 (m, 15.4H), 6.48-6.55 (m, 1.7H), 6.24-6.3 (m, 1H), 6.04-6.15 (m, 0.7H), 4.70 (d, 0.6H, *J* = 10.8 Hz), 4.59 (d, 0.1H, *J* = 8.8 Hz), 4.44 (d, 1H, *J* = 10.4 Hz), 3.80 (s, 0.3H), 3.79 (s, 3H), 3.76 (s, 1.8H), 3.66-3.69 (m, 0.1H), 3.57-3.62 (m, 0.6H), 3.40-3.45 (m, 0.1H), 3.30 (s, 0.3H), 3.25 (s, 1.8H), 3.22 (s, 3H), 3.15-3.20 (m, 1.6H), 3.00-3.09 (m, 1.7H), 2.79-2.85 (m, 1H), 2.43-2.58 (m, 1H), 2.12-2.20 (m, 0.7H); ^{13}C NMR (100 MHz, $CDCl_3$): δ 201.34, 200.74, 172.08, 172.01, 170.82, 170.06, 136.48, 136.38, 136.33, 136.29, 133.57, 133.33, 132.67, 132.04, 130.07, 130.01, 128.80, 128.64, 128.51, 128.37, 127.88, 64.75, 64.68, 62.20, 59.93, 53.19, 52.99, 52.38, 52.34, 50.66, 48.63, 43.79, 43.12, 41.25, 40.31; HRMS (ESI) m/z calcd for $C_{24}H_{23}ClO_5Na$ $(M+Na)^+$: 449.1132, found: 449.1112; HPLC (Chiralpak AD-3, *i*PrOH/hexane = 2/98, flow rate = 1mL/min, λ = 210 nm): t_{1major} = 44.40 min, t_{1minor} = 68.41 min, ee = 92%; t_{2major} = 41.75 min, t_{2minor} = 85.78 min, ee = 86%; t_{3major} = 74.32 min, t_{3minor} not observed, ee = 99%.

4. Determination of X-ray crystallographic structure 7h’.

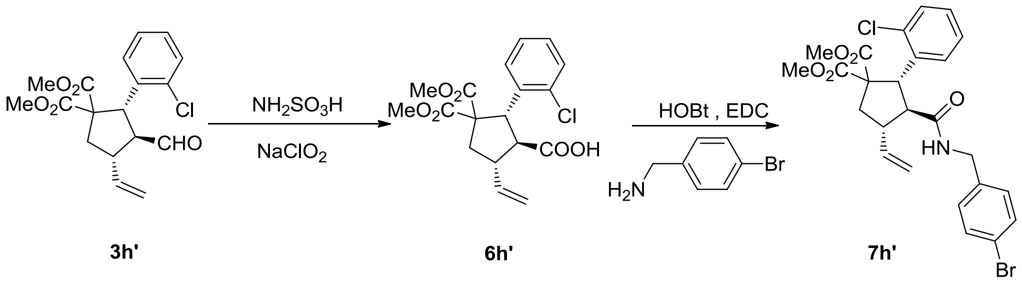

(2*R*,3*S*,4*S*)-Dimethyl 3-((4-bromobenzyl)carbamoyl)-2-(2-chlorophenyl)-4-vinylcyclopentane-1,1-dicarboxylate (7h’). A solution of **3h’** (0.16 mmol, 56 mg) in toluene (1 mL) was added into the mixture of NH_2SO_3H (0.38 mmol, 37 mg) in water (0.3 mL). After the mixture was cooled to 0 °C, a solution of sodium chlorite (0.32 mmol, 29 mg) in water (0.22 mL) was added dropwise, and the mixture was stirred for 20 min. Aqueous solution of sodium sulfite (21 mg in 0.1 mL water) and aqueous NaOH (27 mg in 6 mL water) were successively added to the reaction mixture. The aqueous layer was separated and acidified with 37% HCl. The resulting precipitate was collected by filtration to give **6h’** without purification to use in next step after drying.[2] A solution of **6h’** (0.092 mmol, 34 mg), EDC·HCl (0.27 mmol, 52 mg), HOBt (0.27 mmol, 37 mg) and TEA (0.027 mmol, 27 mg) in 1.5 mL DCM was stirred for 15 min. (4-Bromophenyl)methanamine (0.24 mmol, 45 mg) was added into the solution and stirred overnight at rt. After work-up with H_2O and separation, the organic solution was dried, concentrated, and the residue was separated by column chromatography on silica gel, eluted by petroleum ether/EtOAc = 10:1 to give the title compound **7h’** in 50% yield. ^{1}H NMR (400 MHz, $CDCl_3$): δ 7.14-7.36 (m, 6.5H), 6.92-6.94 (m, 1.5H), 5.80-5.87 (m, 2H), 5.00-5.10 (m, 3H), 4.19-4.33 (m, 2H), 3.7 (s, 3H), 3.31-3.38 (m, 2H), 3.30 (s, 3H), 2.81-2.85 (m, 1H), 2.32-2.36 (m, 1H); ^{13}C NMR (100 MHz, $CDCl_3$): δ 117.32, 170.86, 170.61, 137.25, 137.02, 135.50, 131.45, 129.88, 129.28, 128.93, 128.34, 126.50, 121.07, 116.83, 61.58, 56.98, 52.81, 52.15, 48.18, 45.24, 42.73, 40.88. HRMS (EI) m/z calcd for $C_{25}H_{25}BrClNO_5$ (M): 533.0605, found 533.0609; HPLC (Chiralpak AD-3, *i*PrOH/hexane = 10/90, flow rate = 1mL/min, λ = 210 nm): t_{major} = 39.34 min, t_{minor} not observed, ee = 99%.

X-ray structure of compound 7h’

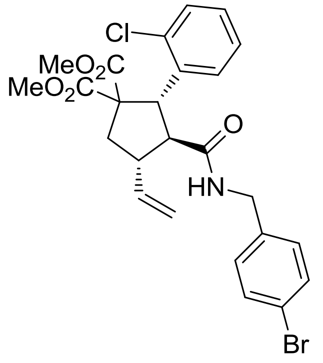

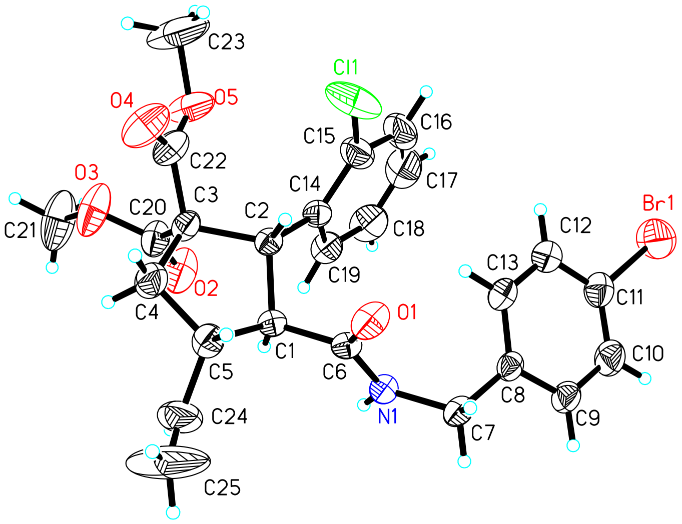

Table 1. Crystal data and structure refinement for 175.

Identification code	175
Empirical formula	C25 H25 Br Cl N O5
Formula weight	534.82
Temperature	296(2) K
Wavelength	1.54178 A
Crystal system, space group	Triclinic, P1
Unit cell dimensions	a = 9.6339(19) A alpha = 103.90(3) deg.
	b = 11.781(2) A beta = 107.58(3) deg.
	c = 13.001(3) A gamma = 102.61(3) deg.
Volume	1296.2(4) A^3
Z, Calculated density	2, 1.370 Mg/m^3
Absorption coefficient	3.394 mm^-1
F(000)	548
Crystal size	0.34 x 0.16 x 0.14 mm
Theta range for data collection	3.78 to 67.04 deg.
Limiting indices	-11<=h<=11, -13<=k<=13, -15<=l<=15
Reflections collected / unique	14247 / 7779 [R(int) = 0.0229]
Completeness to theta = 67.04	92.1 %
Absorption correction	Semi-empirical from equivalents
Max. and min. transmission	0.7529 and 0.5536
Refinement method	Full-matrix least-squares on F^2
Data / restraints / parameters	7779 / 35 / 614
Goodness-of-fit on F^2	1.038
Final R indices [I>2sigma(I)]	R1 = 0.0545, wR2 = 0.1556
R indices (all data)	R1 = 0.0551, wR2 = 0.1566
Absolute structure parameter	0.074(17)
Largest diff. peak and hole	0.515 and -0.823 e.A^-3

Table 2. Atomic coordinates (x 10^4) and equivalent isotropic displacement parameters (A^2 x 10^3) for 175.

	x	y	z	U(eq)
Br(1)	10031(1)	9857(1)	850(1)	119(1)
Br(2)	6204(2)	986(2)	-10480(3)	131(1)
Br(2A)	5496(13)	876(4)	-10058(4)	210(3)
Cl(1)	3945(3)	8825(2)	-4747(2)	134(1)
Cl(2)	7120(1)	2188(1)	-4899(1)	94(1)
O(1)	3952(3)	5280(3)	-4725(3)	67(1)
O(2)	7072(4)	7026(5)	-7275(3)	103(1)
O(3)	5267(5)	7133(6)	-8734(4)	113(2)
O(4)	2157(4)	7198(5)	-8008(4)	103(1)
O(5)	4413(4)	8685(4)	-7178(4)	101(1)
O(6)	8994(3)	5468(3)	-4802(2)	61(1)
O(7)	8924(5)	3229(4)	-2103(3)	85(1)
O(8)	11106(6)	3401(5)	-748(3)	122(2)
O(9)	13920(4)	4076(5)	-1878(5)	109(1)
O(10)	12061(5)	2301(4)	-2454(4)	106(1)
N(1)	6325(3)	5146(3)	-4266(2)	46(1)
N(2)	10964(3)	5593(3)	-5386(2)	49(1)
C(1)	5210(3)	5553(3)	-6019(3)	44(1)
C(2)	4960(3)	6792(3)	-6029(3)	45(1)
C(3)	4488(4)	6694(4)	-7325(3)	55(1)
C(4)	3550(7)	5328(5)	-7939(4)	84(1)
C(5)	3902(4)	4620(4)	-7104(3)	60(1)
C(6)	5123(3)	5307(3)	-4940(3)	43(1)
C(7)	6444(4)	4994(4)	-3163(3)	53(1)
C(8)	7316(4)	6180(4)	-2182(3)	52(1)
C(9)	8386(5)	6203(5)	-1174(4)	68(1)
C(10)	9207(6)	7292(6)	-269(4)	81(1)
C(11)	8941(5)	8345(5)	-385(4)	72(1)
C(12)	7908(7)	8371(5)	-1355(4)	81(1)
C(13)	7101(6)	7286(5)	-2239(4)	75(1)
C(14)	6205(4)	7913(3)	-5121(3)	54(1)
C(15)	5846(7)	8871(5)	-4483(4)	78(1)
C(16)	7001(11)	9889(5)	-3618(5)	105(2)
C(17)	8497(11)	9962(6)	-3379(6)	110(2)
C(18)	8845(7)	9039(6)	-3963(5)	93(2)
C(19)	7726(5)	8013(4)	-4820(4)	65(1)
C(20)	5799(5)	6988(5)	-7734(4)	71(1)
C(21)	6346(10)	7351(12)	-9299(6)	145(4)
C(22)	3521(5)	7527(5)	-7567(4)	71(1)

C(23)	3685(13)	9589(9)	−7373(12)	176(5)
C(24)	4218(11)	3478(7)	−7547(7)	135(3)
C(25)	3550(30)	2520(13)	−7790(30)	300(20)
C(25A)	4590(50)	2740(30)	−7520(40)	201(14)
C(26)	11251(3)	5203(3)	−3604(3)	47(1)
C(27)	10436(4)	3986(3)	−3505(3)	46(1)
C(28)	11351(4)	4097(4)	−2234(3)	58(1)
C(29)	11991(6)	5518(4)	−1593(3)	70(1)
C(30)	11464(4)	6145(4)	−2462(3)	56(1)
C(31)	10305(3)	5436(3)	−4641(3)	43(1)
C(32)	10162(4)	5751(4)	−6444(3)	56(1)
C(33)	9144(5)	4572(4)	−7392(4)	62(1)
C(34)	8173(11)	4616(7)	−8391(6)	119(3)
C(35)	7250(14)	3545(9)	−9278(8)	164(5)
C(36)	7301(10)	2431(8)	−9127(7)	125(3)
C(37)	8217(9)	2386(6)	−8182(5)	98(2)
C(38)	9122(7)	3423(5)	−7302(4)	78(1)
C(39)	10210(5)	2840(4)	−4439(3)	55(1)
C(40)	8804(6)	1958(4)	−5067(4)	69(1)
C(41)	8596(9)	865(5)	−5869(4)	92(2)
C(42)	9799(12)	668(7)	−6099(6)	112(2)
C(43)	11212(12)	1544(8)	−5565(7)	110(2)
C(44)	11427(7)	2640(5)	−4734(5)	80(1)
C(45)	10273(6)	3504(4)	−1722(3)	66(1)
C(46)	10244(14)	2837(11)	−166(6)	191(7)
C(47)	12611(5)	3521(5)	−2152(4)	76(1)
C(48)	13141(13)	1617(11)	−2540(14)	190(6)
C(49)	12505(7)	7405(5)	−2189(5)	87(2)
C(50)	12211(17)	8417(10)	−1895(13)	194(7)
H(1A)	7082	5128	−4482	55
H(2A)	11900	5600	−5228	59
H(1B)	6206	5550	−6066	52
H(2B)	4034	6776	−5859	54
H(4A)	2464	5237	−8216	100
H(4B)	3812	5002	−8589	100
H(5A)	2999	4397	−6906	72
H(7A)	6955	4384	−3052	63
H(7B)	5421	4689	−3168	63
H(9A)	8555	5472	−1104	82
H(10A)	9922	7299	400	97
H(12A)	7753	9108	−1416	97
H(13A)	6384	7296	−2899	90
H(16A)	6745	10517	−3205	126
H(17A)	9271	10645	−2815	132

H(18A)	9870	9087	−3788	112
H(19A)	8015	7384	−5197	78
H(21A)	5832	7432	-10023	218
H(21B)	6742	6670	−9418	218
H(21C)	7177	8094	−8829	218
H(23A)	4439	10391	−7041	264
H(23B)	2931	9568	−7029	264
H(23C)	3195	9416	−8180	264
H(25A)	4047	1931	−7714	358
H(25B)	2487	2287	−8061	358
H(25C)	3891	1960	−7749	241
H(25D)	5639	2845	−7256	241
H(26A)	12254	5197	−3635	56
H(27A)	9410	4011	−3549	55
H(29A)	11605	5727	−988	83
H(29B)	13103	5782	−1255	83
H(30A)	10454	6210	−2499	68
H(32A)	10910	6178	−6695	67
H(32B)	9540	6272	−6305	67
H(34A)	8136	5374	−8470	143
H(35A)	6609	3573	−9962	197
H(37A)	8250	1624	−8111	118
H(38A)	9741	3363	−6626	94
H(41A)	7633	275	−6245	110
H(42A)	9677	−73	−6627	135
H(43A)	12032	1411	−5756	132
H(44A)	12388	3235	−4377	96
H(46A)	10940	2813	529	286
H(46B)	9619	2014	−649	286
H(46C)	9601	3310	9	286
H(48A)	12627	753	−2745	285
H(48B)	13979	1889	−1817	285
H(48C)	13526	1759	−3113	285
H(49A)	13461	7462	−2236	105
H(50A)	11270	8406	−1837	233
H(50C)	12938	9163	−1741	233

Table 3. Bond lengths [A] and angles [deg] for 175.

Br(1)-C(11)	1.909(5)	C(7)-H(7A)	0.9700
Br(2)-C(36)	1.939(8)	C(7)-H(7B)	0.9700
Br(2A)-C(36)	2.029(10)	C(8)-C(13)	1.378(6)
Cl(1)-C(15)	1.744(6)	C(8)-C(9)	1.392(6)
Cl(2)-C(40)	1.769(5)	C(9)-C(10)	1.387(8)
O(1)-C(6)	1.237(4)	C(9)-H(9A)	0.9300
O(2)-C(20)	1.177(6)	C(10)-C(11)	1.353(8)
O(3)-C(20)	1.313(6)	C(10)-H(10A)	0.9300
O(3)-C(21)	1.457(8)	C(11)-C(12)	1.362(7)
O(4)-C(22)	1.192(6)	C(12)-C(13)	1.371(8)
O(5)-C(22)	1.323(7)	C(12)-H(12A)	0.9300
O(5)-C(23)	1.428(8)	C(13)-H(13A)	0.9300
O(6)-C(31)	1.226(4)	C(14)-C(19)	1.368(6)
O(7)-C(45)	1.179(6)	C(14)-C(15)	1.406(6)
O(8)-C(45)	1.328(6)	C(15)-C(16)	1.398(9)
O(8)-C(46)	1.443(8)	C(16)-C(17)	1.358(12)
O(9)-C(47)	1.184(6)	C(16)-H(16A)	0.9300
O(10)-C(47)	1.331(7)	C(17)-C(18)	1.336(12)
O(10)-C(48)	1.462(11)	C(17)-H(17A)	0.9300
N(1)-C(6)	1.307(4)	C(18)-C(19)	1.387(7)
N(1)-C(7)	1.460(4)	C(18)-H(18A)	0.9300
N(1)-H(1A)	0.8600	C(19)-H(19A)	0.9300
N(2)-C(31)	1.335(4)	C(21)-H(21A)	0.9600
N(2)-C(32)	1.437(5)	C(21)-H(21B)	0.9600
N(2)-H(2A)	0.8600	C(21)-H(21C)	0.9600
C(1)-C(6)	1.521(4)	C(23)-H(23A)	0.9600
C(1)-C(2)	1.532(5)	C(23)-H(23B)	0.9600
C(1)-C(5)	1.537(5)	C(23)-H(23C)	0.9600
C(1)-H(1B)	0.9800	C(24)-C(25A)	1.016(18)
C(2)-C(14)	1.510(5)	C(24)-C(25)	1.085(18)
C(2)-C(3)	1.574(5)	C(25)-H(25A)	0.9300
C(2)-H(2B)	0.9800	C(25)-H(25B)	0.9300
C(3)-C(20)	1.517(6)	C(25A)-H(25C)	0.9300
C(3)-C(22)	1.517(6)	C(25A)-H(25D)	0.9300
C(3)-C(4)	1.535(7)	C(26)-C(31)	1.506(5)
C(4)-C(5)	1.525(6)	C(26)-C(27)	1.532(5)
C(4)-H(4A)	0.9700	C(26)-C(30)	1.551(5)
C(4)-H(4B)	0.9700	C(26)-H(26A)	0.9800
C(5)-C(24)	1.465(8)	C(27)-C(39)	1.507(5)
C(5)-H(5A)	0.9800	C(27)-C(28)	1.578(5)
C(7)-C(8)	1.507(6)	C(27)-H(27A)	0.9800

C(28)-C(47)	1.503(7)	C(39)-C(40)	1.375(6)
C(28)-C(45)	1.528(6)	C(39)-C(44)	1.388(6)
C(28)-C(29)	1.564(6)	C(40)-C(41)	1.380(7)
C(29)-C(30)	1.516(6)	C(41)-C(42)	1.334(11)
C(29)-H(29A)	0.9700	C(41)-H(41A)	0.9300
C(29)-H(29B)	0.9700	C(42)-C(43)	1.368(13)
C(30)-C(49)	1.488(7)	C(42)-H(42A)	0.9300
C(30)-H(30A)	0.9800	C(43)-C(44)	1.397(9)
C(32)-C(33)	1.504(6)	C(43)-H(43A)	0.9300
C(32)-H(32A)	0.9700	C(44)-H(44A)	0.9300
C(32)-H(32B)	0.9700	C(46)-H(46A)	0.9600
C(33)-C(34)	1.371(8)	C(46)-H(46B)	0.9600
C(33)-C(38)	1.384(7)	C(46)-H(46C)	0.9600
C(34)-C(35)	1.379(12)	C(48)-H(48A)	0.9600
C(34)-H(34A)	0.9300	C(48)-H(48B)	0.9600
C(35)-C(36)	1.381(13)	C(48)-H(48C)	0.9600
C(35)-H(35A)	0.9300	C(49)-C(50)	1.282(15)
C(36)-C(37)	1.300(10)	C(49)-H(49A)	0.9300
C(37)-C(38)	1.350(9)	C(50)-H(50A)	0.9300
C(37)-H(37A)	0.9300	C(50)-H(50C)	0.9300
C(38)-H(38A)	0.9300		

C(20)-O(3)-C(21)	116.8(5)	C(3)-C(2)-H(2B)	105.6
C(22)-O(5)-C(23)	117.0(6)	C(20)-C(3)-C(22)	109.0(3)
C(45)-O(8)-C(46)	115.5(6)	C(20)-C(3)-C(4)	108.6(4)
C(47)-O(10)-C(48)	116.7(6)	C(22)-C(3)-C(4)	111.8(4)
C(6)-N(1)-C(7)	123.2(2)	C(20)-C(3)-C(2)	115.9(3)
C(6)-N(1)-H(1A)	118.4	C(22)-C(3)-C(2)	109.2(3)
C(7)-N(1)-H(1A)	118.4	C(4)-C(3)-C(2)	102.3(3)
C(31)-N(2)-C(32)	122.2(2)	C(5)-C(4)-C(3)	109.0(4)
C(31)-N(2)-H(2A)	118.9	C(5)-C(4)-H(4A)	109.9
C(32)-N(2)-H(2A)	118.9	C(3)-C(4)-H(4A)	109.9
C(6)-C(1)-C(2)	110.1(3)	C(5)-C(4)-H(4B)	109.9
C(6)-C(1)-C(5)	112.2(3)	C(3)-C(4)-H(4B)	109.9
C(2)-C(1)-C(5)	103.6(3)	H(4A)-C(4)-H(4B)	108.3
C(6)-C(1)-H(1B)	110.2	C(24)-C(5)-C(4)	115.1(5)
C(2)-C(1)-H(1B)	110.2	C(24)-C(5)-C(1)	113.9(4)
C(5)-C(1)-H(1B)	110.2	C(4)-C(5)-C(1)	105.5(3)
C(14)-C(2)-C(1)	115.4(3)	C(24)-C(5)-H(5A)	107.3
C(14)-C(2)-C(3)	119.9(3)	C(4)-C(5)-H(5A)	107.3
C(1)-C(2)-C(3)	103.6(3)	C(1)-C(5)-H(5A)	107.3
C(14)-C(2)-H(2B)	105.6	O(1)-C(6)-N(1)	122.7(3)
C(1)-C(2)-H(2B)	105.6	O(1)-C(6)-C(1)	120.0(3)

N(1)-C(6)-C(1)	117.3(2)	O(2)-C(20)-C(3)	126.7(4)
N(1)-C(7)-C(8)	112.4(3)	O(3)-C(20)-C(3)	108.7(4)
N(1)-C(7)-H(7A)	109.1	O(3)-C(21)-H(21A)	109.5
C(8)-C(7)-H(7A)	109.1	O(3)-C(21)-H(21B)	109.5
N(1)-C(7)-H(7B)	109.1	H(21A)-C(21)-H(21B)	109.5
C(8)-C(7)-H(7B)	109.1	O(3)-C(21)-H(21C)	109.5
H(7A)-C(7)-H(7B)	107.9	H(21A)-C(21)-H(21C)	109.5
C(13)-C(8)-C(9)	117.1(4)	H(21B)-C(21)-H(21C)	109.5
C(13)-C(8)-C(7)	122.0(3)	O(4)-C(22)-O(5)	124.8(5)
C(9)-C(8)-C(7)	121.0(4)	O(4)-C(22)-C(3)	125.4(5)
C(10)-C(9)-C(8)	121.4(4)	O(5)-C(22)-C(3)	109.8(3)
C(10)-C(9)-H(9A)	119.3	O(5)-C(23)-H(23A)	109.5
C(8)-C(9)-H(9A)	119.3	O(5)-C(23)-H(23B)	109.5
C(11)-C(10)-C(9)	118.4(4)	H(23A)-C(23)-H(23B)	109.5
C(11)-C(10)-H(10A)	120.8	O(5)-C(23)-H(23C)	109.5
C(9)-C(10)-H(10A)	120.8	H(23A)-C(23)-H(23C)	109.5
C(10)-C(11)-C(12)	122.4(5)	H(23B)-C(23)-H(23C)	109.5
C(10)-C(11)-Br(1)	119.5(4)	C(25A)-C(24)-C(25)	51(2)
C(12)-C(11)-Br(1)	118.1(4)	C(25A)-C(24)-C(5)	157(3)
C(11)-C(12)-C(13)	118.4(5)	C(25)-C(24)-C(5)	131(2)
C(11)-C(12)-H(12A)	120.8	C(24)-C(25)-H(25A)	120.0
C(13)-C(12)-H(12A)	120.8	C(24)-C(25)-H(25B)	120.0
C(12)-C(13)-C(8)	122.3(4)	H(25A)-C(25)-H(25B)	120.0
C(12)-C(13)-H(13A)	118.9	C(24)-C(25A)-H(25C)	120.0
C(8)-C(13)-H(13A)	118.9	C(24)-C(25A)-H(25D)	120.0
C(19)-C(14)-C(15)	116.5(4)	H(25C)-C(25A)-H(25D)	120.0
C(19)-C(14)-C(2)	122.2(3)	C(31)-C(26)-C(27)	111.6(3)
C(15)-C(14)-C(2)	121.2(4)	C(31)-C(26)-C(30)	113.3(3)
C(16)-C(15)-C(14)	121.0(6)	C(27)-C(26)-C(30)	101.8(3)
C(16)-C(15)-Cl(1)	118.0(5)	C(31)-C(26)-H(26A)	110.0
C(14)-C(15)-Cl(1)	121.0(4)	C(27)-C(26)-H(26A)	110.0
C(17)-C(16)-C(15)	120.1(6)	C(30)-C(26)-H(26A)	110.0
C(17)-C(16)-H(16A)	120.0	C(39)-C(27)-C(26)	115.5(3)
C(15)-C(16)-H(16A)	120.0	C(39)-C(27)-C(28)	117.6(3)
C(18)-C(17)-C(16)	119.2(6)	C(26)-C(27)-C(28)	103.3(3)
C(18)-C(17)-H(17A)	120.4	C(39)-C(27)-H(27A)	106.6
C(16)-C(17)-H(17A)	120.4	C(26)-C(27)-H(27A)	106.6
C(17)-C(18)-C(19)	122.2(6)	C(28)-C(27)-H(27A)	106.6
C(17)-C(18)-H(18A)	118.9	C(47)-C(28)-C(45)	109.6(4)
C(19)-C(18)-H(18A)	118.9	C(47)-C(28)-C(29)	112.0(4)
C(14)-C(19)-C(18)	121.0(5)	C(45)-C(28)-C(29)	108.8(3)
C(14)-C(19)-H(19A)	119.5	C(47)-C(28)-C(27)	111.9(3)
C(18)-C(19)-H(19A)	119.5	C(45)-C(28)-C(27)	110.8(3)
O(2)-C(20)-O(3)	124.4(4)	C(29)-C(28)-C(27)	103.6(3)

C(30)-C(29)-C(28)	107.5(3)
C(30)-C(29)-H(29A)	110.2
C(28)-C(29)-H(29A)	110.2
C(30)-C(29)-H(29B)	110.2
C(28)-C(29)-H(29B)	110.2
H(29A)-C(29)-H(29B)	108.5
C(49)-C(30)-C(29)	114.2(4)
C(49)-C(30)-C(26)	115.0(4)
C(29)-C(30)-C(26)	103.2(3)
C(49)-C(30)-H(30A)	108.0
C(29)-C(30)-H(30A)	108.0
C(26)-C(30)-H(30A)	108.0
O(6)-C(31)-N(2)	121.6(3)
O(6)-C(31)-C(26)	122.1(3)
N(2)-C(31)-C(26)	116.4(2)
N(2)-C(32)-C(33)	114.7(3)
N(2)-C(32)-H(32A)	108.6
C(33)-C(32)-H(32A)	108.6
N(2)-C(32)-H(32B)	108.6
C(33)-C(32)-H(32B)	108.6
H(32A)-C(32)-H(32B)	107.6
C(34)-C(33)-C(38)	117.5(5)
C(34)-C(33)-C(32)	119.8(5)
C(38)-C(33)-C(32)	122.7(4)
C(33)-C(34)-C(35)	120.6(6)
C(33)-C(34)-H(34A)	119.7
C(35)-C(34)-H(34A)	119.7
C(34)-C(35)-C(36)	118.5(7)
C(34)-C(35)-H(35A)	120.7
C(36)-C(35)-H(35A)	120.7
C(37)-C(36)-C(35)	121.1(7)
C(37)-C(36)-Br(2)	122.3(6)
C(35)-C(36)-Br(2)	115.8(6)
C(37)-C(36)-Br(2A)	114.6(7)
C(35)-C(36)-Br(2A)	120.7(6)
Br(2)-C(36)-Br(2A)	29.1(3)
C(36)-C(37)-C(38)	121.2(6)
C(36)-C(37)-H(37A)	119.4
C(38)-C(37)-H(37A)	119.4
C(37)-C(38)-C(33)	121.0(5)
C(37)-C(38)-H(38A)	119.5
C(33)-C(38)-H(38A)	119.5
C(40)-C(39)-C(44)	116.3(4)
C(40)-C(39)-C(27)	122.8(3)
C(44)-C(39)-C(27)	120.9(4)
C(39)-C(40)-C(41)	123.4(5)
C(39)-C(40)-Cl(2)	120.9(3)
C(41)-C(40)-Cl(2)	115.7(5)
C(42)-C(41)-C(40)	119.0(6)
C(42)-C(41)-H(41A)	120.5
C(40)-C(41)-H(41A)	120.5
C(41)-C(42)-C(43)	120.6(5)
C(41)-C(42)-H(42A)	119.7
C(43)-C(42)-H(42A)	119.7
C(42)-C(43)-C(44)	120.3(6)
C(42)-C(43)-H(43A)	119.9
C(44)-C(43)-H(43A)	119.9
C(39)-C(44)-C(43)	120.1(6)
C(39)-C(44)-H(44A)	120.0
C(43)-C(44)-H(44A)	120.0
O(7)-C(45)-O(8)	125.4(4)
O(7)-C(45)-C(28)	125.6(4)
O(8)-C(45)-C(28)	108.9(4)
O(8)-C(46)-H(46A)	109.5
O(8)-C(46)-H(46B)	109.5
H(46A)-C(46)-H(46B)	109.5
O(8)-C(46)-H(46C)	109.5
H(46A)-C(46)-H(46C)	109.5
H(46B)-C(46)-H(46C)	109.5
O(9)-C(47)-O(10)	124.1(6)
O(9)-C(47)-C(28)	124.5(5)
O(10)-C(47)-C(28)	111.3(4)
O(10)-C(48)-H(48A)	109.5
O(10)-C(48)-H(48B)	109.5
H(48A)-C(48)-H(48B)	109.5
O(10)-C(48)-H(48C)	109.5
H(48A)-C(48)-H(48C)	109.5
H(48B)-C(48)-H(48C)	109.5
C(50)-C(49)-C(30)	126.3(9)
C(50)-C(49)-H(49A)	116.9
C(30)-C(49)-H(49A)	116.9
C(49)-C(50)-H(50A)	120.0
C(49)-C(50)-H(50C)	120.0
H(50A)-C(50)-H(50C)	120.0

Table 4. Torsion angles [deg] for 175.

C(6)-C(1)-C(2)-C(14)	66.8(3)
C(5)-C(1)-C(2)-C(14)	-173.1(3)
C(6)-C(1)-C(2)-C(3)	-160.2(2)
C(5)-C(1)-C(2)-C(3)	-40.0(3)
C(14)-C(2)-C(3)-C(20)	47.8(5)
C(1)-C(2)-C(3)-C(20)	-82.6(4)
C(14)-C(2)-C(3)-C(22)	-75.7(4)
C(1)-C(2)-C(3)-C(22)	153.9(3)
C(14)-C(2)-C(3)-C(4)	165.7(4)
C(1)-C(2)-C(3)-C(4)	35.3(4)
C(20)-C(3)-C(4)-C(5)	105.4(4)
C(22)-C(3)-C(4)-C(5)	-134.4(4)
C(2)-C(3)-C(4)-C(5)	-17.6(5)
C(3)-C(4)-C(5)-C(24)	-133.1(6)
C(3)-C(4)-C(5)-C(1)	-6.6(5)
C(6)-C(1)-C(5)-C(24)	-85.1(6)
C(2)-C(1)-C(5)-C(24)	156.2(6)
C(6)-C(1)-C(5)-C(4)	147.6(3)
C(2)-C(1)-C(5)-C(4)	28.9(4)
C(7)-N(1)-C(6)-O(1)	-3.9(6)
C(7)-N(1)-C(6)-C(1)	175.3(3)
C(2)-C(1)-C(6)-O(1)	57.8(4)
C(5)-C(1)-C(6)-O(1)	-57.0(4)
C(2)-C(1)-C(6)-N(1)	-121.4(3)
C(5)-C(1)-C(6)-N(1)	123.8(3)
C(6)-N(1)-C(7)-C(8)	-97.3(4)
N(1)-C(7)-C(8)-C(13)	42.2(4)
N(1)-C(7)-C(8)-C(9)	-137.6(3)
C(13)-C(8)-C(9)-C(10)	-0.5(6)
C(7)-C(8)-C(9)-C(10)	179.4(4)
C(8)-C(9)-C(10)-C(11)	0.2(7)
C(9)-C(10)-C(11)-C(12)	-0.3(7)
C(9)-C(10)-C(11)-Br(1)	179.2(4)
C(10)-C(11)-C(12)-C(13)	0.6(8)
Br(1)-C(11)-C(12)-C(13)	-178.9(4)
C(11)-C(12)-C(13)-C(8)	-0.9(8)
C(9)-C(8)-C(13)-C(12)	0.8(6)
C(7)-C(8)-C(13)-C(12)	-179.1(4)
C(1)-C(2)-C(14)-C(19)	40.1(5)
C(3)-C(2)-C(14)-C(19)	-84.9(4)

C(1)-C(2)-C(14)-C(15)	-135.3(4)
C(3)-C(2)-C(14)-C(15)	99.7(4)
C(19)-C(14)-C(15)-C(16)	2.3(6)
C(2)-C(14)-C(15)-C(16)	177.9(4)
C(19)-C(14)-C(15)-Cl(1)	-177.6(3)
C(2)-C(14)-C(15)-Cl(1)	-2.0(6)
C(14)-C(15)-C(16)-C(17)	-0.2(8)
Cl(1)-C(15)-C(16)-C(17)	179.7(5)
C(15)-C(16)-C(17)-C(18)	-1.4(10)
C(16)-C(17)-C(18)-C(19)	1.0(10)
C(15)-C(14)-C(19)-C(18)	-2.8(6)
C(2)-C(14)-C(19)-C(18)	-178.3(4)
C(17)-C(18)-C(19)-C(14)	1.2(9)
C(21)-O(3)-C(20)-O(2)	0.2(11)
C(21)-O(3)-C(20)-C(3)	-176.5(7)
C(22)-C(3)-C(20)-O(2)	140.7(6)
C(4)-C(3)-C(20)-O(2)	-97.4(6)
C(2)-C(3)-C(20)-O(2)	17.1(8)
C(22)-C(3)-C(20)-O(3)	-42.7(6)
C(4)-C(3)-C(20)-O(3)	79.3(6)
C(2)-C(3)-C(20)-O(3)	-166.3(5)
C(23)-O(5)-C(22)-O(4)	-4.3(11)
C(23)-O(5)-C(22)-C(3)	177.9(8)
C(20)-C(3)-C(22)-O(4)	132.2(5)
C(4)-C(3)-C(22)-O(4)	12.2(7)
C(2)-C(3)-C(22)-O(4)	-100.3(6)
C(20)-C(3)-C(22)-O(5)	-50.1(5)
C(4)-C(3)-C(22)-O(5)	-170.1(4)
C(2)-C(3)-C(22)-O(5)	77.5(5)
C(4)-C(5)-C(24)-C(25A)	165(7)
C(1)-C(5)-C(24)-C(25A)	43(7)
C(4)-C(5)-C(24)-C(25)	-109(2)
C(1)-C(5)-C(24)-C(25)	129(2)
C(31)-C(26)-C(27)-C(39)	65.6(3)
C(30)-C(26)-C(27)-C(39)	-173.4(3)
C(31)-C(26)-C(27)-C(28)	-164.6(3)
C(30)-C(26)-C(27)-C(28)	-43.6(3)
C(39)-C(27)-C(28)-C(47)	34.8(5)
C(26)-C(27)-C(28)-C(47)	-93.7(4)
C(39)-C(27)-C(28)-C(45)	-87.8(4)
C(26)-C(27)-C(28)-C(45)	143.7(3)
C(39)-C(27)-C(28)-C(29)	155.7(3)
C(26)-C(27)-C(28)-C(29)	27.2(4)
C(47)-C(28)-C(29)-C(30)	120.5(4)

C(45)-C(28)-C(29)-C(30)	-118.2(4)
C(27)-C(28)-C(29)-C(30)	-0.3(4)
C(28)-C(29)-C(30)-C(49)	-151.9(4)
C(28)-C(29)-C(30)-C(26)	-26.4(4)
C(31)-C(26)-C(30)-C(49)	-71.6(5)
C(27)-C(26)-C(30)-C(49)	168.5(4)
C(31)-C(26)-C(30)-C(29)	163.3(3)
C(27)-C(26)-C(30)-C(29)	43.4(3)
C(32)-N(2)-C(31)-O(6)	-2.9(5)
C(32)-N(2)-C(31)-C(26)	176.3(3)
C(27)-C(26)-C(31)-O(6)	56.6(4)
C(30)-C(26)-C(31)-O(6)	-57.5(4)
C(27)-C(26)-C(31)-N(2)	-122.6(3)
C(30)-C(26)-C(31)-N(2)	123.3(3)
C(31)-N(2)-C(32)-C(33)	-81.9(4)
N(2)-C(32)-C(33)-C(34)	171.5(6)
N(2)-C(32)-C(33)-C(38)	-7.6(5)
C(38)-C(33)-C(34)-C(35)	-2.0(13)
C(32)-C(33)-C(34)-C(35)	178.8(9)
C(33)-C(34)-C(35)-C(36)	1.5(18)
C(34)-C(35)-C(36)-C(37)	-1.4(19)
C(34)-C(35)-C(36)-Br(2)	-171.4(9)
C(34)-C(35)-C(36)-Br(2A)	155.9(10)
C(35)-C(36)-C(37)-C(38)	1.8(15)
Br(2)-C(36)-C(37)-C(38)	171.1(6)
Br(2A)-C(36)-C(37)-C(38)	-156.8(7)
C(36)-C(37)-C(38)-C(33)	-2.3(11)
C(34)-C(33)-C(38)-C(37)	2.3(8)
C(32)-C(33)-C(38)-C(37)	-178.5(5)
C(26)-C(27)-C(39)-C(40)	-127.1(4)
C(28)-C(27)-C(39)-C(40)	110.4(4)
C(26)-C(27)-C(39)-C(44)	50.9(5)
C(28)-C(27)-C(39)-C(44)	-71.5(5)
C(44)-C(39)-C(40)-C(41)	6.5(6)
C(27)-C(39)-C(40)-C(41)	-175.3(4)
C(44)-C(39)-C(40)-Cl(2)	-172.3(3)
C(27)-C(39)-C(40)-Cl(2)	5.8(5)
C(39)-C(40)-C(41)-C(42)	-3.5(8)
Cl(2)-C(40)-C(41)-C(42)	175.3(5)
C(40)-C(41)-C(42)-C(43)	-1.3(10)
C(41)-C(42)-C(43)-C(44)	2.8(10)
C(40)-C(39)-C(44)-C(43)	-4.9(7)
C(27)-C(39)-C(44)-C(43)	177.0(5)
C(42)-C(43)-C(44)-C(39)	0.5(9)

C(46)-O(8)-C(45)-O(7)	3.6(11)
C(46)-O(8)-C(45)-C(28)	-179.2(8)
C(47)-C(28)-C(45)-O(7)	-138.7(5)
C(29)-C(28)-C(45)-O(7)	98.5(5)
C(27)-C(28)-C(45)-O(7)	-14.8(6)
C(47)-C(28)-C(45)-O(8)	44.1(5)
C(29)-C(28)-C(45)-O(8)	-78.7(5)
C(27)-C(28)-C(45)-O(8)	168.0(4)
C(48)-O(10)-C(47)-O(9)	-5.5(11)
C(48)-O(10)-C(47)-C(28)	173.1(8)
C(45)-C(28)-C(47)-O(9)	-137.2(5)
C(29)-C(28)-C(47)-O(9)	-16.4(7)
C(27)-C(28)-C(47)-O(9)	99.5(6)
C(45)-C(28)-C(47)-O(10)	44.2(5)
C(29)-C(28)-C(47)-O(10)	165.0(4)
C(27)-C(28)-C(47)-O(10)	-79.1(5)
C(29)-C(30)-C(49)-C(50)	-111.3(10)
C(26)-C(30)-C(49)-C(50)	129.7(9)

Table 5. Hydrogen bonds for 175 [A and deg.].

D-H...A	d(D-H)	d(H...A)	d(D...A)	<(DHA)
N(1)-H(1A)...O(6)	0.86	1.99	2.834(3)	167.9
N(2)-H(2A)...O(1)#1	0.86	2.03	2.878(3)	166.5

Symmetry transformations used to generate equivalent atoms:
#1 x+1,y,z

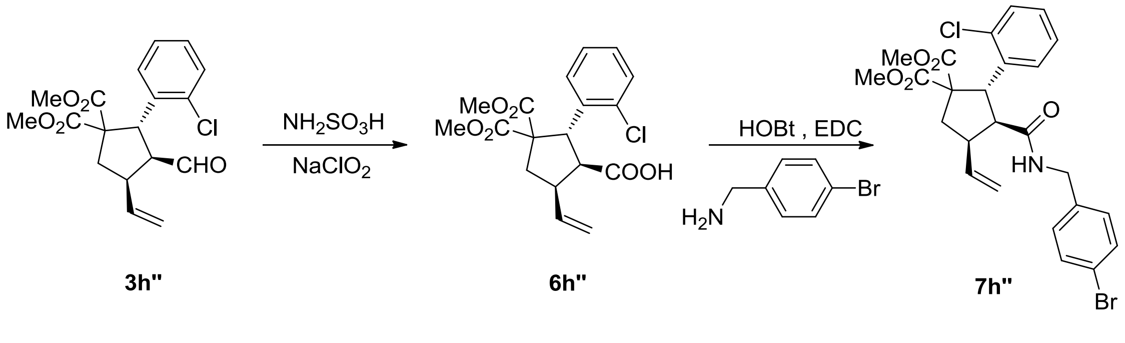

(2*R*,3*S*,4*R*)-Dimethyl 3-((4-bromobenzyl)carbamoyl)-2-(2-chlorophenyl)-4-vinylcyclopentane-1,1-dicarboxylate (7h'') The title compound was prepared following the procedure for the synthesis of **7h''** in 49% yield. ^{1}H NMR (400 MHz, $CDCl_3$): δ 7.12-7.35 (m, 6H), 6.90-6.92 (m, 2H), 5.95 (m, 1H), 5.82-5.89 (m, 1H), 5.09-5.18 (m, 3H), 4.35-4.39 (m, 1H), 4.21-4.25 (m, 1H), 3.77 (s, 3H), 3.18 (s, 3H), 2.91-2.97 (m, 1H), 2.78 (m, 1H), 2.68-2.73 (m, 1H), 2.41-2.45 (m, 1H); ^{13}C NMR (100 MHz,

$CDCl_3$): δ 172.11, 171.24, 169.97, 137.80, 137.43, 137.18, 135.45, 131.46, 129.78, 128.98, 128.66, 128.41, 126.70, 120.96, 116.77, 64.30, 59.84, 53.18, 52.02, 49.32, 45.73, 42.66, 40.49. HRMS (EI) m/z calcd for $C_{25}H_{25}BrClNO_5$ (M): 533.0605, found 533.0614; HPLC (Chiralpak OD-3, *i*PrOH/hexane = 15/85, flow rate = 1mL/min, λ = 210 nm): t_{major} = 18.45min, t_{minor} = 9.47min, ee = 84%

X-ray structure of compound 7h''

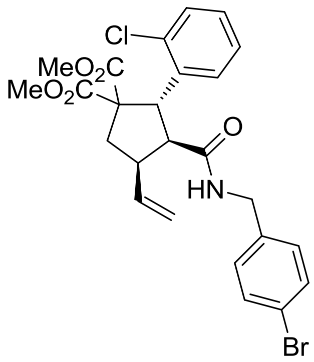

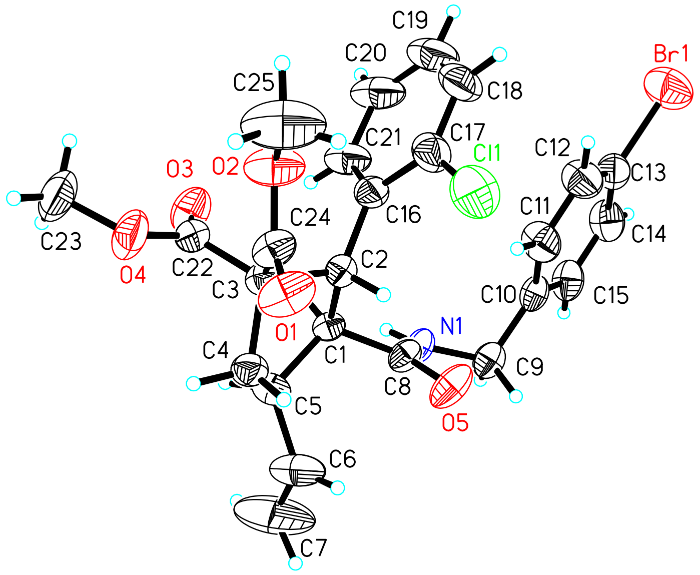

Table 6. Crystal data and structure refinement for 171.

Identification code	171
Empirical formula	C25 H25 Br Cl N O5
Formula weight	534.82
Temperature	296(2) K
Wavelength	1.54178 A
Crystal system, space group	Triclinic, P 1
Unit cell dimensions	a = 9.582 A alpha = 103.71 deg.
	b = 11.863 A beta = 108.33 deg.
	c = 13.061 A gamma = 102.90 deg.
Volume	1295.9 A^3
Z, Calculated density	2, 1.371 Mg/m^3

Absorption coefficient	3.395 mm^-1
F(000)	548
Crystal size	0.35 x 0.10 x 0.10 mm
Theta range for data collection	3.78 to 64.99 deg.
Limiting indices	-9<=h<=10, -13<=k<=12, -15<=l<=14
Reflections collected / unique	7340 / 5182 [R(int) = 0.0183]
Completeness to theta = 64.99	89.5 %
Absorption correction	Semi-empirical from equivalents
Max. and min. transmission	0.7529 and 0.5302
Refinement method	Full-matrix least-squares on F^2
Data / restraints / parameters	5182 / 13 / 605
Goodness-of-fit on F^2	1.061
Final R indices [I>2sigma(I)]	R1 = 0.0526, wR2 = 0.1483
R indices (all data)	R1 = 0.0543, wR2 = 0.1511
Absolute structure parameter	0.04(2)
Largest diff. peak and hole	0.315 and -0.531 e.A^-3

Table 7. Atomic coordinates (x 10^4) and equivalent isotropic displacement parameters (A^2 x 10^3) for 171.

	x	y	z	U(eq)
Br(1)	8607(1)	8168(1)	9200(1)	127(1)
Br(2)	3881(8)	-775(3)	-1665(3)	235(3)
Br(2A)	5046(3)	-643(2)	-2206(2)	129(2)
Cl(1)	2708(4)	7097(2)	3577(3)	136(1)
Cl(2)	5773(2)	619(2)	3434(2)	99(1)
O(1)	843(5)	5517(5)	416(5)	93(1)
O(2)	3125(6)	6993(4)	1137(5)	97(2)
O(3)	5802(6)	5384(5)	1041(4)	90(1)
O(4)	3872(6)	5353(6)	-453(4)	107(2)
O(5)	2725(5)	3545(4)	3476(4)	83(1)
O(6)	12774(8)	2667(6)	6593(6)	119(2)
O(7)	11053(10)	790(6)	6009(9)	198(6)
O(8)	7631(9)	1474(5)	6190(6)	107(2)
O(9)	9858(13)	1710(10)	7579(5)	252(8)
O(10)	7900(5)	3831(4)	3648(4)	68(1)
N(1)	5129(5)	3473(4)	4094(3)	55(1)
N(2)	9756(5)	3899(4)	2945(4)	57(1)
C(1)	4229(6)	3958(4)	2366(4)	50(1)
C(2)	3823(6)	5143(4)	2340(4)	50(1)
C(3)	3220(6)	5025(4)	1045(4)	56(1)
C(4)	2244(7)	3656(5)	499(4)	65(1)
C(5)	3182(7)	3000(5)	1161(5)	66(1)
C(6)	2192(10)	1826(6)	1151(7)	105(3)
C(7)	2327(19)	815(9)	895(15)	193(8)
C(8)	3970(6)	3626(4)	3357(4)	54(1)
C(9)	5155(7)	3311(5)	5160(5)	65(1)
C(10)	5989(6)	4499(5)	6159(4)	60(1)
C(11)	5762(9)	5580(6)	6063(5)	86(2)
C(12)	6548(10)	6669(7)	6972(6)	92(2)
C(13)	7562(8)	6662(6)	7959(5)	76(2)
C(14)	7826(9)	5627(7)	8100(6)	87(2)
C(15)	7023(8)	4537(7)	7182(5)	77(2)
C(16)	5055(7)	6301(4)	3236(4)	62(1)
C(17)	4657(10)	7222(6)	3855(6)	87(2)
C(18)	5771(16)	8259(6)	4696(7)	110(3)
C(19)	7310(16)	8381(7)	4991(7)	120(4)
C(20)	7704(11)	7503(7)	4407(8)	112(3)
C(21)	6596(8)	6474(6)	3539(6)	83(2)
C(22)	4486(7)	5287(5)	580(5)	66(1)

C(23)	4913(13)	5602(11)	-1028(8)	133(4)
C(24)	2244(7)	5850(5)	802(5)	68(1)
C(25)	2335(14)	7871(9)	930(13)	158(5)
C(26)	10202(5)	3430(4)	4693(4)	52(1)
C(27)	9210(6)	2312(4)	4846(4)	53(1)
C(28)	10072(8)	2444(5)	6119(4)	77(2)
C(29)	10536(9)	3845(5)	6701(5)	83(2)
C(30)	10968(7)	4457(5)	5889(5)	67(1)
C(31)	10566(13)	5595(6)	5951(6)	109(3)
C(32)	11390(20)	6622(10)	6223(16)	242(12)
C(33)	9191(5)	3749(4)	3735(4)	49(1)
C(34)	8846(7)	4065(5)	1900(5)	64(1)
C(35)	7783(8)	2888(6)	967(5)	73(1)
C(36)	7662(11)	1743(7)	1067(6)	100(2)
C(37)	6710(14)	709(8)	203(8)	123(3)
C(38)	5832(19)	770(10)	-722(10)	225(9)
C(39)	5950(30)	1888(13)	-926(12)	303(17)
C(40)	6850(20)	2930(9)	-27(9)	186(7)
C(41)	8863(7)	1105(5)	3927(4)	61(1)
C(42)	7381(7)	280(5)	3294(5)	68(1)
C(43)	7107(10)	-840(6)	2477(5)	89(2)
C(44)	8277(14)	-1130(7)	2263(8)	109(3)
C(45)	9727(15)	-301(8)	2779(10)	125(4)
C(46)	10045(10)	796(7)	3643(8)	94(2)
C(47)	11465(13)	2025(7)	6295(7)	115(3)
C(48)	12350(20)	307(14)	6090(30)	360(20)
C(49)	9000(14)	1830(6)	6608(5)	112(3)
C(50)	8980(30)	1162(18)	8161(9)	370(20)
H(1A)	5934	3468	3935	66
H(2A)	10689	3897	3057	68
H(1B)	5322	4103	2478	60
H(2B)	2911	5065	2539	60
H(4A)	1243	3524	562	78
H(4B)	2078	3363	-303	78
H(5A)	3872	2794	784	79
H(6A)	1367	1876	1369	126
H(7A)	3130	704	669	232
H(7B)	1628	151	924	232
H(9A)	5669	2713	5303	77
H(9B)	4097	2991	5099	77
H(11A)	5071	5580	5379	103
H(12A)	6379	7395	6903	110
H(14A)	8522	5640	8788	104
H(15A)	7193	3816	7264	92

H(18A)	5474	8878	5066	132
H(19A)	8068	9060	5583	144
H(20A)	8750	7587	4589	134
H(21A)	6916	5884	3153	99
H(23A)	4341	5626	-1767	199
H(23B)	5381	4968	-1114	199
H(23C)	5709	6378	-583	199
H(25A)	3086	8672	1200	237
H(25B)	1646	7878	1328	237
H(25C)	1748	7646	125	237
H(26A)	11025	3208	4484	62
H(27A)	8210	2436	4774	64
H(29A)	9676	4054	6842	99
H(29B)	11414	4116	7426	99
H(30A)	12096	4676	6120	80
H(31A)	9514	5497	5748	130
H(32A)	12457	6790	6437	290
H(32B)	10967	7255	6221	290
H(34A)	9549	4502	1623	77
H(34B)	8230	4571	2077	77
H(36A)	8258	1684	1756	120
H(37A)	6697	-50	286	147
H(39A)	5430	1916	-1648	364
H(40A)	6813	3686	-100	223
H(43A)	6100	-1385	2082	106
H(44A)	8100	-1898	1762	131
H(45A)	10516	-460	2559	151
H(46A)	11061	1325	4032	112
H(48A)	11969	-572	5846	545
H(48B)	13089	619	6867	545
H(48C)	12846	557	5608	545
H(50A)	9678	1188	8884	548
H(50B)	8381	325	7700	548
H(50C)	8291	1609	8283	548

Table 8. Bond lengths [A] and angles [deg] for 171.

Br(1)-C(13)	1.906(6)	C(6)-H(6A)	0.9300
Br(2)-C(38)	2.079(13)	C(7)-H(7A)	0.9300
Br(2A)-C(38)	2.028(10)	C(7)-H(7B)	0.9300
Cl(1)-C(17)	1.750(9)	C(9)-C(10)	1.515(8)
Cl(2)-C(42)	1.730(7)	C(9)-H(9A)	0.9700
O(1)-C(24)	1.206(7)	C(9)-H(9B)	0.9700
O(2)-C(24)	1.311(7)	C(10)-C(15)	1.374(9)
O(2)-C(25)	1.445(9)	C(10)-C(11)	1.376(8)
O(3)-C(22)	1.180(8)	C(11)-C(12)	1.387(10)
O(4)-C(22)	1.321(8)	C(11)-H(11A)	0.9300
O(4)-C(23)	1.446(9)	C(12)-C(13)	1.352(10)
O(5)-C(8)	1.237(6)	C(12)-H(12A)	0.9300
O(6)-C(47)	1.196(12)	C(13)-C(14)	1.350(10)
O(7)-C(47)	1.353(10)	C(14)-C(15)	1.394(10)
O(7)-C(48)	1.47(2)	C(14)-H(14A)	0.9300
O(8)-C(49)	1.180(12)	C(15)-H(15A)	0.9300
O(9)-C(49)	1.337(13)	C(16)-C(21)	1.356(9)
O(9)-C(50)	1.450(16)	C(16)-C(17)	1.397(8)
O(10)-C(33)	1.234(6)	C(17)-C(18)	1.377(12)
N(1)-C(8)	1.301(7)	C(18)-C(19)	1.366(15)
N(1)-C(9)	1.444(7)	C(18)-H(18A)	0.9300
N(1)-H(1A)	0.8600	C(19)-C(20)	1.338(16)
N(2)-C(33)	1.336(6)	C(19)-H(19A)	0.9300
N(2)-C(34)	1.454(7)	C(20)-C(21)	1.382(10)
N(2)-H(2A)	0.8600	C(20)-H(20A)	0.9300
C(1)-C(8)	1.514(7)	C(21)-H(21A)	0.9300
C(1)-C(2)	1.545(6)	C(23)-H(23A)	0.9600
C(1)-C(5)	1.557(7)	C(23)-H(23B)	0.9600
C(1)-H(1B)	0.9800	C(23)-H(23C)	0.9600
C(2)-C(16)	1.515(7)	C(25)-H(25A)	0.9600
C(2)-C(3)	1.565(6)	C(25)-H(25B)	0.9600
C(2)-H(2B)	0.9800	C(25)-H(25C)	0.9600
C(3)-C(24)	1.517(8)	C(26)-C(33)	1.507(7)
C(3)-C(22)	1.524(8)	C(26)-C(27)	1.544(6)
C(3)-C(4)	1.541(7)	C(26)-C(30)	1.562(7)
C(4)-C(5)	1.518(8)	C(26)-H(26A)	0.9800
C(4)-H(4A)	0.9700	C(27)-C(41)	1.524(7)
C(4)-H(4B)	0.9700	C(27)-C(28)	1.558(7)
C(5)-C(6)	1.495(9)	C(27)-H(27A)	0.9800
C(5)-H(5A)	0.9800	C(28)-C(47)	1.497(14)
C(6)-C(7)	1.214(15)	C(28)-C(49)	1.514(11)

C(28)-C(29)	1.547(9)
C(29)-C(30)	1.526(8)
C(29)-H(29A)	0.9700
C(29)-H(29B)	0.9700
C(30)-C(31)	1.476(10)
C(30)-H(30A)	0.9800
C(31)-C(32)	1.193(16)
C(31)-H(31A)	0.9300
C(32)-H(32A)	0.9300
C(32)-H(32B)	0.9300
C(34)-C(35)	1.500(9)
C(34)-H(34A)	0.9700
C(34)-H(34B)	0.9700
C(35)-C(40)	1.346(11)
C(35)-C(36)	1.378(10)
C(36)-C(37)	1.344(12)
C(36)-H(36A)	0.9300
C(37)-C(38)	1.267(15)
C(37)-H(37A)	0.9300
C(38)-C(39)	1.401(19)
C(39)-C(40)	1.361(15)
C(39)-H(39A)	0.9300
C(40)-H(40A)	0.9300
C(41)-C(42)	1.384(8)
C(41)-C(46)	1.393(9)
C(42)-C(43)	1.403(8)
C(43)-C(44)	1.334(13)
C(43)-H(43A)	0.9300
C(44)-C(45)	1.356(15)
C(44)-H(44A)	0.9300
C(45)-C(46)	1.404(12)
C(45)-H(45A)	0.9300
C(46)-H(46A)	0.9300
C(48)-H(48A)	0.9600
C(48)-H(48B)	0.9600
C(48)-H(48C)	0.9600
C(50)-H(50A)	0.9600
C(50)-H(50B)	0.9600
C(50)-H(50C)	0.9600

C(24)-O(2)-C(25)	116.4(6)
C(22)-O(4)-C(23)	116.9(6)
C(47)-O(7)-C(48)	114.1(11)
C(49)-O(9)-C(50)	114.9(14)
C(8)-N(1)-C(9)	124.4(4)
C(8)-N(1)-H(1A)	117.8
C(9)-N(1)-H(1A)	117.8
C(33)-N(2)-C(34)	122.3(4)
C(33)-N(2)-H(2A)	118.9
C(34)-N(2)-H(2A)	118.9
C(8)-C(1)-C(2)	108.4(4)
C(8)-C(1)-C(5)	115.2(4)
C(2)-C(1)-C(5)	105.1(4)
C(8)-C(1)-H(1B)	109.3
C(2)-C(1)-H(1B)	109.3
C(5)-C(1)-H(1B)	109.3
C(16)-C(2)-C(1)	114.2(4)
C(16)-C(2)-C(3)	120.7(4)
C(1)-C(2)-C(3)	104.5(4)
C(16)-C(2)-H(2B)	105.4
C(1)-C(2)-H(2B)	105.4
C(3)-C(2)-H(2B)	105.4
C(24)-C(3)-C(22)	108.4(4)
C(24)-C(3)-C(4)	112.2(4)
C(22)-C(3)-C(4)	109.6(4)
C(24)-C(3)-C(2)	111.4(4)
C(22)-C(3)-C(2)	114.7(4)
C(4)-C(3)-C(2)	100.4(4)
C(5)-C(4)-C(3)	105.5(4)
C(5)-C(4)-H(4A)	110.6
C(3)-C(4)-H(4A)	110.6
C(5)-C(4)-H(4B)	110.6
C(3)-C(4)-H(4B)	110.6
H(4A)-C(4)-H(4B)	108.8
C(6)-C(5)-C(4)	112.8(5)
C(6)-C(5)-C(1)	115.6(5)
C(4)-C(5)-C(1)	106.3(4)
C(6)-C(5)-H(5A)	107.2
C(4)-C(5)-H(5A)	107.2
C(1)-C(5)-H(5A)	107.2
C(7)-C(6)-C(5)	128.3(13)
C(7)-C(6)-H(6A)	115.8
C(5)-C(6)-H(6A)	115.8
C(6)-C(7)-H(7A)	120.0
C(6)-C(7)-H(7B)	120.0
H(7A)-C(7)-H(7B)	120.0

O(5)-C(8)-N(1)	121.8(5)
O(5)-C(8)-C(1)	121.1(5)
N(1)-C(8)-C(1)	117.1(4)
N(1)-C(9)-C(10)	112.2(4)
N(1)-C(9)-H(9A)	109.2
C(10)-C(9)-H(9A)	109.2
N(1)-C(9)-H(9B)	109.2
C(10)-C(9)-H(9B)	109.2
H(9A)-C(9)-H(9B)	107.9
C(15)-C(10)-C(11)	117.8(6)
C(15)-C(10)-C(9)	121.1(5)
C(11)-C(10)-C(9)	121.1(5)
C(10)-C(11)-C(12)	120.9(6)
C(10)-C(11)-H(11A)	119.5
C(12)-C(11)-H(11A)	119.5
C(13)-C(12)-C(11)	119.1(6)
C(13)-C(12)-H(12A)	120.4
C(11)-C(12)-H(12A)	120.4
C(14)-C(13)-C(12)	122.4(6)
C(14)-C(13)-Br(1)	119.4(5)
C(12)-C(13)-Br(1)	118.2(5)
C(13)-C(14)-C(15)	117.9(6)
C(13)-C(14)-H(14A)	121.0
C(15)-C(14)-H(14A)	121.0
C(10)-C(15)-C(14)	121.9(6)
C(10)-C(15)-H(15A)	119.1
C(14)-C(15)-H(15A)	119.1
C(21)-C(16)-C(17)	116.0(6)
C(21)-C(16)-C(2)	122.5(5)
C(17)-C(16)-C(2)	121.3(6)
C(18)-C(17)-C(16)	121.7(9)
C(18)-C(17)-Cl(1)	117.5(7)
C(16)-C(17)-Cl(1)	120.8(6)
C(19)-C(18)-C(17)	120.2(8)
C(19)-C(18)-H(18A)	119.9
C(17)-C(18)-H(18A)	119.9
C(20)-C(19)-C(18)	118.5(8)
C(20)-C(19)-H(19A)	120.7
C(18)-C(19)-H(19A)	120.7
C(19)-C(20)-C(21)	121.7(10)
C(19)-C(20)-H(20A)	119.2
C(21)-C(20)-H(20A)	119.2
C(16)-C(21)-C(20)	121.8(8)
C(16)-C(21)-H(21A)	119.1
C(20)-C(21)-H(21A)	119.1
O(3)-C(22)-O(4)	124.4(5)
O(3)-C(22)-C(3)	126.4(5)
O(4)-C(22)-C(3)	109.2(5)
O(4)-C(23)-H(23A)	109.5
O(4)-C(23)-H(23B)	109.5
H(23A)-C(23)-H(23B)	109.5
O(4)-C(23)-H(23C)	109.5
H(23A)-C(23)-H(23C)	109.5
H(23B)-C(23)-H(23C)	109.5
O(1)-C(24)-O(2)	124.2(6)
O(1)-C(24)-C(3)	124.8(5)
O(2)-C(24)-C(3)	110.9(5)
O(2)-C(25)-H(25A)	109.5
O(2)-C(25)-H(25B)	109.5
H(25A)-C(25)-H(25B)	109.5
O(2)-C(25)-H(25C)	109.5
H(25A)-C(25)-H(25C)	109.5
H(25B)-C(25)-H(25C)	109.5
C(33)-C(26)-C(27)	109.1(4)
C(33)-C(26)-C(30)	115.6(4)
C(27)-C(26)-C(30)	106.2(4)
C(33)-C(26)-H(26A)	108.6
C(27)-C(26)-H(26A)	108.6
C(30)-C(26)-H(26A)	108.6
C(41)-C(27)-C(26)	113.2(4)
C(41)-C(27)-C(28)	118.2(4)
C(26)-C(27)-C(28)	103.9(4)
C(41)-C(27)-H(27A)	107.0
C(26)-C(27)-H(27A)	107.0
C(28)-C(27)-H(27A)	107.0
C(47)-C(28)-C(49)	111.7(7)
C(47)-C(28)-C(29)	111.7(6)
C(49)-C(28)-C(29)	107.2(6)
C(47)-C(28)-C(27)	112.2(6)
C(49)-C(28)-C(27)	112.5(6)
C(29)-C(28)-C(27)	100.9(5)
C(30)-C(29)-C(28)	107.1(5)
C(30)-C(29)-H(29A)	110.3
C(28)-C(29)-H(29A)	110.3
C(30)-C(29)-H(29B)	110.3
C(28)-C(29)-H(29B)	110.3
H(29A)-C(29)-H(29B)	108.6
C(31)-C(30)-C(29)	112.4(6)

C(31)-C(30)-C(26)	116.3(5)
C(29)-C(30)-C(26)	105.1(4)
C(31)-C(30)-H(30A)	107.6
C(29)-C(30)-H(30A)	107.6
C(26)-C(30)-H(30A)	107.6
C(32)-C(31)-C(30)	129.6(14)
C(32)-C(31)-H(31A)	115.2
C(30)-C(31)-H(31A)	115.2
C(31)-C(32)-H(32A)	120.0
C(31)-C(32)-H(32B)	120.0
H(32A)-C(32)-H(32B)	120.0
O(10)-C(33)-N(2)	121.0(5)
O(10)-C(33)-C(26)	123.3(4)
N(2)-C(33)-C(26)	115.6(4)
N(2)-C(34)-C(35)	113.9(5)
N(2)-C(34)-H(34A)	108.8
C(35)-C(34)-H(34A)	108.8
N(2)-C(34)-H(34B)	108.8
C(35)-C(34)-H(34B)	108.8
H(34A)-C(34)-H(34B)	107.7
C(40)-C(35)-C(36)	116.7(7)
C(40)-C(35)-C(34)	119.1(6)
C(36)-C(35)-C(34)	124.1(6)
C(37)-C(36)-C(35)	122.2(7)
C(37)-C(36)-H(36A)	118.9
C(35)-C(36)-H(36A)	118.9
C(38)-C(37)-C(36)	120.0(9)
C(38)-C(37)-H(37A)	120.0
C(36)-C(37)-H(37A)	120.0
C(37)-C(38)-C(39)	121.5(10)
C(37)-C(38)-Br(2A)	120.6(9)
C(39)-C(38)-Br(2A)	111.0(10)
C(37)-C(38)-Br(2)	114.1(10)
C(39)-C(38)-Br(2)	122.2(10)
Br(2A)-C(38)-Br(2)	42.7(3)
C(40)-C(39)-C(38)	117.4(10)
C(40)-C(39)-H(39A)	121.3
C(38)-C(39)-H(39A)	121.3
C(35)-C(40)-C(39)	121.2(9)
C(35)-C(40)-H(40A)	119.4
C(39)-C(40)-H(40A)	119.4
C(42)-C(41)-C(46)	115.8(5)
C(42)-C(41)-C(27)	123.4(5)
C(46)-C(41)-C(27)	120.8(5)
C(41)-C(42)-C(43)	121.9(6)
C(41)-C(42)-Cl(2)	121.0(4)
C(43)-C(42)-Cl(2)	117.0(5)
C(44)-C(43)-C(42)	120.5(8)
C(44)-C(43)-H(43A)	119.7
C(42)-C(43)-H(43A)	119.7
C(43)-C(44)-C(45)	119.7(7)
C(43)-C(44)-H(44A)	120.1
C(45)-C(44)-H(44A)	120.1
C(44)-C(45)-C(46)	120.4(8)
C(44)-C(45)-H(45A)	119.8
C(46)-C(45)-H(45A)	119.8
C(41)-C(46)-C(45)	121.1(8)
C(41)-C(46)-H(46A)	119.5
C(45)-C(46)-H(46A)	119.5
O(6)-C(47)-O(7)	123.0(11)
O(6)-C(47)-C(28)	125.8(7)
O(7)-C(47)-C(28)	111.1(8)
O(7)-C(48)-H(48A)	109.5
O(7)-C(48)-H(48B)	109.5
H(48A)-C(48)-H(48B)	109.5
O(7)-C(48)-H(48C)	109.5
H(48A)-C(48)-H(48C)	109.5
H(48B)-C(48)-H(48C)	109.5
O(8)-C(49)-O(9)	124.9(8)
O(8)-C(49)-C(28)	126.2(7)
O(9)-C(49)-C(28)	108.9(9)
O(9)-C(50)-H(50A)	109.5
O(9)-C(50)-H(50B)	109.5
H(50A)-C(50)-H(50B)	109.5
O(9)-C(50)-H(50C)	109.5
H(50A)-C(50)-H(50C)	109.5
H(50B)-C(50)-H(50C)	109.5

Table 9. Torsion angles [deg] for 171.

C(8)-C(1)-C(2)-C(16)	76.7(5)
C(5)-C(1)-C(2)-C(16)	-159.6(4)
C(8)-C(1)-C(2)-C(3)	-149.4(4)
C(5)-C(1)-C(2)-C(3)	-25.7(5)
C(16)-C(2)-C(3)-C(24)	-70.3(6)
C(1)-C(2)-C(3)-C(24)	159.5(4)
C(16)-C(2)-C(3)-C(22)	53.4(6)
C(1)-C(2)-C(3)-C(22)	-76.8(5)
C(16)-C(2)-C(3)-C(4)	170.7(5)
C(1)-C(2)-C(3)-C(4)	40.5(5)
C(24)-C(3)-C(4)-C(5)	-159.0(5)
C(22)-C(3)-C(4)-C(5)	80.5(5)
C(2)-C(3)-C(4)-C(5)	-40.6(5)
C(3)-C(4)-C(5)-C(6)	153.3(6)
C(3)-C(4)-C(5)-C(1)	25.6(6)
C(8)-C(1)-C(5)-C(6)	-6.5(8)
C(2)-C(1)-C(5)-C(6)	-125.7(6)
C(8)-C(1)-C(5)-C(4)	119.6(5)
C(2)-C(1)-C(5)-C(4)	0.4(5)
C(4)-C(5)-C(6)-C(7)	128.2(12)
C(1)-C(5)-C(6)-C(7)	-109.2(13)
C(9)-N(1)-C(8)-O(5)	-5.9(8)
C(9)-N(1)-C(8)-C(1)	171.7(5)
C(2)-C(1)-C(8)-O(5)	52.1(6)
C(5)-C(1)-C(8)-O(5)	-65.3(6)
C(2)-C(1)-C(8)-N(1)	-125.5(5)
C(5)-C(1)-C(8)-N(1)	117.1(5)
C(8)-N(1)-C(9)-C(10)	-97.1(6)
N(1)-C(9)-C(10)-C(15)	-136.1(5)
N(1)-C(9)-C(10)-C(11)	42.4(7)
C(15)-C(10)-C(11)-C(12)	-0.3(10)
C(9)-C(10)-C(11)-C(12)	-178.8(6)
C(10)-C(11)-C(12)-C(13)	0.7(12)
C(11)-C(12)-C(13)-C(14)	-0.9(12)
C(11)-C(12)-C(13)-Br(1)	-179.3(6)
C(12)-C(13)-C(14)-C(15)	0.6(11)
Br(1)-C(13)-C(14)-C(15)	178.9(5)
C(11)-C(10)-C(15)-C(14)	-0.1(9)
C(9)-C(10)-C(15)-C(14)	178.5(6)
C(13)-C(14)-C(15)-C(10)	-0.1(10)
C(1)-C(2)-C(16)-C(21)	37.9(7)

C(3)-C(2)-C(16)-C(21)	-88.0(6)
C(1)-C(2)-C(16)-C(17)	-137.7(5)
C(3)-C(2)-C(16)-C(17)	96.4(6)
C(21)-C(16)-C(17)-C(18)	2.0(9)
C(2)-C(16)-C(17)-C(18)	177.9(6)
C(21)-C(16)-C(17)-Cl(1)	-178.4(5)
C(2)-C(16)-C(17)-Cl(1)	-2.5(7)
C(16)-C(17)-C(18)-C(19)	-3.5(11)
Cl(1)-C(17)-C(18)-C(19)	176.8(6)
C(17)-C(18)-C(19)-C(20)	3.2(12)
C(18)-C(19)-C(20)-C(21)	-1.4(12)
C(17)-C(16)-C(21)-C(20)	-0.2(9)
C(2)-C(16)-C(21)-C(20)	-176.0(6)
C(19)-C(20)-C(21)-C(16)	-0.1(12)
C(23)-O(4)-C(22)-O(3)	-2.2(11)
C(23)-O(4)-C(22)-C(3)	179.8(7)
C(24)-C(3)-C(22)-O(3)	137.1(6)
C(4)-C(3)-C(22)-O(3)	-100.1(7)
C(2)-C(3)-C(22)-O(3)	11.9(8)
C(24)-C(3)-C(22)-O(4)	-44.8(6)
C(4)-C(3)-C(22)-O(4)	77.9(6)
C(2)-C(3)-C(22)-O(4)	-170.1(5)
C(25)-O(2)-C(24)-O(1)	-5.5(12)
C(25)-O(2)-C(24)-C(3)	178.7(9)
C(22)-C(3)-C(24)-O(1)	134.1(6)
C(4)-C(3)-C(24)-O(1)	13.0(8)
C(2)-C(3)-C(24)-O(1)	-98.7(7)
C(22)-C(3)-C(24)-O(2)	-50.1(6)
C(4)-C(3)-C(24)-O(2)	-171.2(5)
C(2)-C(3)-C(24)-O(2)	77.1(6)
C(33)-C(26)-C(27)-C(41)	76.0(5)
C(30)-C(26)-C(27)-C(41)	-158.8(4)
C(33)-C(26)-C(27)-C(28)	-154.5(4)
C(30)-C(26)-C(27)-C(28)	-29.3(6)
C(41)-C(27)-C(28)-C(47)	47.6(7)
C(26)-C(27)-C(28)-C(47)	-78.8(6)
C(41)-C(27)-C(28)-C(49)	-79.4(7)
C(26)-C(27)-C(28)-C(49)	154.2(6)
C(41)-C(27)-C(28)-C(29)	166.6(5)
C(26)-C(27)-C(28)-C(29)	40.2(6)
C(47)-C(28)-C(29)-C(30)	82.2(7)
C(49)-C(28)-C(29)-C(30)	-155.2(7)
C(27)-C(28)-C(29)-C(30)	-37.2(7)
C(28)-C(29)-C(30)-C(31)	147.0(7)

C(28)-C(29)-C(30)-C(26)	19.6(7)
C(33)-C(26)-C(30)-C(31)	2.3(8)
C(27)-C(26)-C(30)-C(31)	-118.8(7)
C(33)-C(26)-C(30)-C(29)	127.3(5)
C(27)-C(26)-C(30)-C(29)	6.2(6)
C(29)-C(30)-C(31)-C(32)	118.2(17)
C(26)-C(30)-C(31)-C(32)	-120.6(16)
C(34)-N(2)-C(33)-O(10)	-5.1(7)
C(34)-N(2)-C(33)-C(26)	172.6(4)
C(27)-C(26)-C(33)-O(10)	47.2(6)
C(30)-C(26)-C(33)-O(10)	-72.3(6)
C(27)-C(26)-C(33)-N(2)	-130.5(4)
C(30)-C(26)-C(33)-N(2)	110.0(5)
C(33)-N(2)-C(34)-C(35)	-83.4(6)
N(2)-C(34)-C(35)-C(40)	177.9(11)
N(2)-C(34)-C(35)-C(36)	-0.2(9)
C(40)-C(35)-C(36)-C(37)	2.9(16)
C(34)-C(35)-C(36)-C(37)	-179.0(9)
C(35)-C(36)-C(37)-C(38)	-3(2)
C(36)-C(37)-C(38)-C(39)	7(3)
C(36)-C(37)-C(38)-Br(2A)	155.9(10)
C(36)-C(37)-C(38)-Br(2)	-156.2(9)
C(37)-C(38)-C(39)-C(40)	-11(4)
Br(2A)-C(38)-C(39)-C(40)	-162(2)
Br(2)-C(38)-C(39)-C(40)	151.4(18)
C(36)-C(35)-C(40)-C(39)	-7(3)
C(34)-C(35)-C(40)-C(39)	175.1(19)
C(38)-C(39)-C(40)-C(35)	10(4)
C(26)-C(27)-C(41)-C(42)	-128.4(5)
C(28)-C(27)-C(41)-C(42)	109.8(6)
C(26)-C(27)-C(41)-C(46)	48.6(7)
C(28)-C(27)-C(41)-C(46)	-73.1(7)
C(46)-C(41)-C(42)-C(43)	4.7(8)
C(27)-C(41)-C(42)-C(43)	-178.1(5)
C(46)-C(41)-C(42)-Cl(2)	-172.5(5)
C(27)-C(41)-C(42)-Cl(2)	4.6(7)
C(41)-C(42)-C(43)-C(44)	-1.9(10)
Cl(2)-C(42)-C(43)-C(44)	175.4(6)
C(42)-C(43)-C(44)-C(45)	-4.7(13)
C(43)-C(44)-C(45)-C(46)	8.2(16)
C(42)-C(41)-C(46)-C(45)	-1.2(11)
C(27)-C(41)-C(46)-C(45)	-178.4(8)
C(44)-C(45)-C(46)-C(41)	-5.2(15)
C(48)-O(7)-C(47)-O(6)	0(2)

C(48)-O(7)-C(47)-C(28)	176.2(17)
C(49)-C(28)-C(47)-O(6)	-137.0(9)
C(29)-C(28)-C(47)-O(6)	-17.0(11)
C(27)-C(28)-C(47)-O(6)	95.6(10)
C(49)-C(28)-C(47)-O(7)	46.4(9)
C(29)-C(28)-C(47)-O(7)	166.5(7)
C(27)-C(28)-C(47)-O(7)	-81.0(9)
C(50)-O(9)-C(49)-O(8)	-4(2)
C(50)-O(9)-C(49)-C(28)	178.4(15)
C(47)-C(28)-C(49)-O(8)	-140.3(9)
C(29)-C(28)-C(49)-O(8)	97.0(9)
C(27)-C(28)-C(49)-O(8)	-13.0(12)
C(47)-C(28)-C(49)-O(9)	37.4(10)
C(29)-C(28)-C(49)-O(9)	-85.3(10)
C(27)-C(28)-C(49)-O(9)	164.6(8)

Table 10. Hydrogen bonds for 171 [A and deg.].

D-H...A	d(D-H)	d(H...A)	d(D...A)	<(DHA)
N(1)-H(1A)...O(10)	0.86	2.01	2.856(5)	168.2
N(2)-H(2A)...O(5)#1	0.86	2.02	2.860(6)	165.4

Symmetry transformations used to generate equivalent atoms:
#1 x+1,y,z

5. References:

[1] J. Tsuji; I. Shimizu; Y. Ohashi *Tetrahedron Lett.* **1985**, 26, 3825-3828.
[2] N. Ishizuka; K. Matsumura *J. Med. Chem.* **2002**, *45*, 2041-2055.

6. Original ^{1}H and ^{13}C NMR spectra

Dimethyl 3-formyl-2-phenyl-4-vinylcyclopentane-1,1-dicarboxylate (3a)

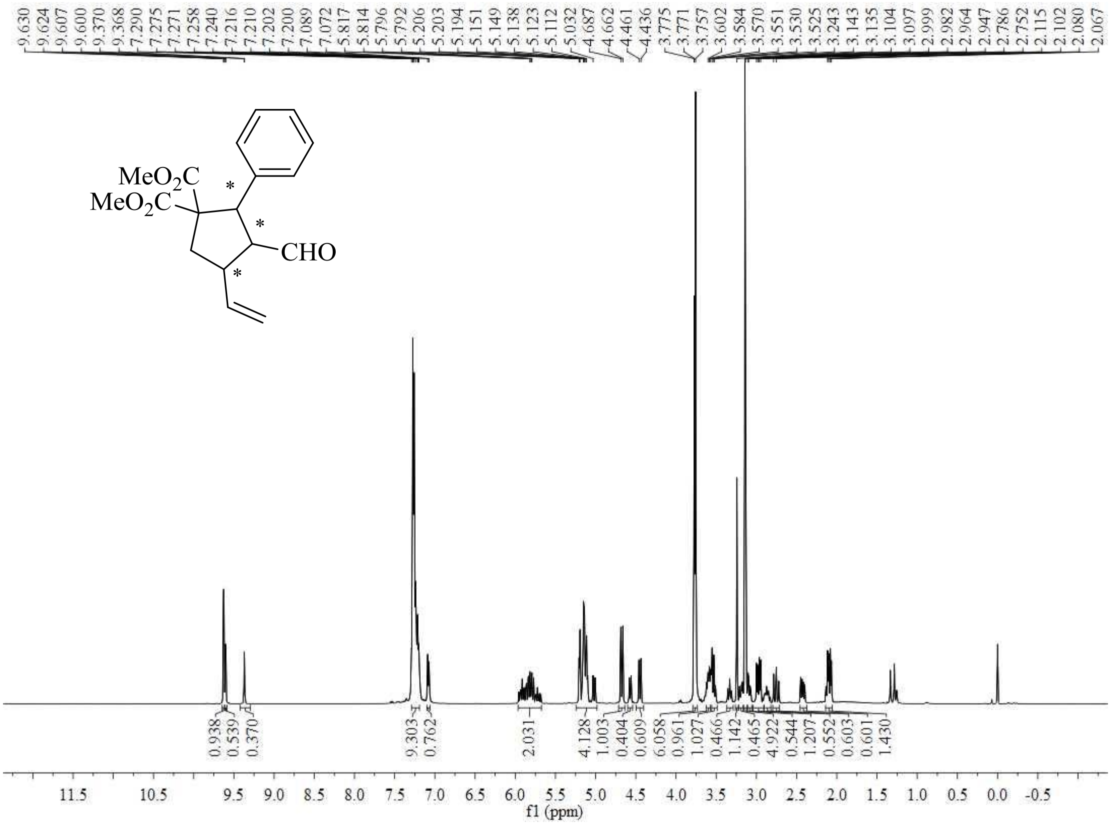

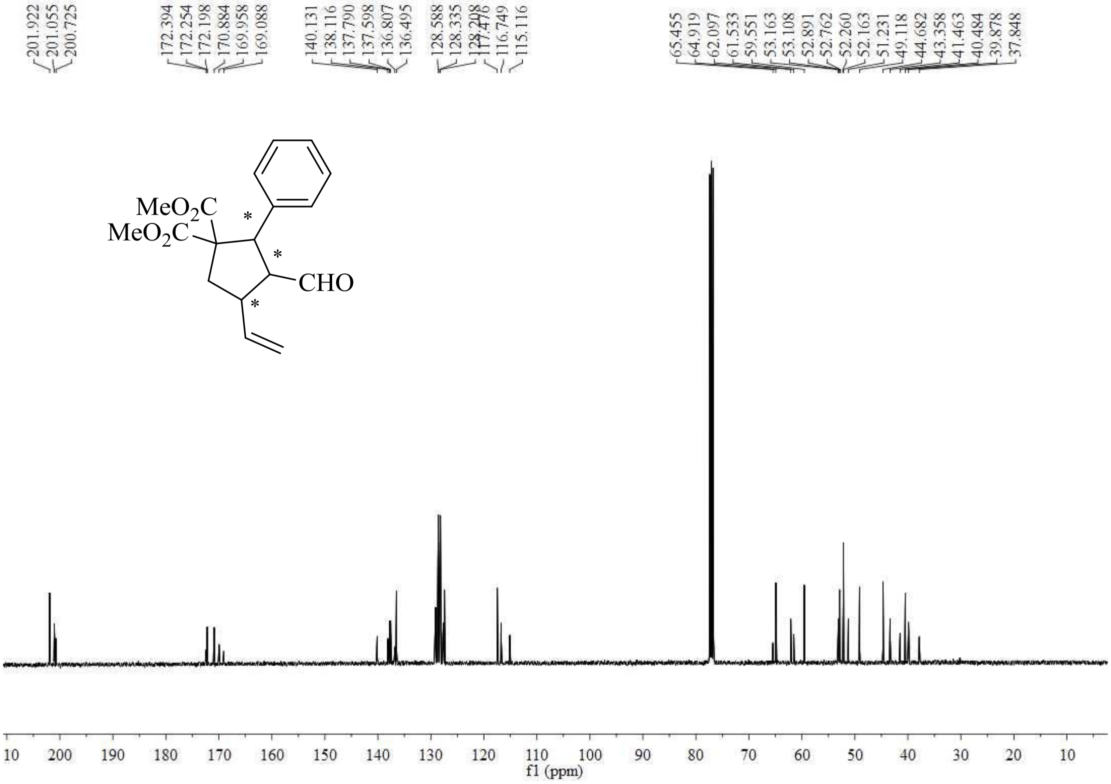

Dimethyl 2-(4-chlorophenyl)-3-formyl-4-vinylcyclopentane-1,1-dicarboxylate (3b)

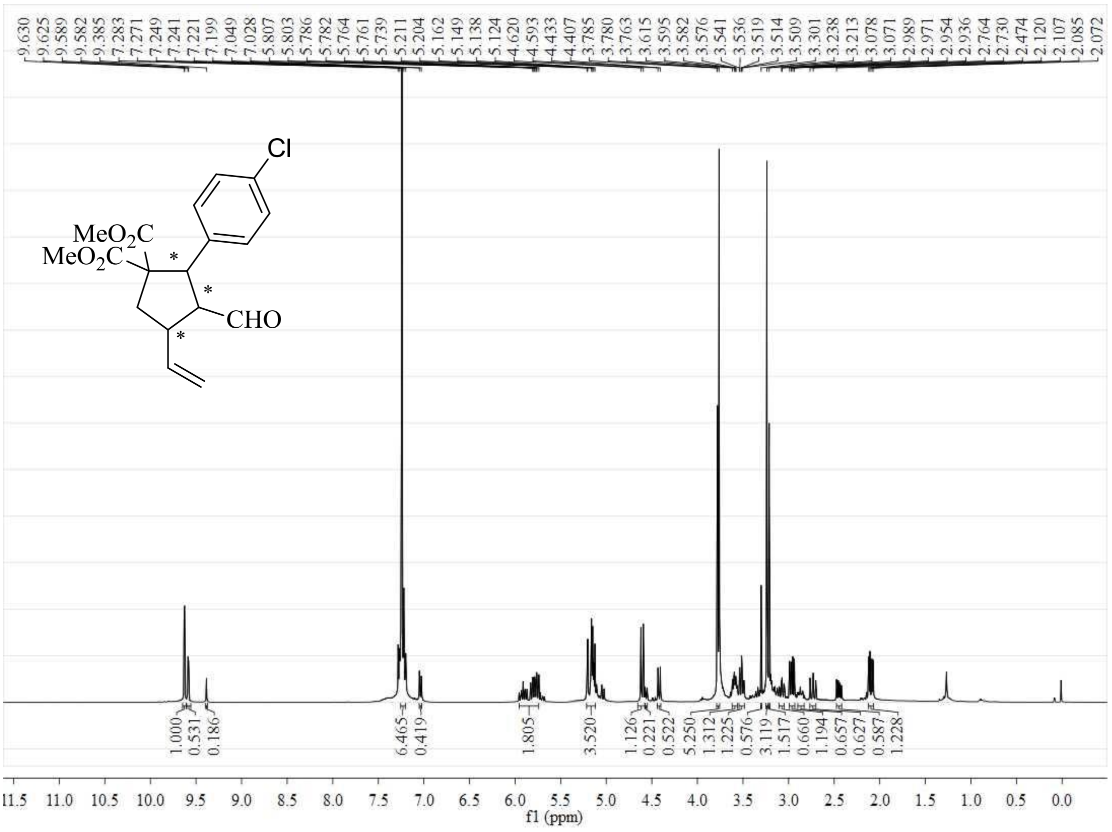

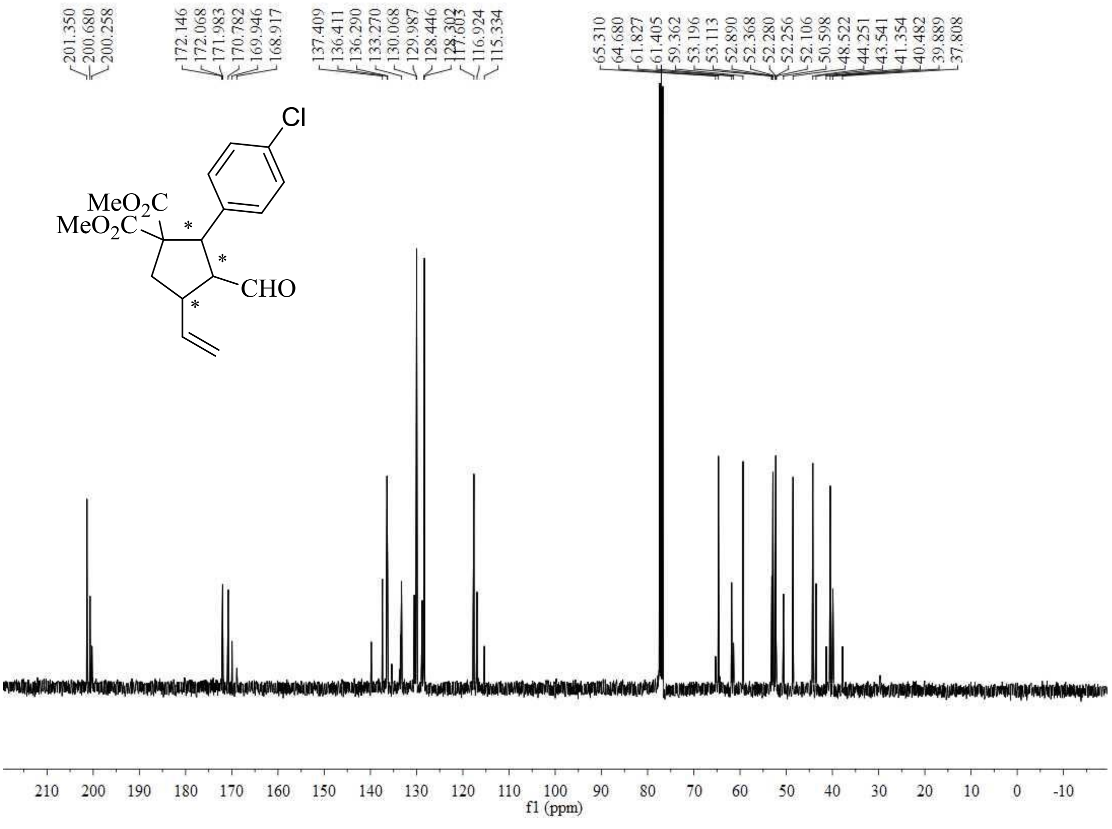

Dimethyl2-(4-bromophenyl)-3-formyl-4-vinylcyclopentane-1,1-dicarboxylate (3c)

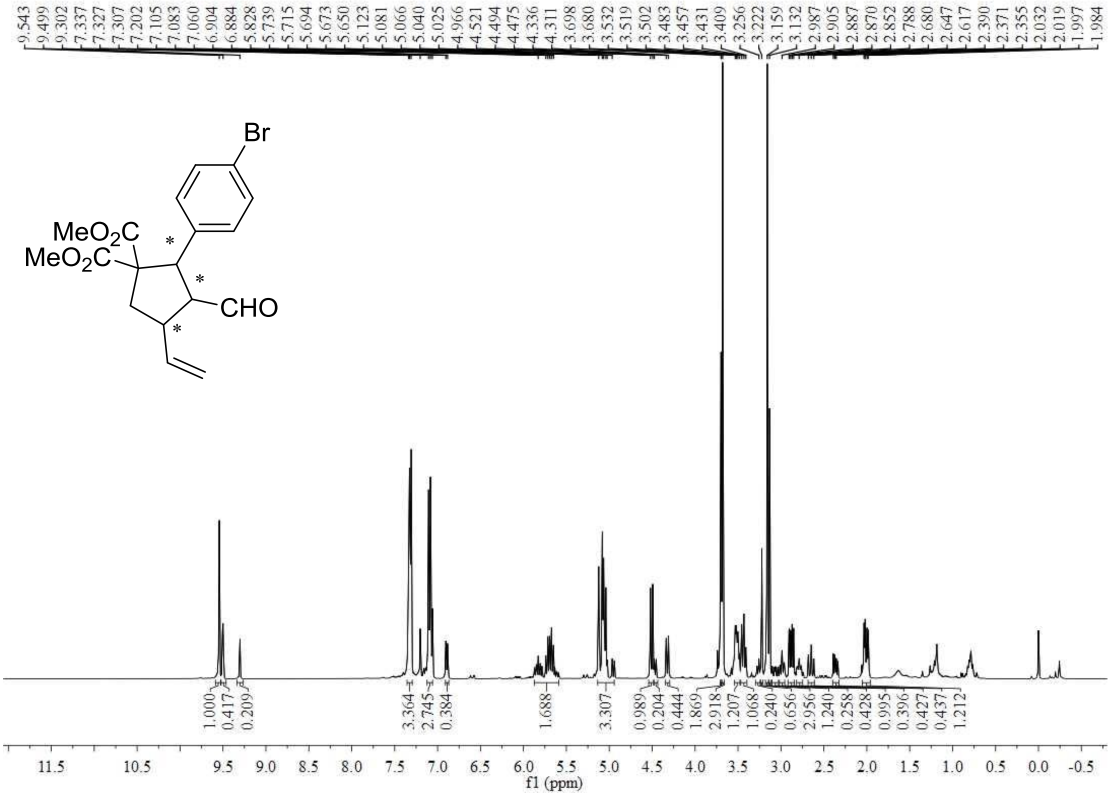

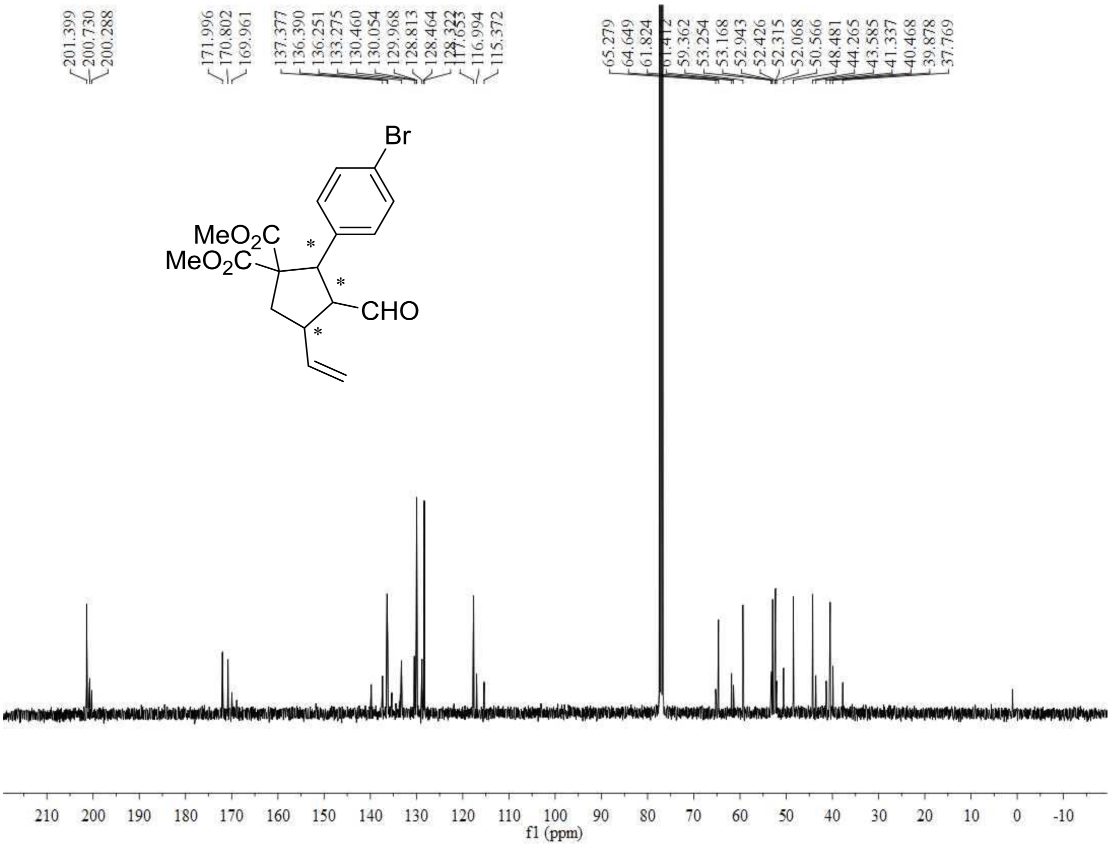

Dimethyl 3-formyl-2-(*p*-tolyl)-4-vinylcyclopentane-1,1-dicarboxylate (3d).

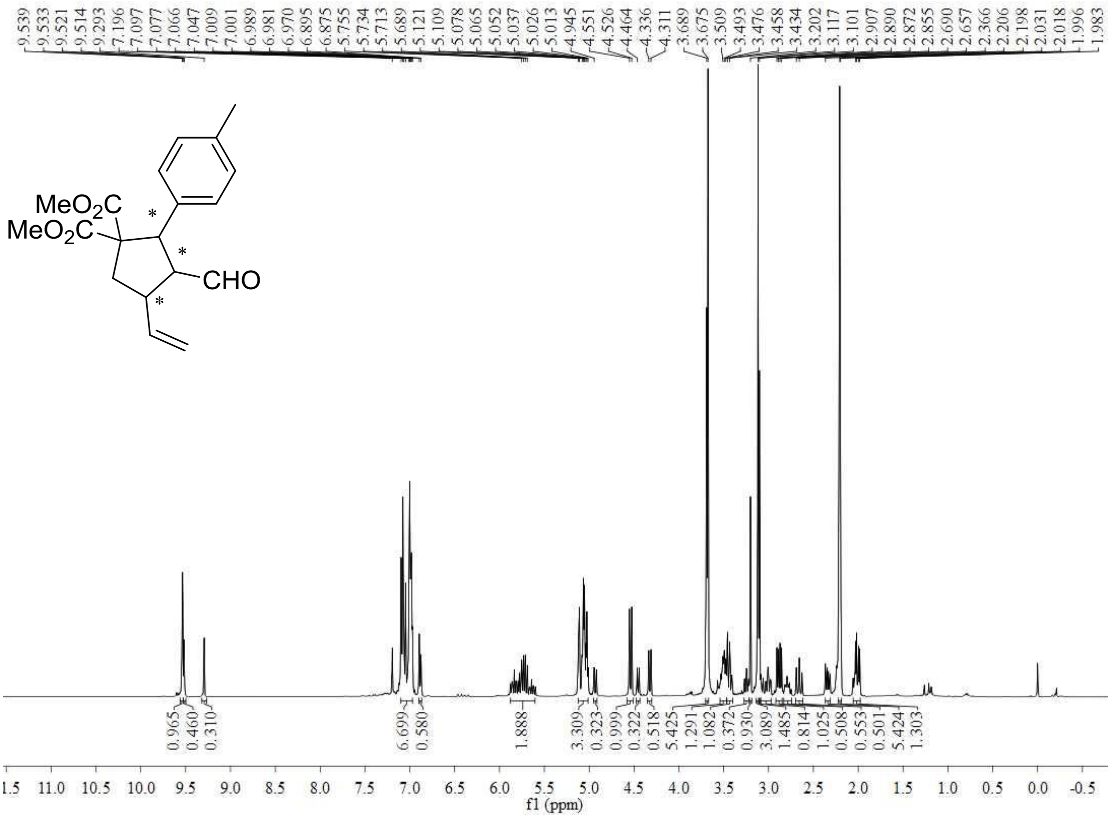

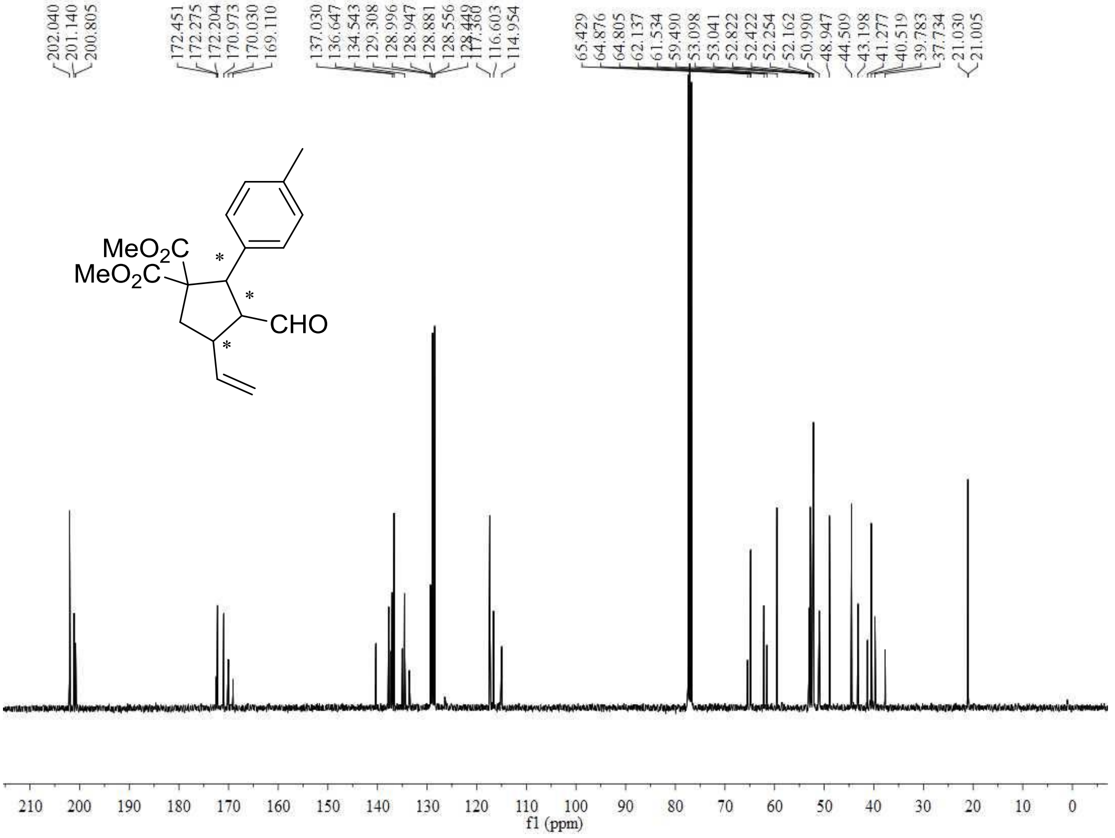

Dimethyl 3-formyl-2-(4-methoxyphenyl)-4-vinylcyclopentane-1,1-dicarboxylate (3e)

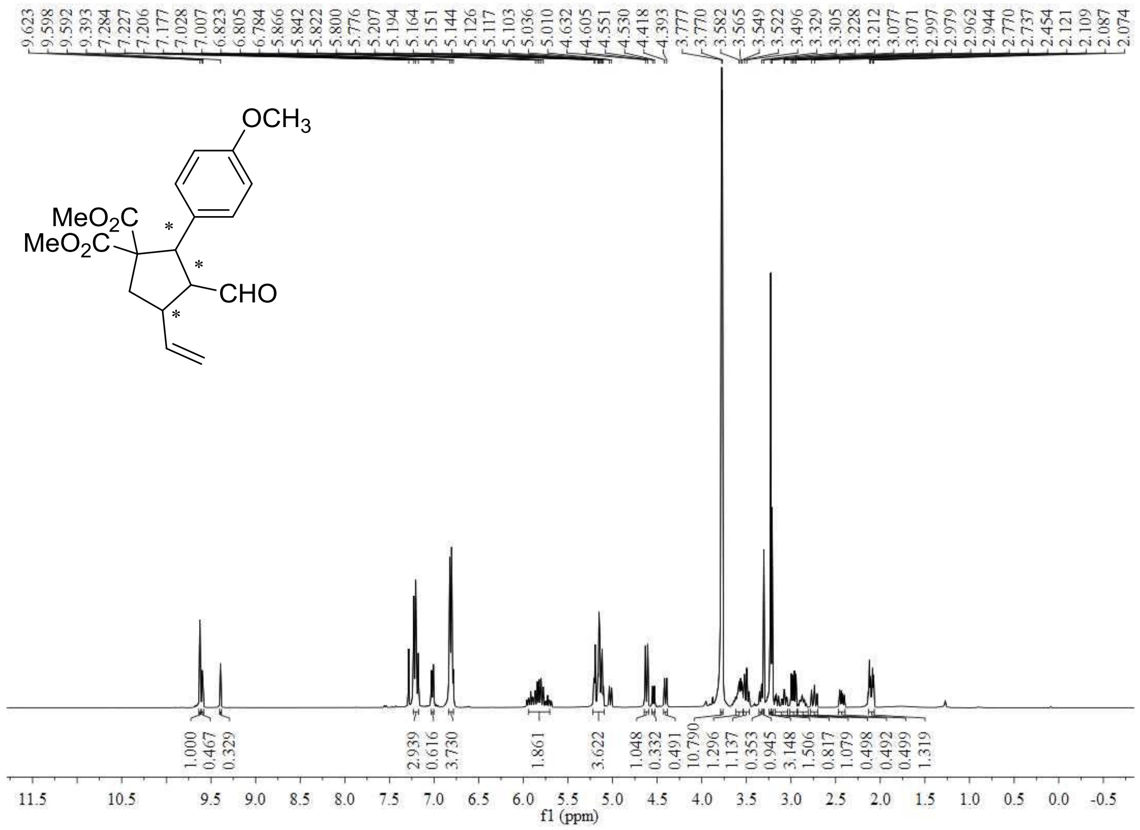

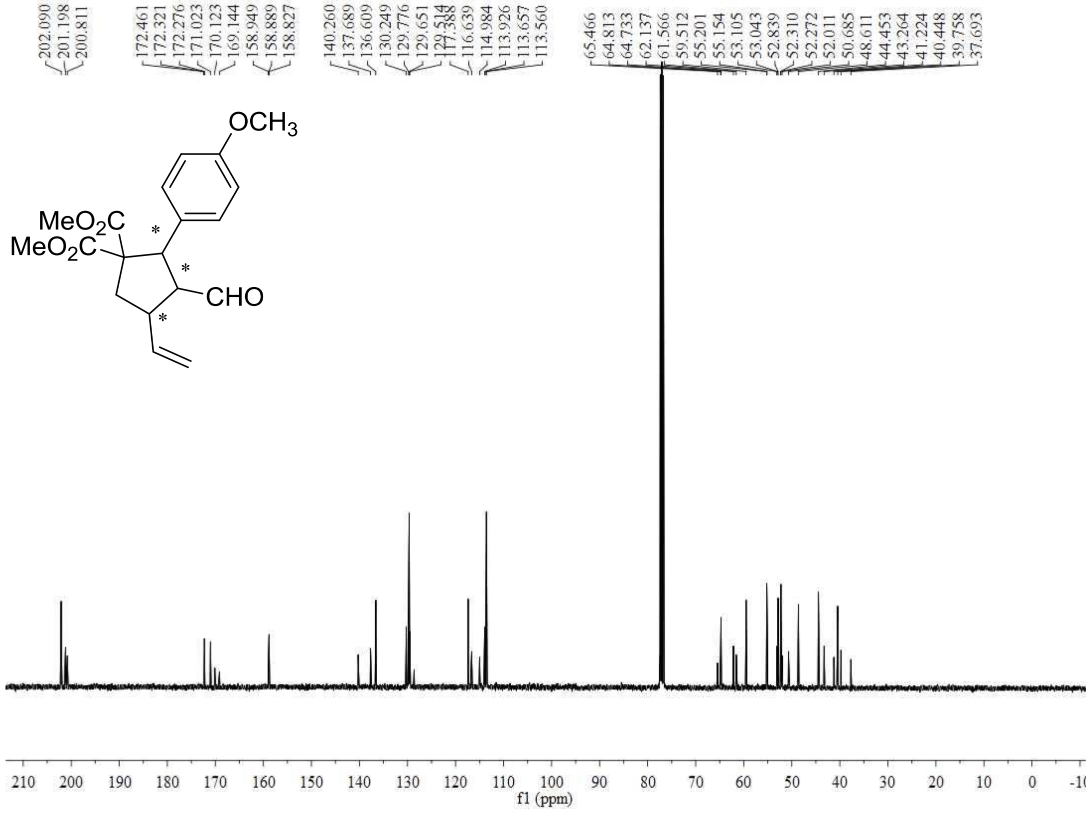

Dimethyl 2-(3-fluorophenyl)-3-formyl-4-vinylcyclopentane-1,1-dicarboxylate (3f)

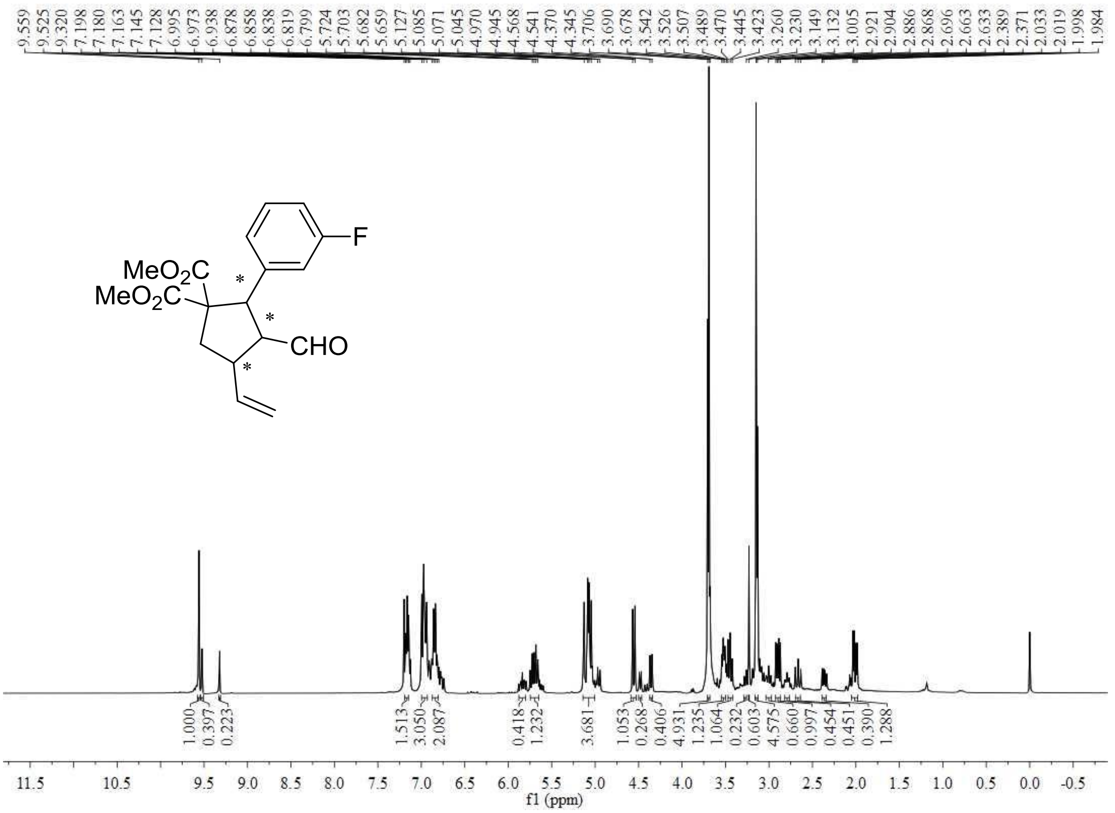

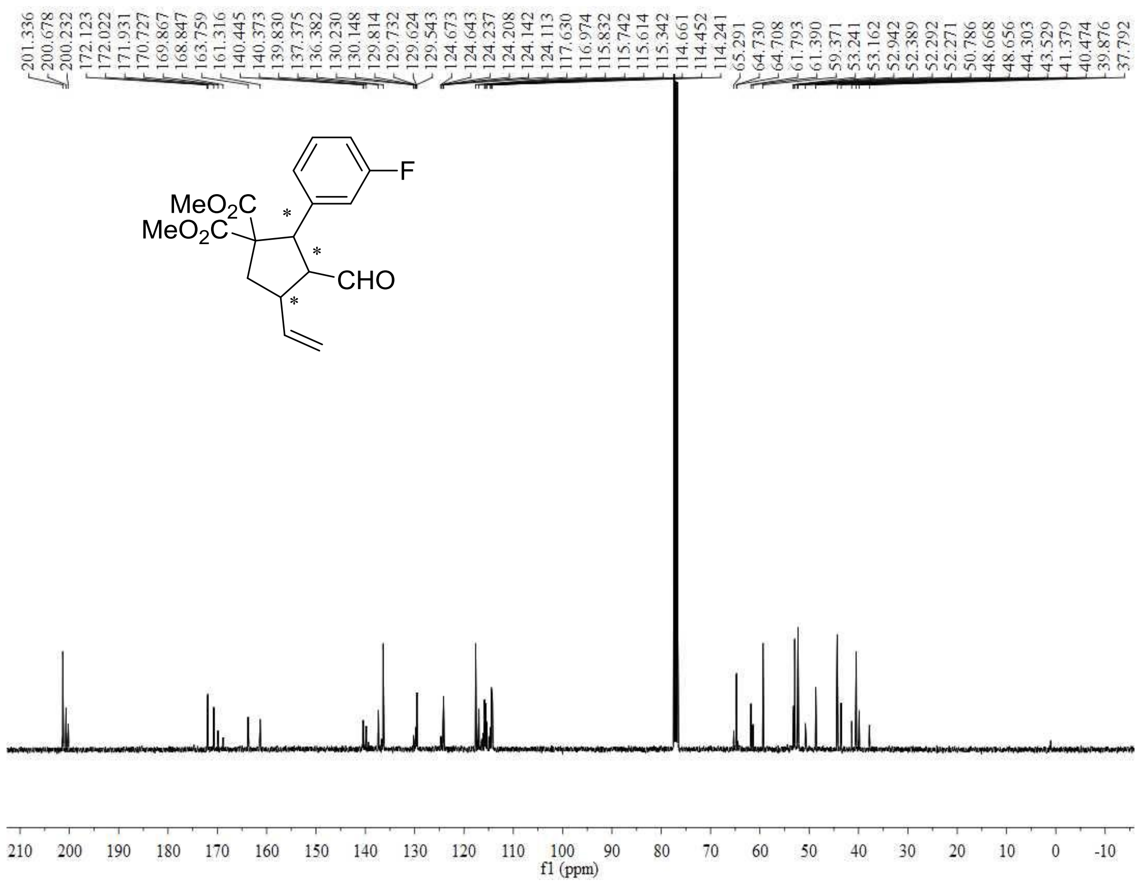

Dimethyl 3-formyl-2-(3-(trifluoromethyl)phenyl)-4-vinylcyclopentane-1,1-dicarboxylate (3g)

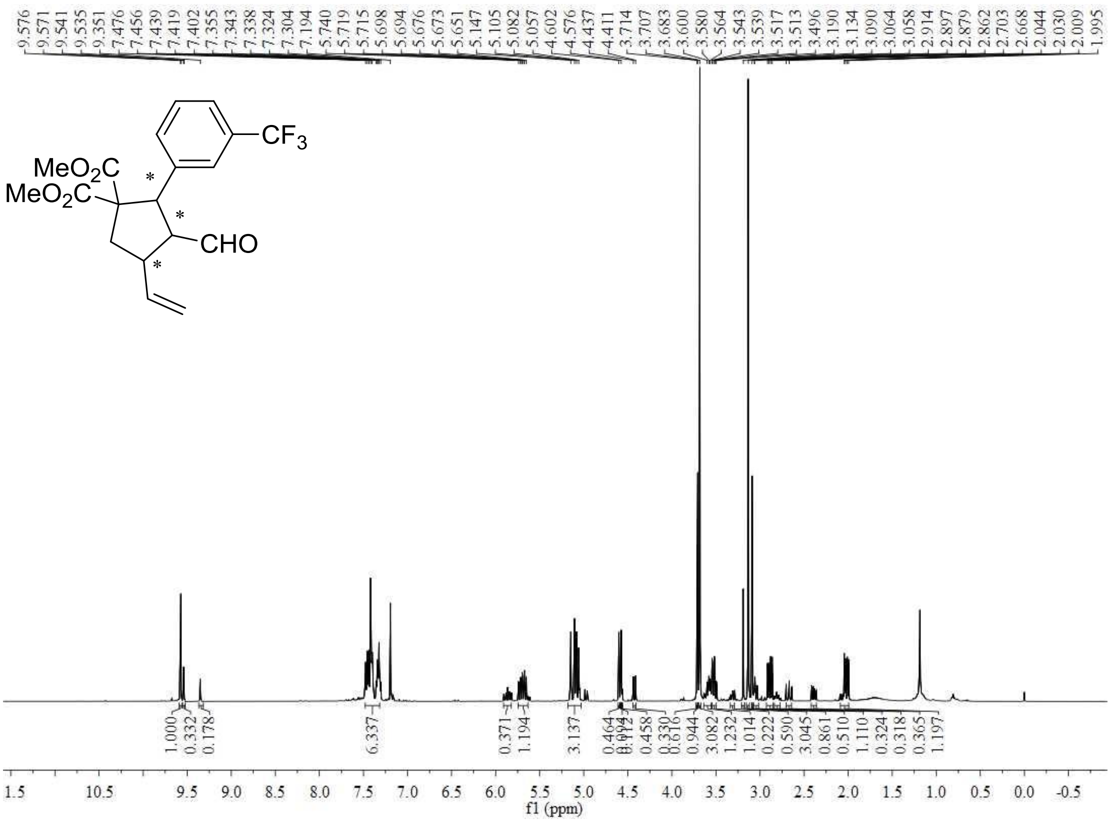

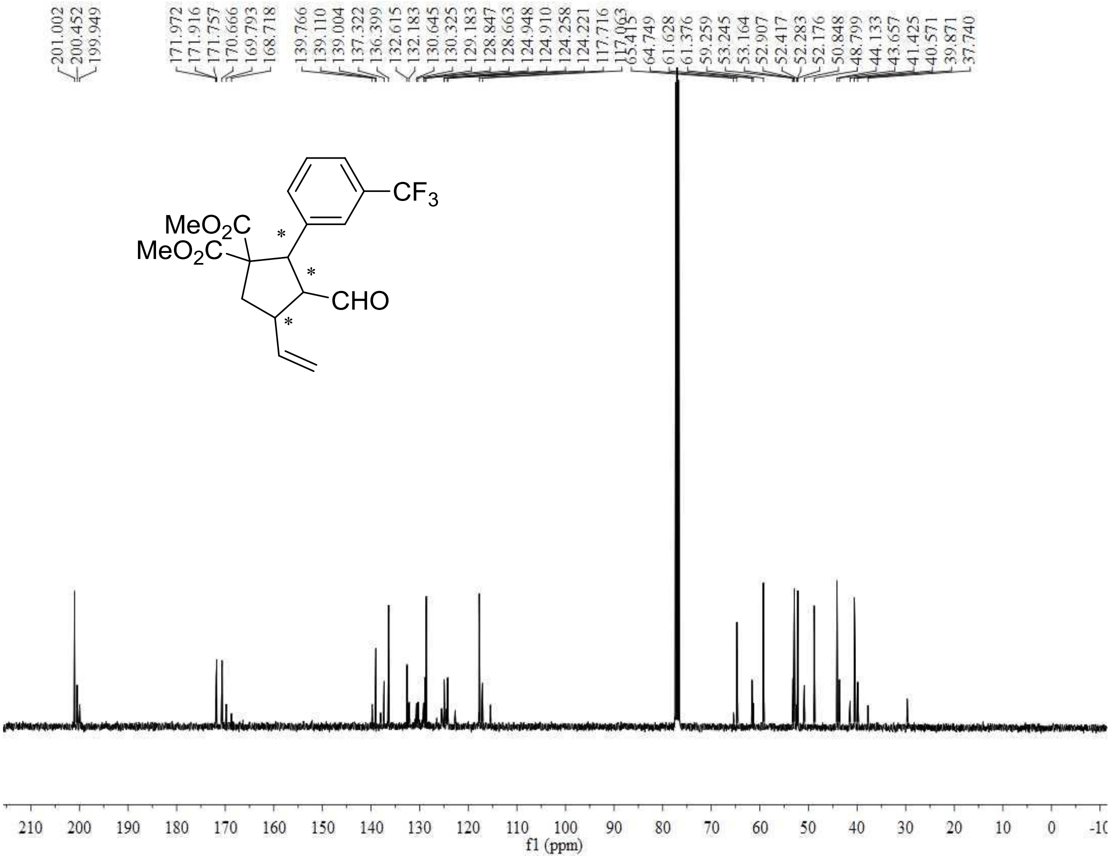

Dimethyl 2-(2-chlorophenyl)-3-formyl-4-vinylcyclopentane-1,1-dicarboxylate (3h)

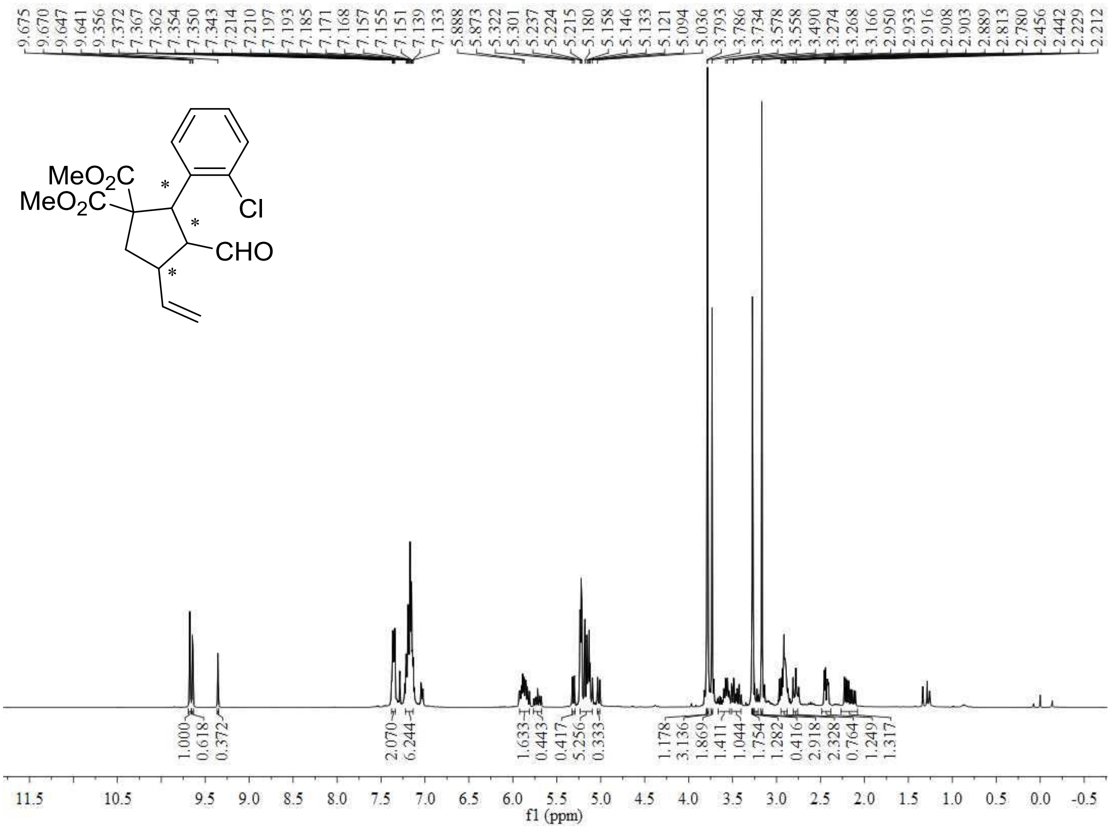

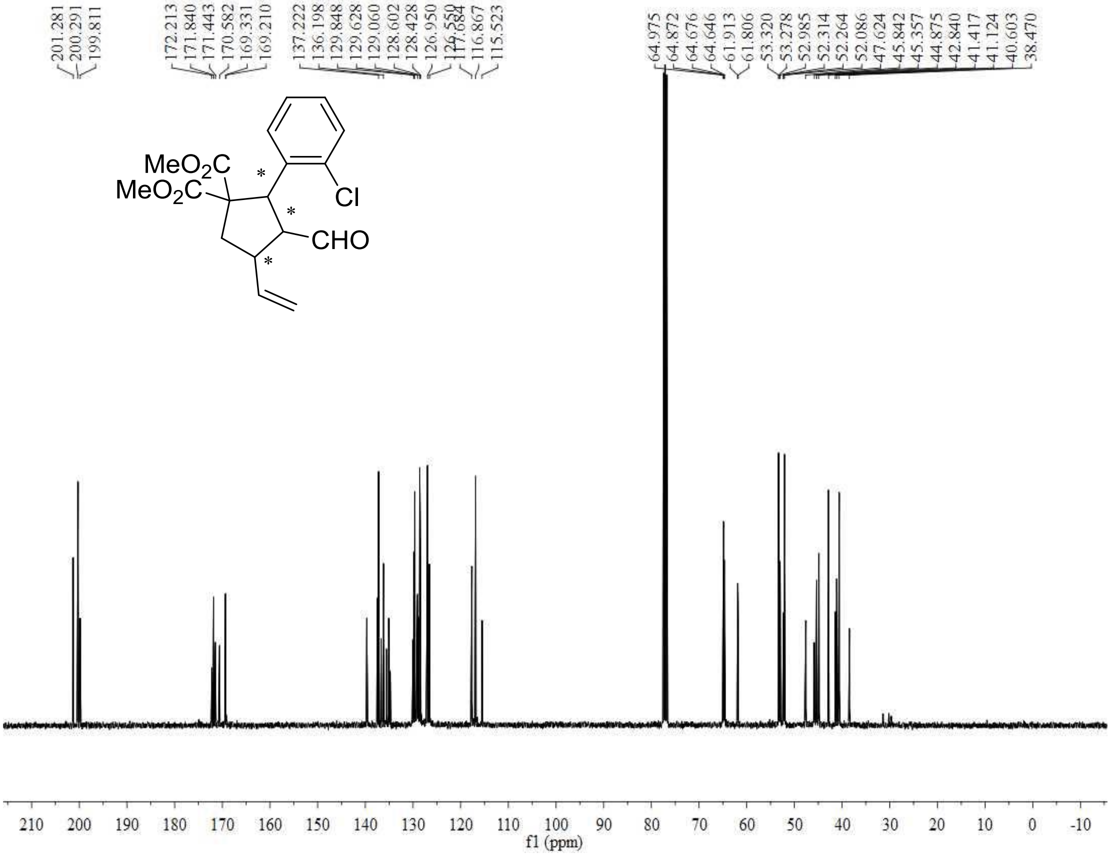

Dimethyl 2-(2-bromophenyl)-3-formyl-4-vinylcyclopentane-1,1-dicarboxylate (3i)

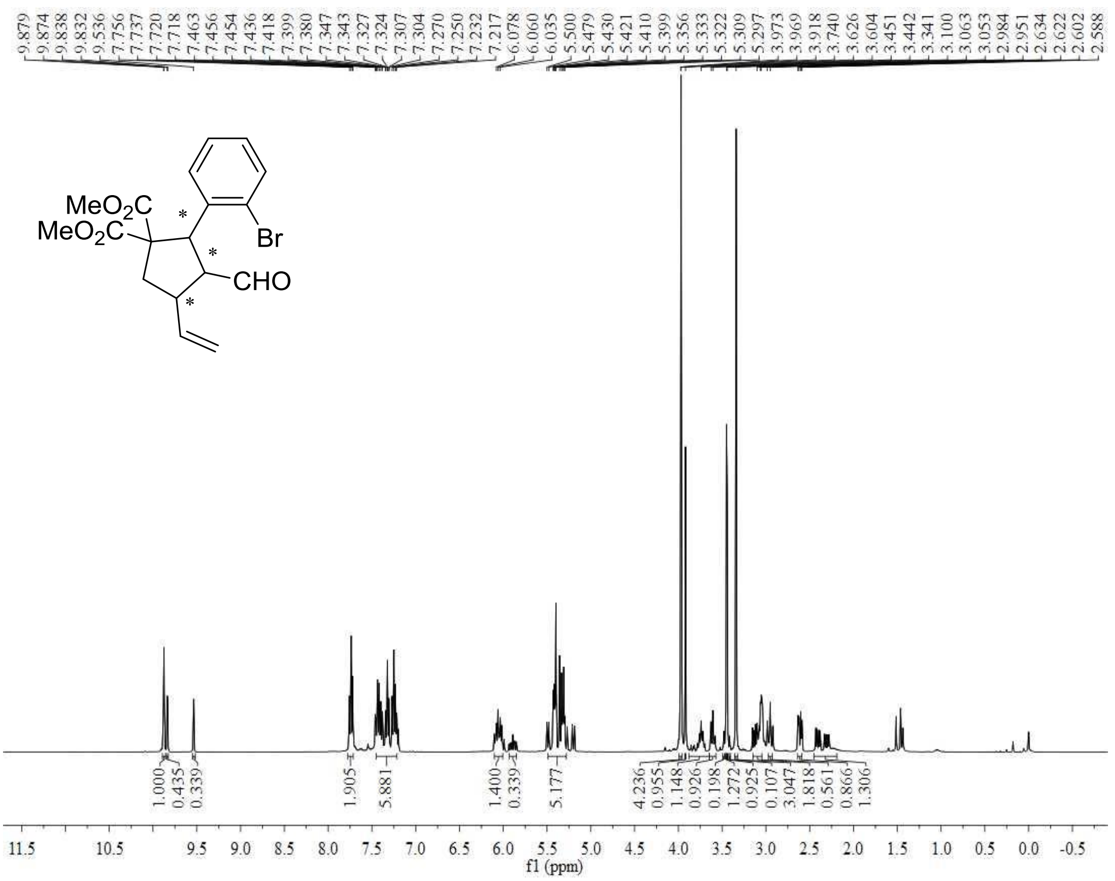

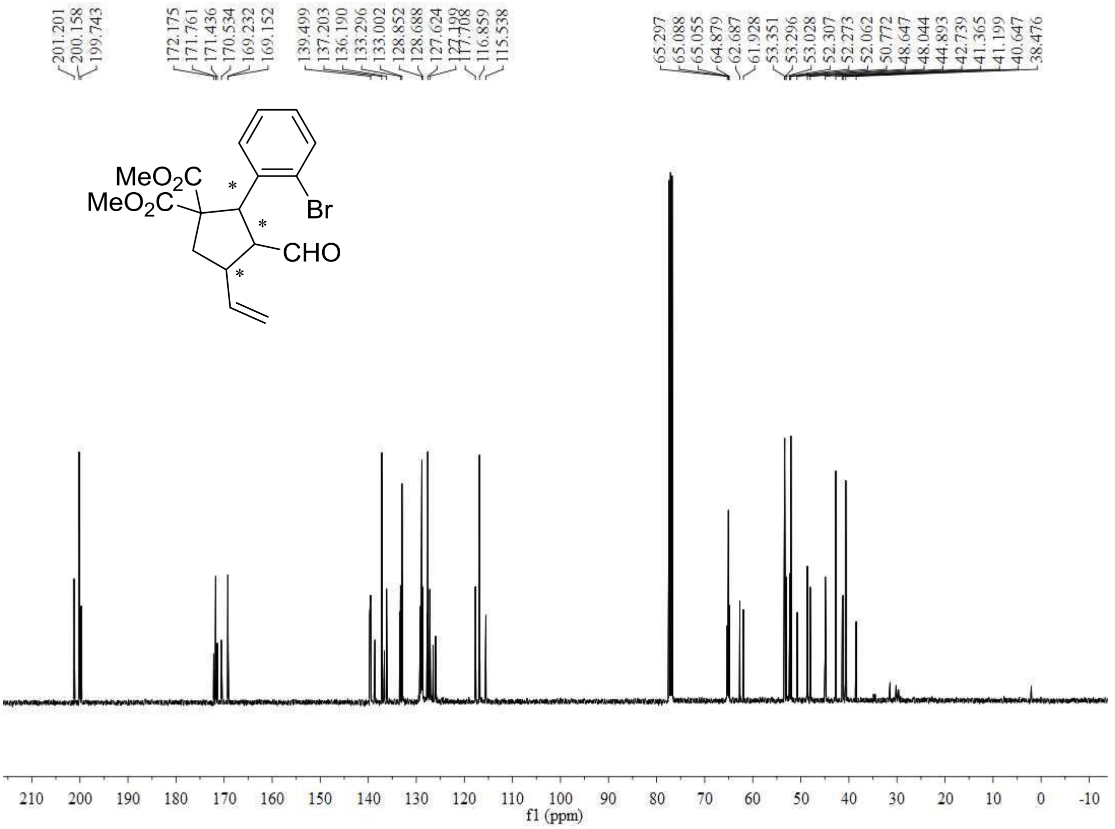

Dimethyl 3-formyl-2-(*o*-tolyl)-4-vinylcyclopentane-1,1-dicarboxylate (3j)

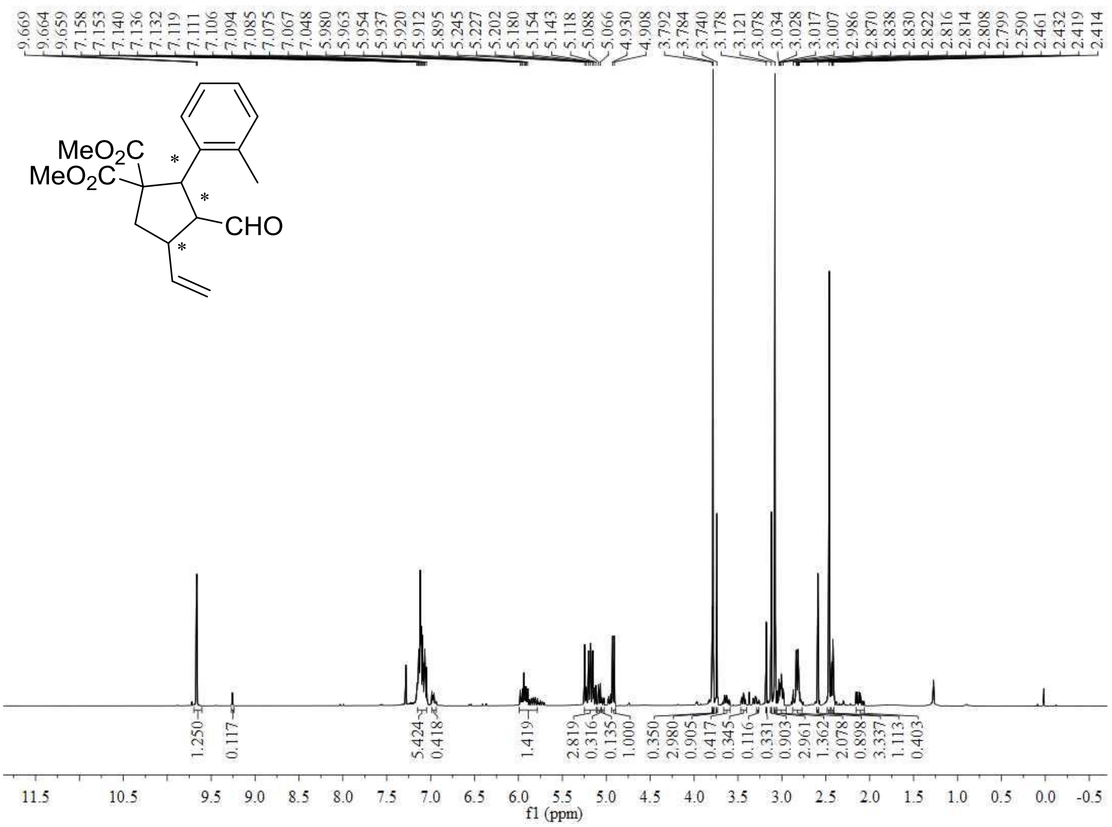

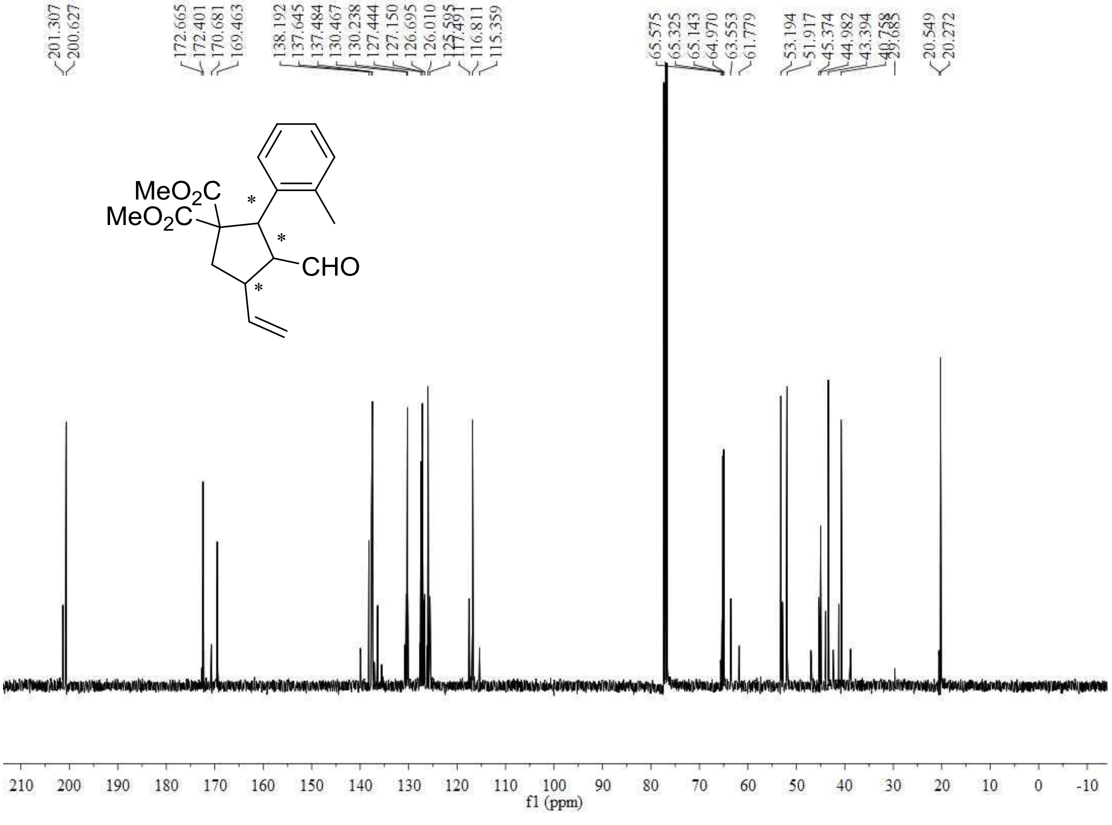

Dimethyl 3-formyl-2-(2-methoxyphenyl)-4-vinylcyclopentane-1,1-dicarboxylate (3k)

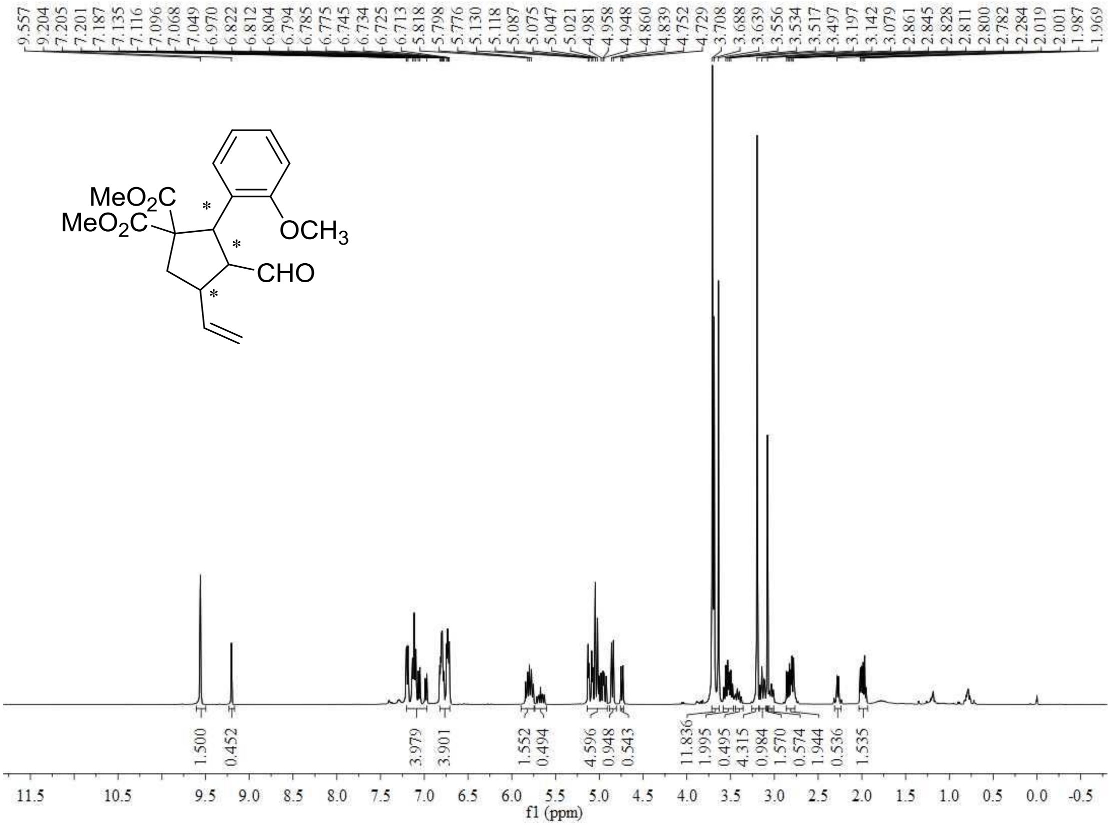

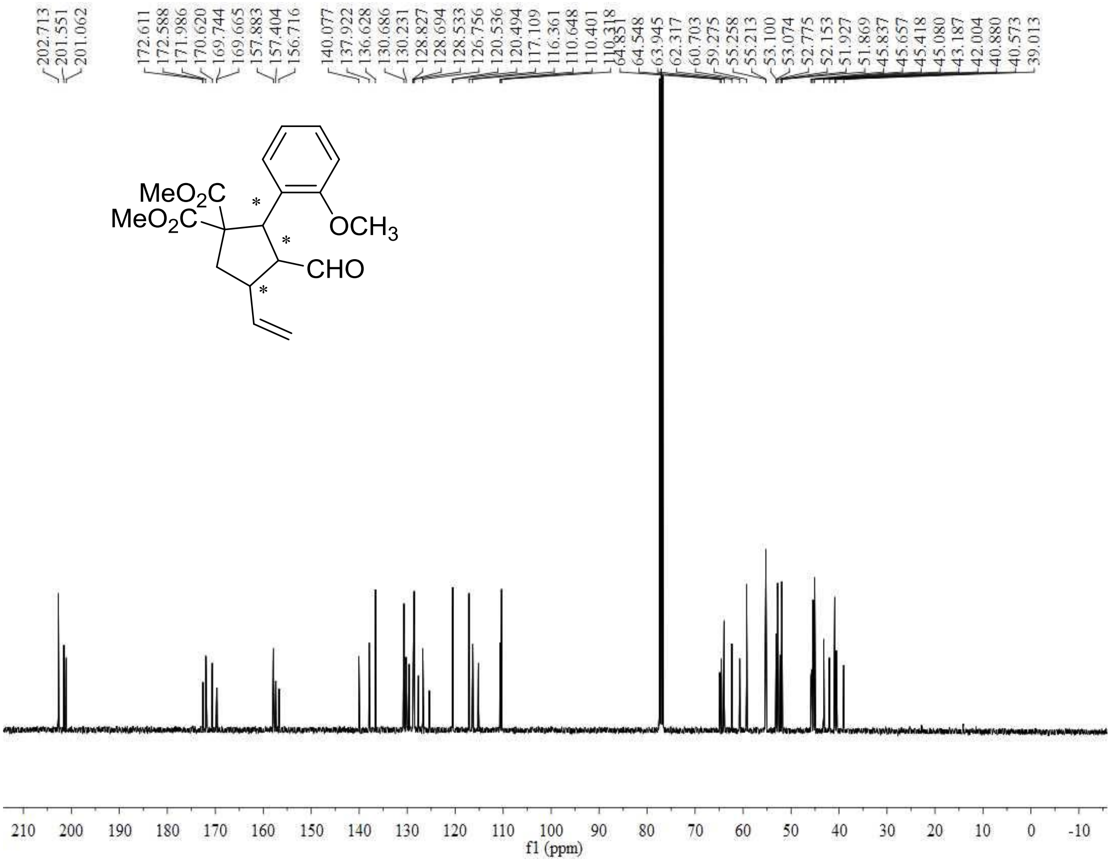

Dimethyl 3-formyl-2-(furan-2-yl)-4-vinylcyclopentane-1,1-dicarboxylate (3l)

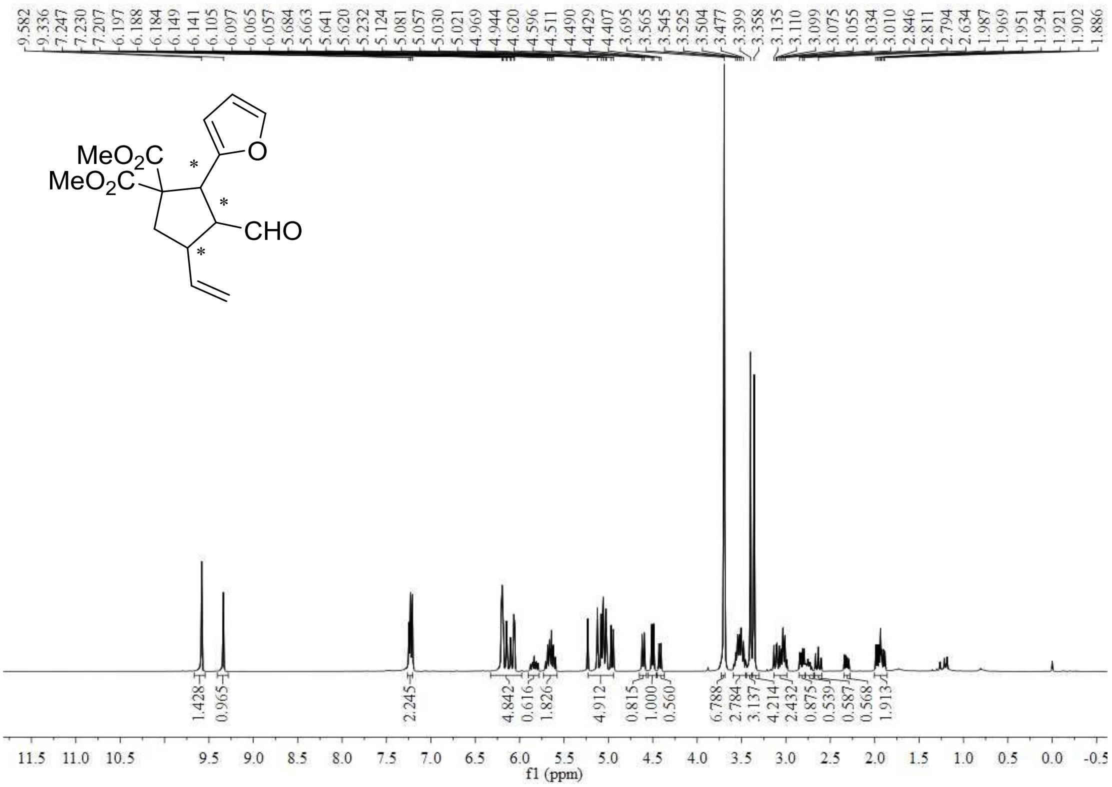

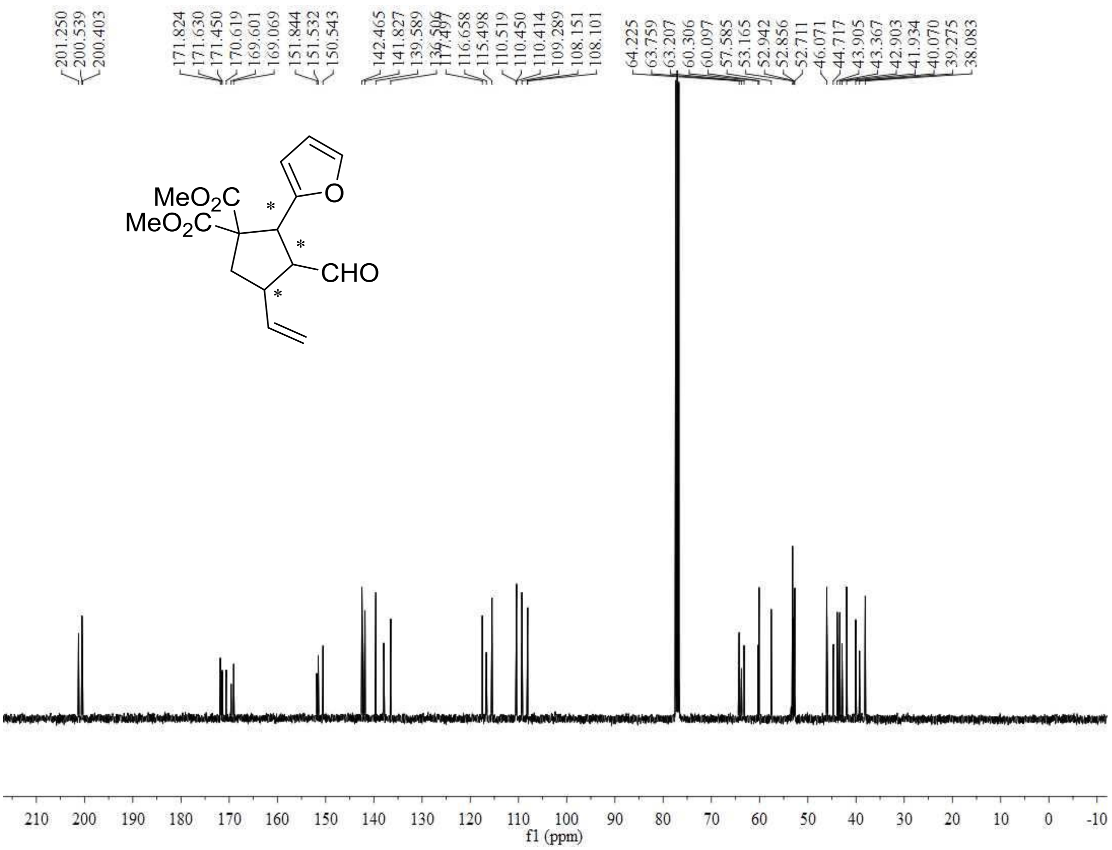

Dimethyl 2-(4-chlorophenyl)-3-formyl-4-((*E*)-styryl)cyclopentane-1,1-dicarbox ylate (3m)

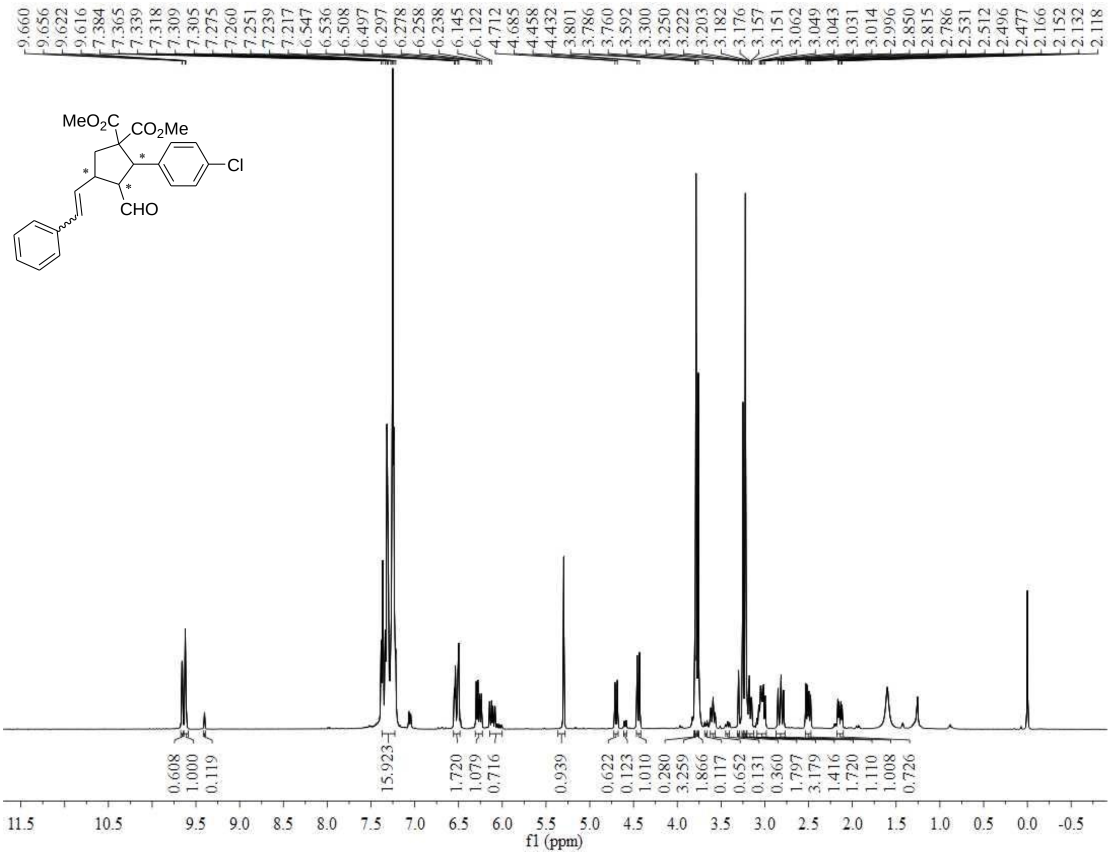

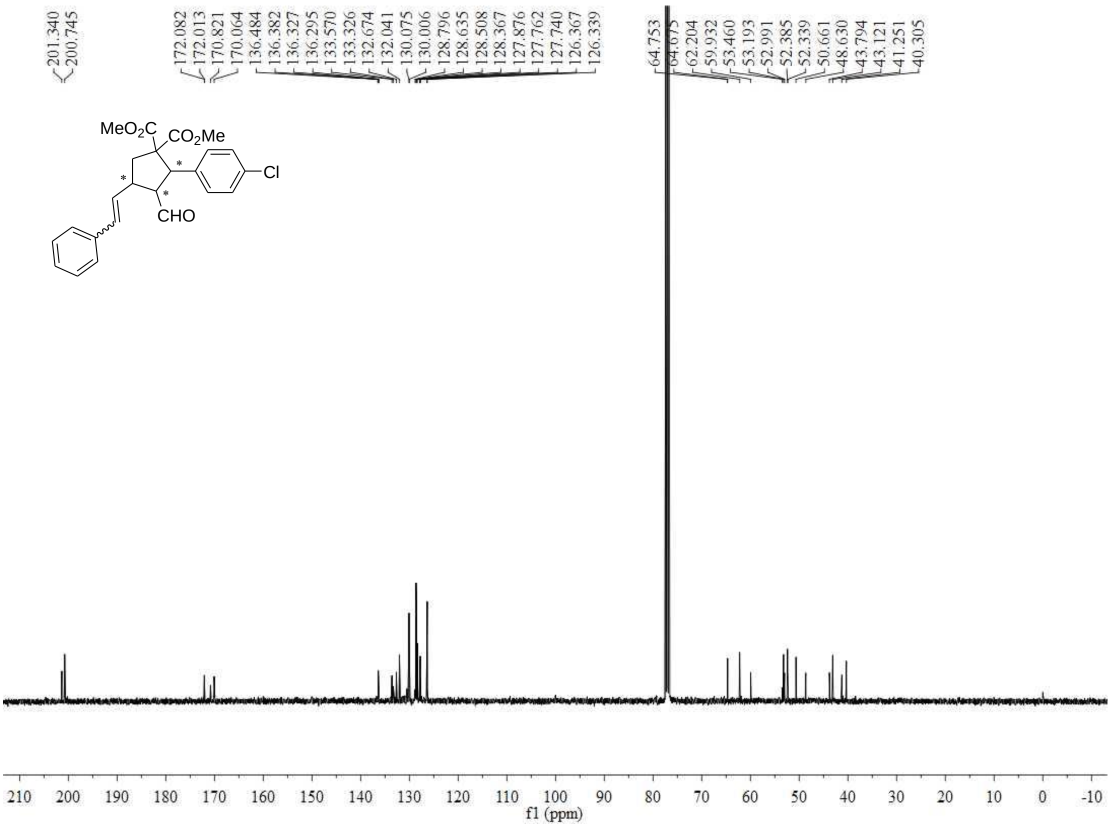

(2*R*,3*S*,4*S*)-Dimethyl 3-((4-bromobenzyl)carbamoyl)-2-(2-chlorophenyl)-4-vinylcyclopentane-1,1-dicarboxylate (7h')

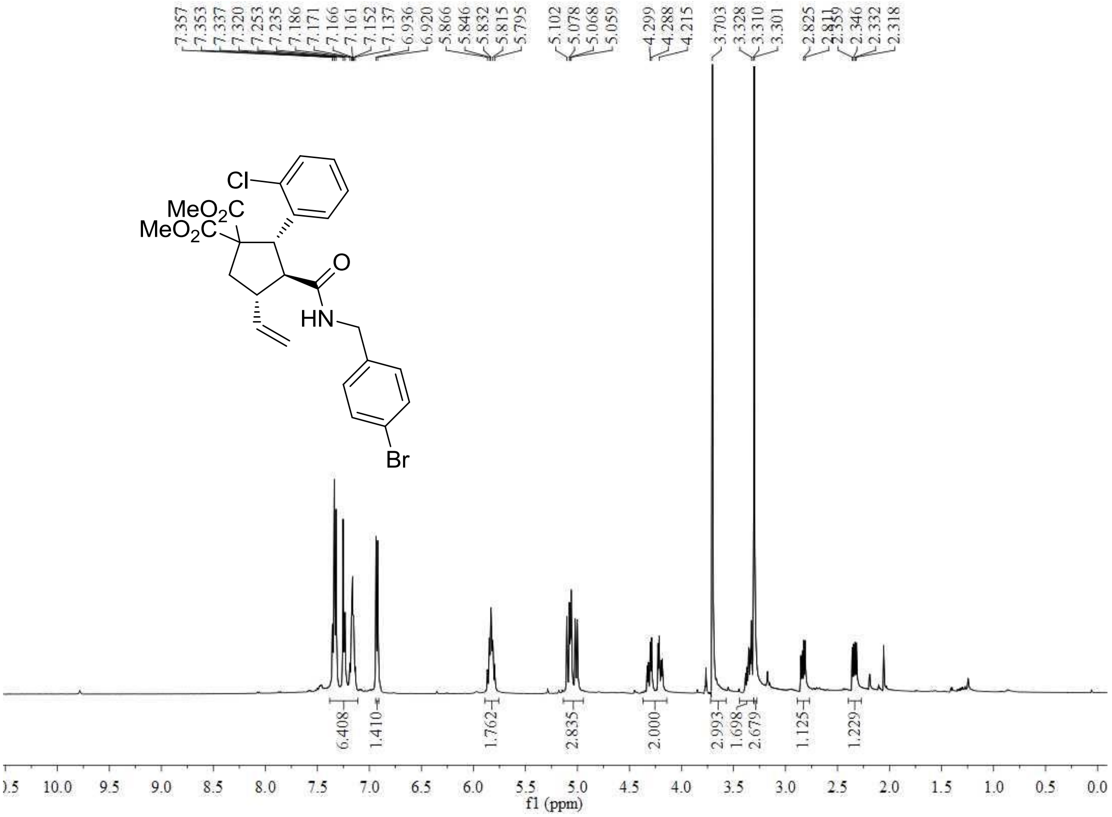

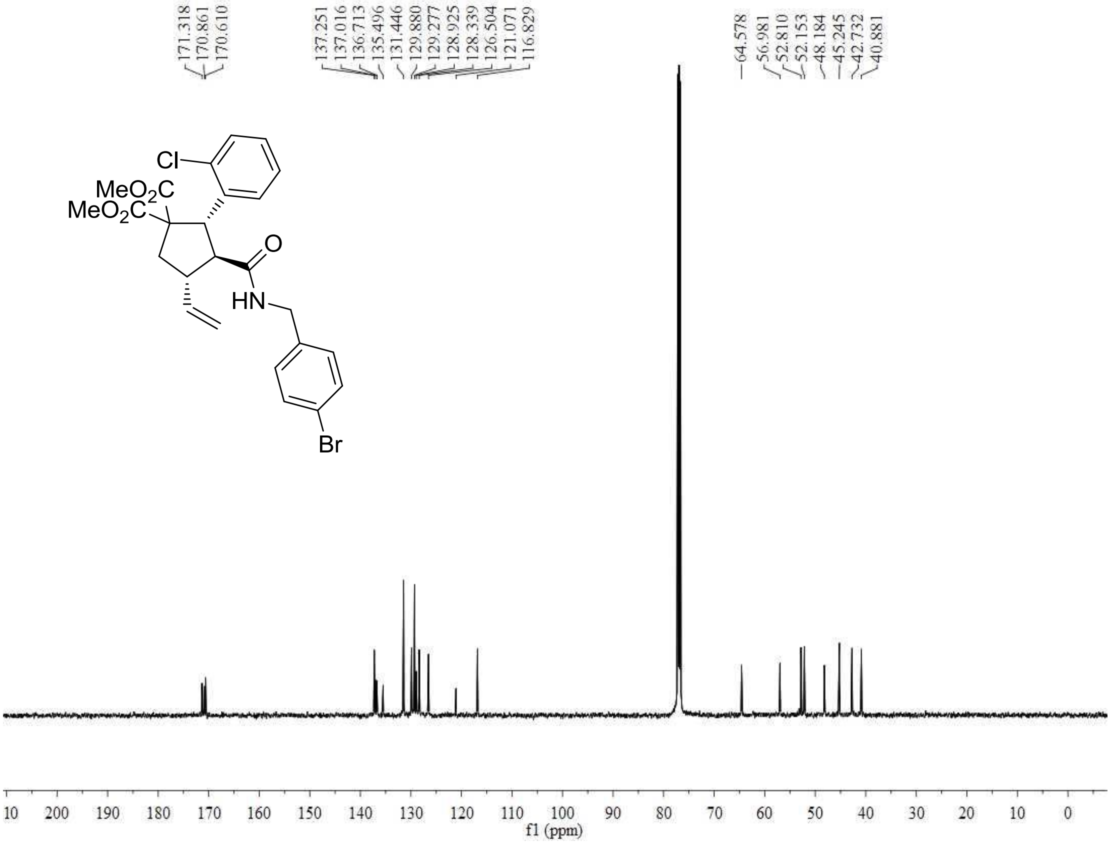

(2*R*,3*S*,4*R*)-Dimethyl 3-((4-bromobenzyl)carbamoyl)-2-(2-chlorophenyl)-4-vinylcyclopentane-1,1-dicarboxylate -dicarboxylate (7h’’)

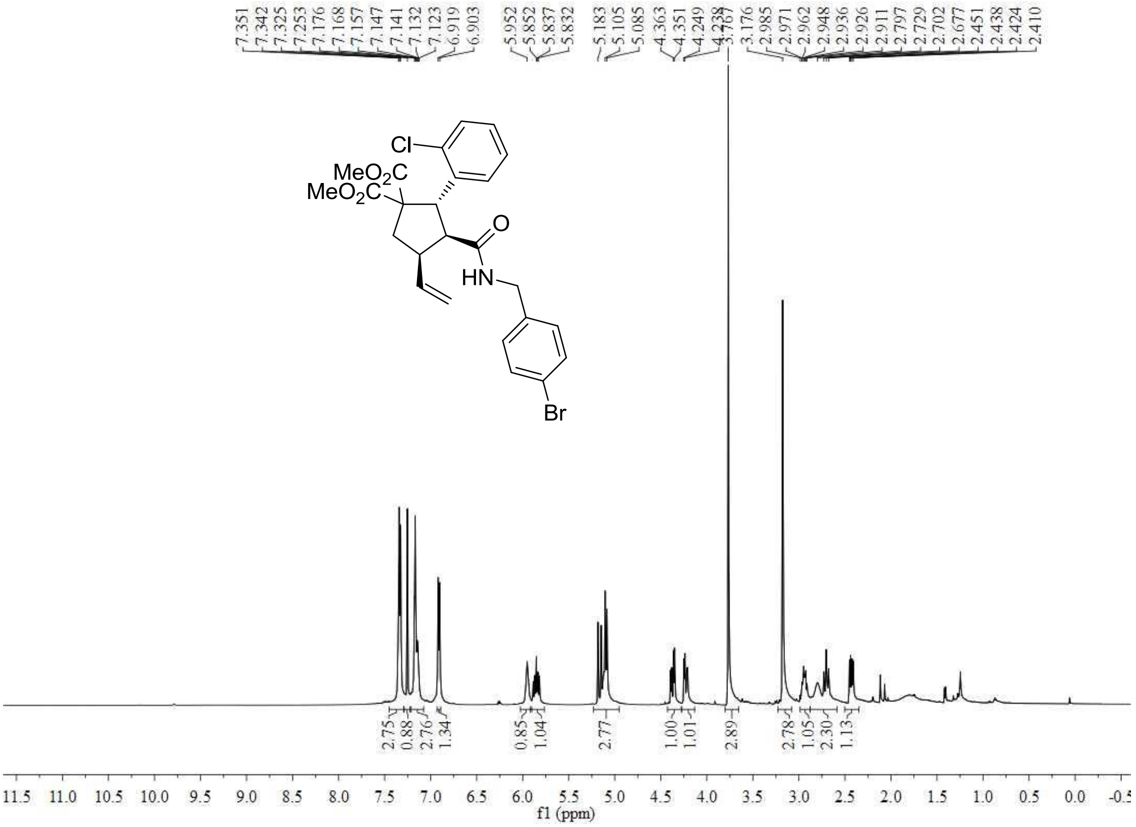

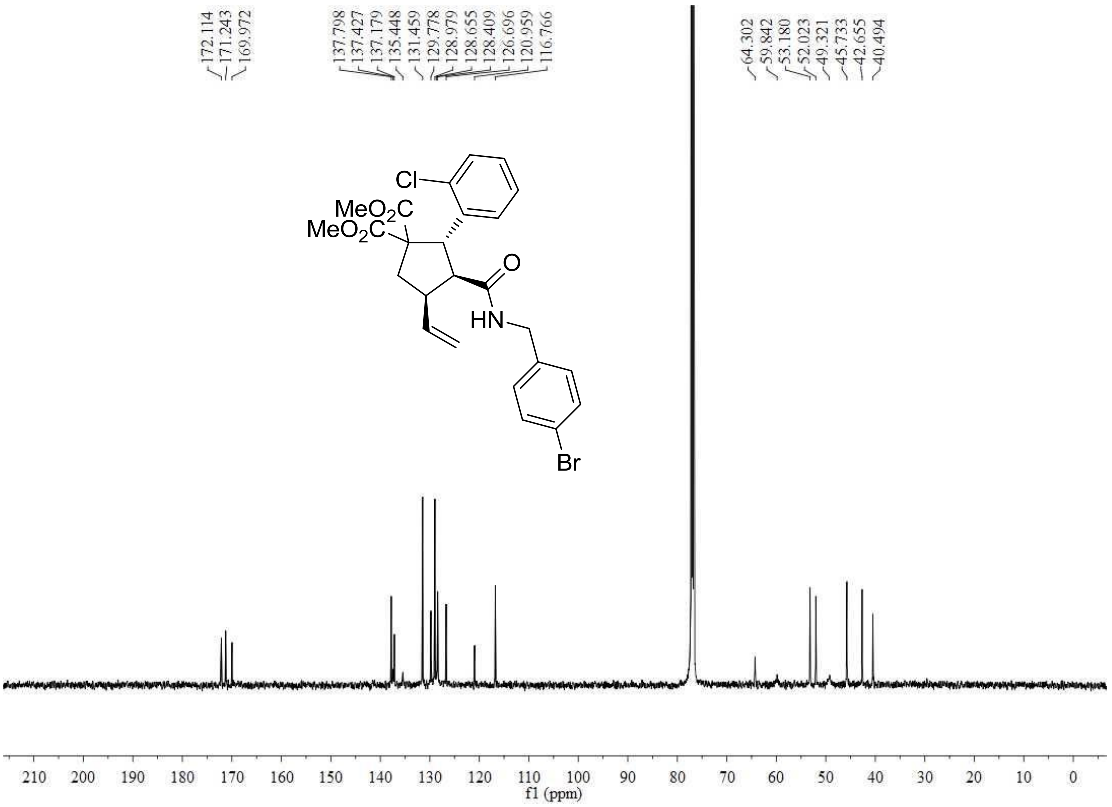

7. Chiral HPLC analysis spectra

SHIMADZU LabSolutions **Analysis Report**

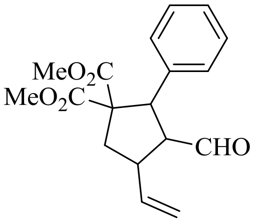

rac-**3a**

<Sample Information>

Sample Name	: ZHP-H-178-1		
Sample ID	: ZHP-H-178-1		
Data Filename	: ZHP-H-178-1.lcd		
Method Filename	: A99X-1.lcm		
Batch Filename	:		
Vial #	: 1-1	Sample Type	: Unknown
Injection Volume	: 10 uL		
Date Acquired	: 2013/5/22 16:02:10	Acquired by	: System Administrator
Date Processed	: 2013/5/22 16:54:17	Processed by	: System Administrator

<Chromatogram>

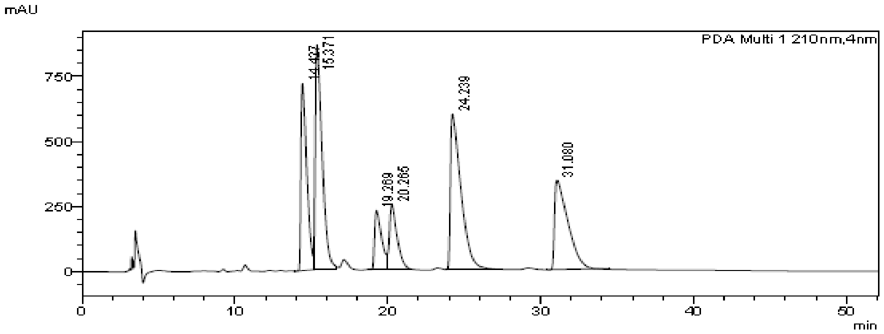

<Peak Table>

PDA Ch1 210nm

Peak#	Ret. Time	Area	Height	Conc.	Unit	Mark	Name
1	14.427	19518048	719270	17.237	%	M	RT:14.427
2	15.371	27541877	864625	24.323	%	M	RT:15.371
3	19.269	7387980	228021	6.524	%	M	RT:19.269
4	20.265	8940628	251233	7.896	%	M	RT:20.265
5	24.239	29230336	598257	25.814	%	M	RT:24.239
6	31.080	20615903	344014	18.206	%	M	RT:31.080
Total		113234772	3005420				

SHIMADZU LabSolutions **Analysis Report**

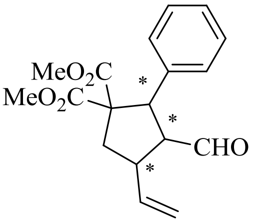

3a

<Sample Information>

Sample Name	: ZHP-H-104-1		
Sample ID	: ZHP-H-104-1		
Data Filename	: ZHP-H-104-1.lcd		
Method Filename	: A99X-1.lcm		
Batch Filename	:		
Vial #	: 1-1	Sample Type	: Unknown
Injection Volume	: 10 uL		
Date Acquired	: 2013/5/22 17:05:02	Acquired by	: System Administrator
Date Processed	: 2013/5/22 18:00:47	Processed by	: System Administrator

<Chromatogram>

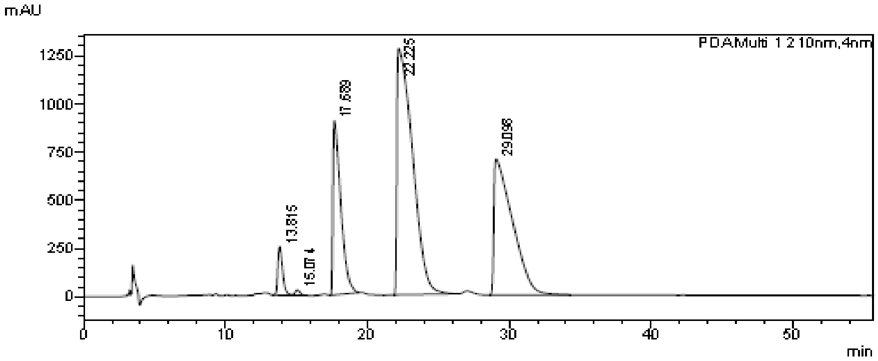

<Peak Table>

PDA Ch1 210nm

Peak#	Ret. Time	Area	Height	Conc.	Unit	Mark	Name
1	13.815	5965923	252964	2.916	%		RT:13.815
2	15.074	658983	27921	0.322	%	V	RT:15.074
3	17.689	35049369	903576	17.133	%		RT:17.689
4	22.225	98738376	1277544	48.267	%		RT:22.225
5	29.098	64154716	705762	31.361	%		RT:29.098
Total		204567366	3167767				

SHIMADZU LabSolutions

Analysis Report

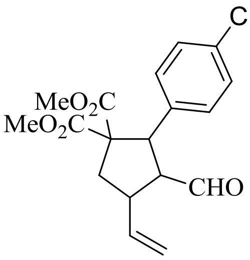

rac-**3b**

<Sample Information>

Sample Name	: zhp-h-110-8		
Sample ID	: zhp-h-110-8		
Data Filename	: zhp-h-110-8.lcd		
Method Filename	: A99X-1.lcm		
Batch Filename	:		
Vial #	: 1-1	Sample Type	: Unknown
Injection Volume	: 10 uL		
Date Acquired	: 2013/2/28 17:45:39	Acquired by	: System Administrator
Date Processed	: 2013/2/28 19:48:03	Processed by	: System Administrator

<Chromatogram>

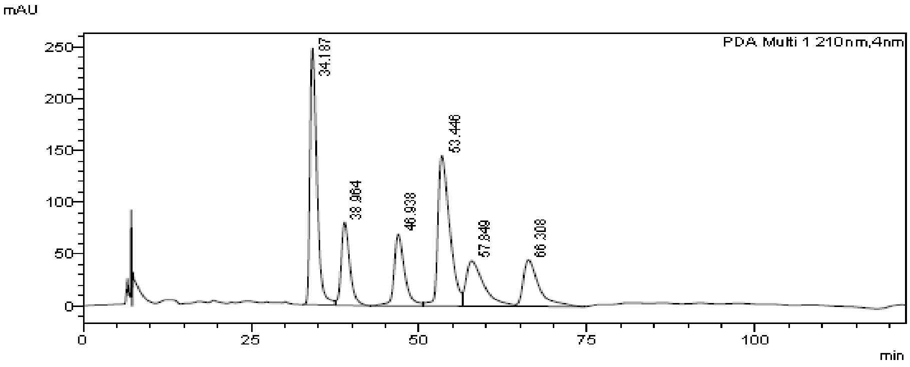

<Peak Table>

PDA Ch1 210nm

Peak#	Ret. Time	Area	Height	Conc.	Unit	Mark	Name
1	34.187	17683997	243691	26.898	%		RT:34.187
2	38.964	7087717	79184	10.781	%	V	RT:38.964
3	46.938	7576776	68476	11.525	%		RT:46.938
4	53.446	17960587	143601	27.319	%	V	RT:53.446
5	57.849	8063101	43037	12.264	%	V	RT:57.849
6	66.308	7372040	44593	11.213	%	V	RT:66.308
Total		65744218	622581				

SHIMADZU LabSolutions

Analysis Report

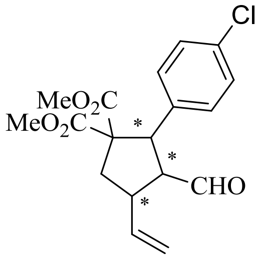

3b

<Sample Information>

Sample Name	: zhp-h-96-2		
Sample ID	: zhp-h-96-2		
Data Filename	: zhp-h-96-2.lcd		
Method Filename	: A99X-1.lcm		
Batch Filename	:		
Vial #	: 1-1	Sample Type	: Unknown
Injection Volume	: 10 uL		
Date Acquired	: 2013/2/28 21:13:46	Acquired by	: System Administrator
Date Processed	: 2013/2/28 23:09:28	Processed by	: System Administrator

<Chromatogram>

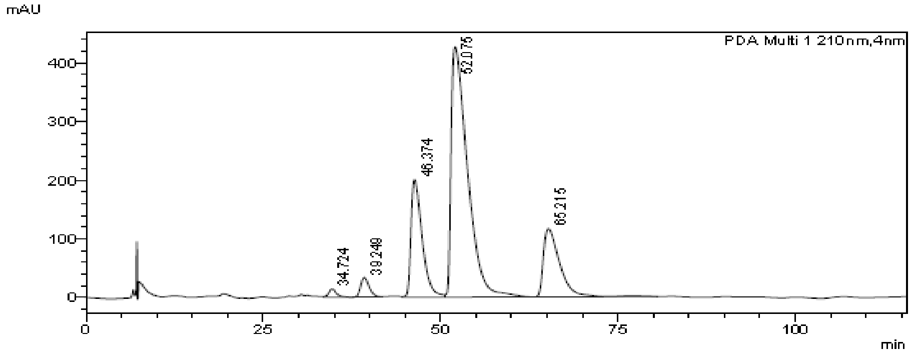

<Peak Table>

PDA Ch1 210nm

Peak#	Ret. Time	Area	Height	Conc.	Unit	Mark	Name
1	34.724	1115346	14290	0.989	%	V	RT:34.724
2	39.249	2936537	33813	2.605	%		RT:39.249
3	46.374	21934246	199963	19.457	%	V	RT:46.374
4	52.075	68295659	426668	60.583	%	V	RT:52.075
5	65.215	18449441	116408	16.366	%	S	RT:65.215
Total		112731228	791142				

SHIMADZU LabSolutions **Analysis Report**

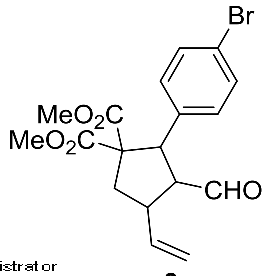

rac-**3c**

<Sample Information>

Sample Name	: zhp-h-10		
Sample ID	: zhp-h-10		
Data Filename	: zhp-h--109-10.lcd		
Method Filename	: A99X-1.lcm		
Batch Filename	:		
Vial #	: 1-1	Sample Type	: Unknown
Injection Volume	: 10 uL		
Date Acquired	: 2013/2/23 17:09:07	Acquired by	: System Administrator
Date Processed	: 2013/2/25 15:51:50	Processed by	: System Administrator

<Chromatogram>

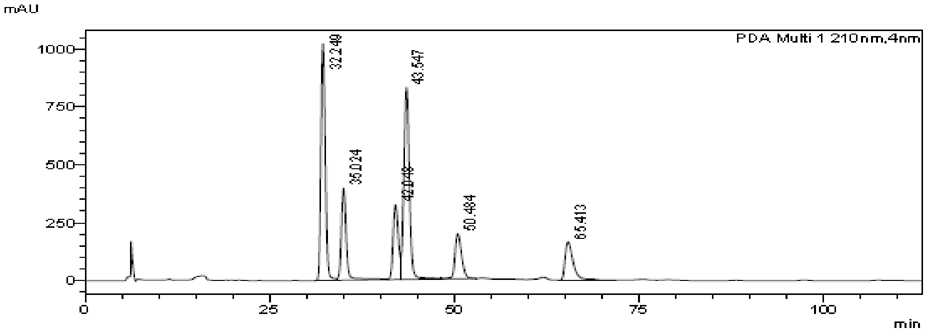

<Peak Table>

PDA Ch1 210nm

Peak#	Ret. Time	Area	Height	Conc.	Unit	Mark	Name
1	32.249	42345109	1019177	29.120	%		RT:32.249
2	35.024	18765123	395555	12.904	%	SV	RT:35.024
3	42.048	15931323	322665	10.956	%	V	RT:42.048
4	43.547	43577268	822122	29.967	%	V	RT:43.547
5	50.484	12025296	194794	8.270	%	V	RT:50.484
6	65.413	12771334	165005	8.783	%		RT:65.413
Total		145415454	2919318				

SHIMADZU LabSolutions **Analysis Report**

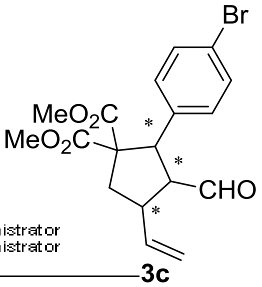

3c

<Sample Information>

Sample Name	: zhp-h-95-4		
Sample ID	: zhp-h-95-4		
Data Filename	: zhp-h-95-4.lcd		
Method Filename	: A99X-1.lcm		
Batch Filename	:		
Vial #	: 1-1	Sample Type	: Unknown
Injection Volume	: 10 uL		
Date Acquired	: 2013/2/28 9:31:45	Acquired by	: System Administrator
Date Processed	: 2013/2/28 10:53:52	Processed by	: System Administrator

<Chromatogram>

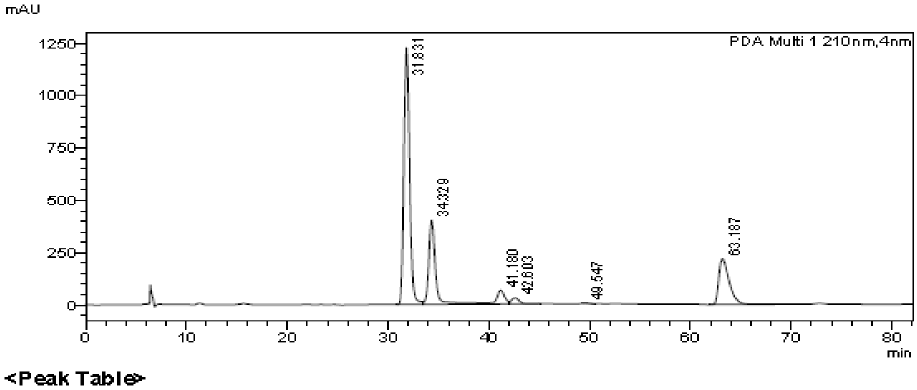

<Peak Table>

PDA Ch1 210nm

Peak#	Ret. Time	Area	Height	Conc.	Unit	Mark	Name
1	31.831	48811499	1225574	54.791	%		RT:31.831
2	34.329	19188860	401740	21.540	%	SV	RT:34.329
3	41.180	3763274	66481	4.224	%	V	RT:41.180
4	42.603	1528908	29256	1.716	%	V	RT:42.603
5	49.547	106368	3144	0.119	%	M	RT:49.547
6	63.187	15687208	218911	17.609	%	S	RT:63.187
Total		89086116	1945105				

SHIMADZU LabSolutions

Analysis Report

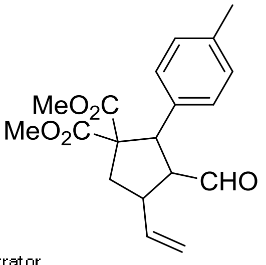

rac-**3d**

<Sample Information>

Sample Name	: zhp-h-111--3		
Sample ID	: zhp-h-111--3		
Data Filename	: zhp-h-111--3.lcd		
Method Filename	: A99X-1.lcm		
Batch Filename	:		
Vial #	: 1-1	Sample Type	: Unknown
Injection Volume	: 10 uL		
Date Acquired	: 2013/3/2 11:49:40	Acquired by	: System Administrator
Date Processed	: 2013/3/2 19:43:16	Processed by	: System Administrator

<Chromatogram>

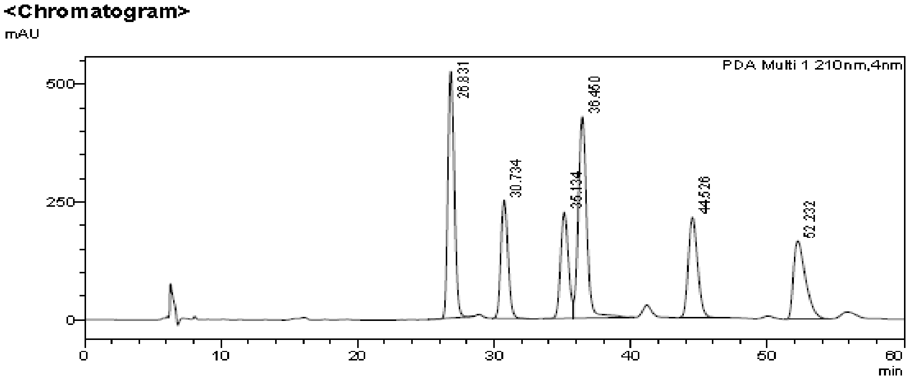

<Peak Table>

PDA Ch1 210nm

Peak#	Ret. Time	Area	Height	Conc.	Unit	Mark	Name
1	26.831	16963454	524563	23.078	%	M	RT:26.831
2	30.734	9094903	250968	12.373	%		RT:30.734
3	35.134	8983872	225572	12.222	%		RT:35.134
4	36.450	18253942	427403	24.834	%	V	RT:36.450
5	44.526	10041084	212358	13.661	%		RT:44.526
6	52.232	10167218	165472	13.832	%		RT:52.232
Total		73504473	1806336				

SHIMADZU LabSolutions

Analysis Report

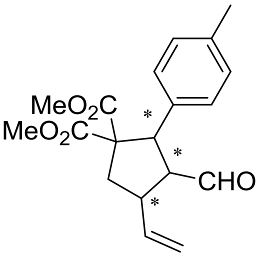

3d

<Sample Information>

Sample Name	: zhp-h-97-1		
Sample ID	: zhp-h-97-1		
Data Filename	: zhp-h-97-1.lcd		
Method Filename	: A99X-1.lcm		
Batch Filename	:		
Vial #	: 1-1	Sample Type	: Unknown
Injection Volume	: 10 uL		
Date Acquired	: 2013/3/2 10:38:38	Acquired by	: System Administrator
Date Processed	: 2013/3/2 19:43:45	Processed by	: System Administrator

<Chromatogram>

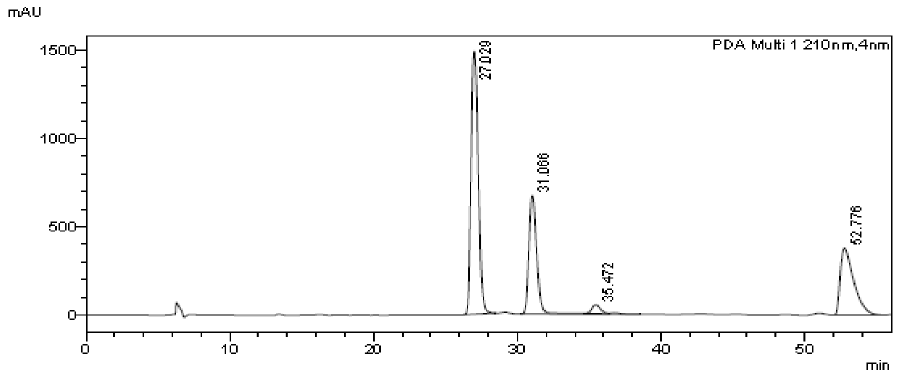

<Peak Table>

PDA Ch1 210nm

Peak#	Ret. Time	Area	Height	Conc.	Unit	Mark	Name
1	27.029	52250742	1490285	49.560	%	M	RT:27.029
2	31.066	26232617	672838	24.882	%	S	RT:31.066
3	35.472	2112324	50142	2.004	%	TV	RT:35.472
4	52.776	24832792	378869	23.554	%		RT:52.776
Total		105428475	2592134				

Analysis Report

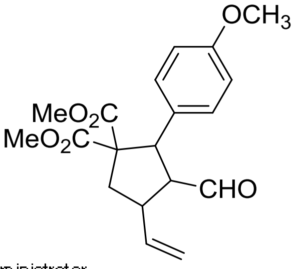

rac-**3e**

<Sample Information>

Sample Name	: zhp-h-118-27		
Sample ID	: zhp-h-118-27		
Data Filename	: zhp-h-118-27.lcd		
Method Filename	: A99X-1.lcm		
Batch Filename	:		
Vial #	: 1-1	Sample Type	: Unknown
Injection Volume	: 10 uL		
Date Acquired	: 2013/3/26 18:41:30	Acquired by	: System Administrator
Date Processed	: 2013/4/17 22:58:43	Processed by	: System Administrator

<Chromatogram>

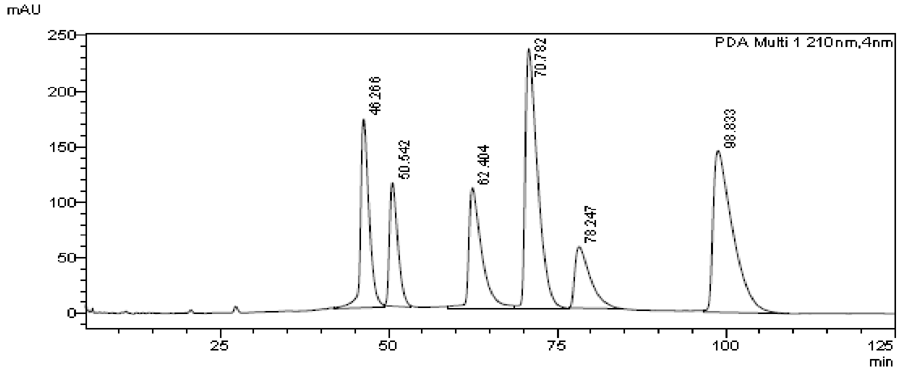

<Peak Table>

PDA Ch1 210nm

Peak#	Ret. Time	Area	Height	Conc.	Unit	Mark	Name
1	46.266	14984378	169813	13.934	%	M	RT:46.266
2	50.542	9503683	111107	8.838	%	M	RT:50.542
3	62.404	14819421	108894	13.781	%	M	RT:62.404
4	70.782	29906696	234159	27.811	%	M	RT:70.782
5	78.247	8836787	55125	8.218	%		RT:78.247
6	98.833	29483930	145053	27.418	%	M	RT:98.833
Total		107534895	824151				

Analysis Report

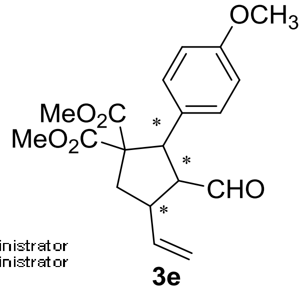

3e

<Sample Information>

Sample Name	: zhp-h-117-10		
Sample ID	: zhp-h-117-10		
Data Filename	: zhp-h-117-10.lcd		
Method Filename	: A99X-1.lcm		
Batch Filename	:		
Vial #	: 1-1	Sample Type	: Unknown
Injection Volume	: 10 uL		
Date Acquired	: 2013/4/18 19:58:47	Acquired by	: System Administrator
Date Processed	: 2013/4/18 22:03:55	Processed by	: System Administrator

<Chromatogram>

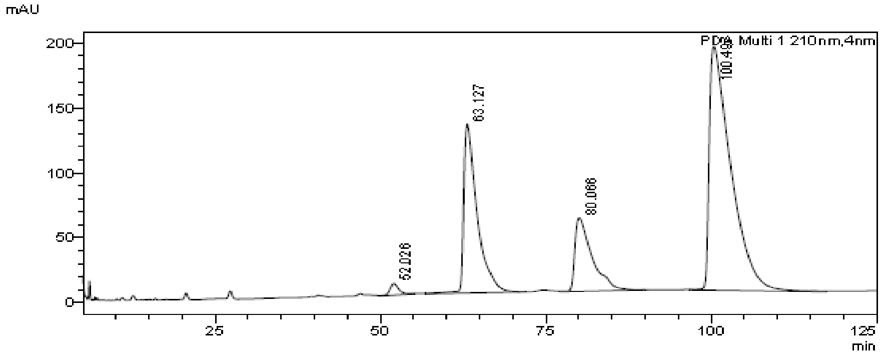

<Peak Table>

PDA Ch1 210nm

Peak#	Ret. Time	Area	Height	Conc.	Unit	Mark	Name
1	52.026	829263	8610	1.148	%	M	RT:52.026
2	63.127	18344781	129699	25.396	%	M	RT:63.127
3	80.066	10263485	56269	14.209	%	M	RT:80.066
4	100.497	42796150	186611	59.247	%	M	RT:100.497
Total		72233678	381190				

SHIMADZU LabSolutions

Analysis Report

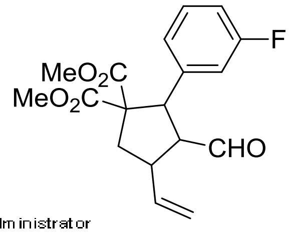

rac-3f

<Sample Information>

Sample Name	: zhp-h-116-6		
Sample ID	: zhp-h-116-6		
Data Filename	: zhp-h-116-6.lcd		
Method Filename	: A99X-1.lcm		
Batch Filename	:		
Vial #	: 1-1	Sample Type	: Unknown
Injection Volume	: 10 uL		
Date Acquired	: 2013/3/21 17:35:54	Acquired by	: System Administrator
Date Processed	: 2013/3/21 21:01:20	Processed by	: System Administrator

<Chromatogram>

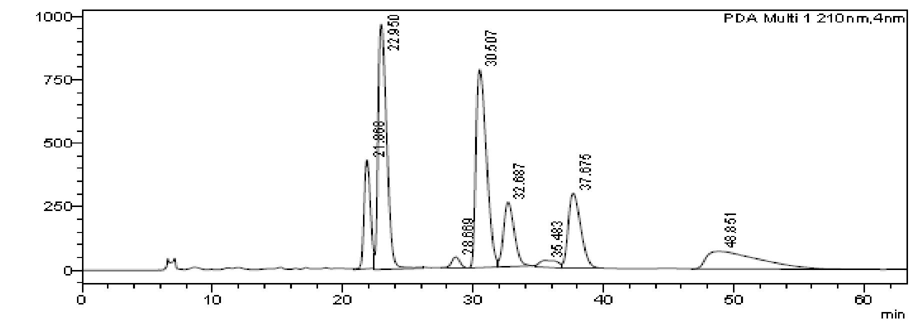

<Peak Table>

PDA Ch1 210nm

Peak#	Ret. Time	Area	Height	Conc.	Unit	Mark	Name
1	21.868	14402439	418047	8.911	%	M	RT:21.868
2	22.950	44184103	965803	27.338	%	M	RT:22.950
3	28.669	1900513	43305	1.176	%	M	RT:28.669
4	30.507	44211599	779223	27.355	%		RT:30.507
5	32.687	14617774	253075	9.044	%	V	RT:32.687
6	35.483	2766745	27746	1.712	%	M	RT:35.483
7	37.675	19668378	295041	12.169	%	M	RT:37.675
8	48.851	19869805	69487	12.294	%		RT:48.851
Total		161621357	2851727				

SHIMADZU LabSolutions

Analysis Report

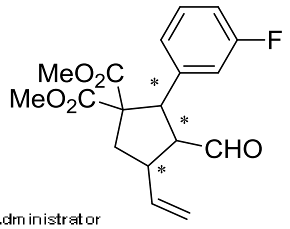

3f

<Sample Information>

Sample Name	: zhp-h-115-2		
Sample ID	: zhp-h-115-2		
Data Filename	: zhp-h-115-2.lcd		
Method Filename	: A99X-1.lcm		
Batch Filename	:		
Vial #	: 1-1	Sample Type	: Unknown
Injection Volume	: 10 uL		
Date Acquired	: 2013/3/21 19:35:36	Acquired by	: System Administrator
Date Processed	: 2013/3/22 9:48:01	Processed by	: System Administrator

<Chromatogram>

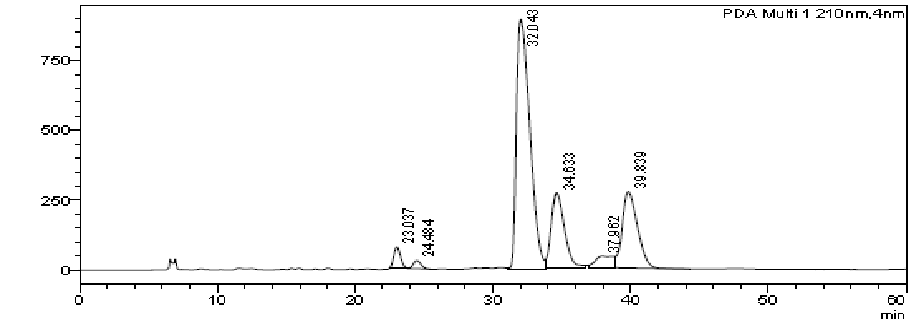

<Peak Table>

PDA Ch1 210nm

Peak#	Ret. Time	Area	Height	Conc.	Unit	Mark	Name
1	23.037	2468517	73477	2.302	%	M	RT:23.037
2	24.484	1095130	29259	1.021	%	M	RT:24.484
3	32.043	60646205	891478	56.561	%	M	RT:32.043
4	34.633	17976614	267743	16.766	%	M	RT:34.633
5	37.962	3903423	42604	3.640	%	M	RT:37.962
6	39.839	21133268	272918	19.710	%	M	RT:39.839
Total		107223157	1577479				

Analysis Report

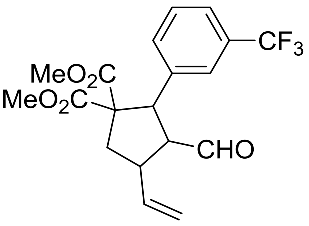

rac-**3g**

<Sample Information>

Sample Name : zhp-h-124-5
Sample ID : zhp-h-124-5
Data Filename : zhp-h-124-5.lcd
Method Filename : A99X-1.lcm
Batch Filename :
Vial # : 1-1
Injection Volume : 10 uL
Date Acquired : 2013/3/6 14:22:56
Date Processed : 2013/3/6 15:26:16

Sample Type : Unknown
Acquired by : System Administrator
Processed by : System Administrator

<Chromatogram>

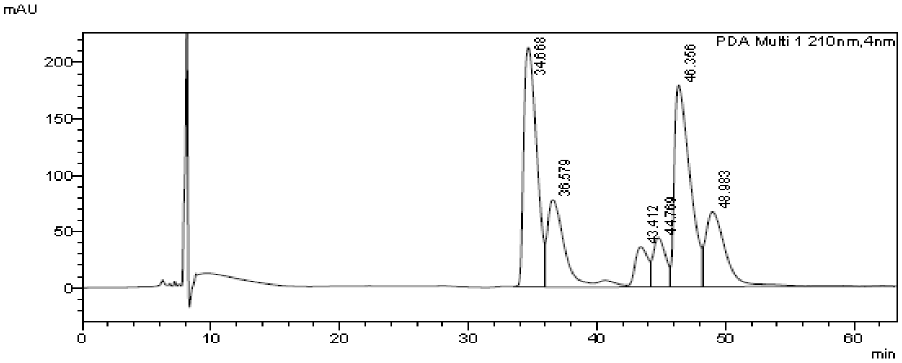

<Peak Table>

PDA Ch1 210nm

Peak#	Ret. Time	Area	Height	Conc.	Unit	Mark	Name
1	34.668	14977427	212612	29.925	%	V	RT:34.668
2	36.579	7286978	77349	14.559	%	SV	RT:36.579
3	43.412	2390503	35429	4.776	%	V	RT:43.412
4	44.769	2997012	43721	5.988	%	V	RT:44.769
5	46.356	15129068	178709	30.228	%	V	RT:46.356
6	48.983	7268880	66540	14.523	%	SV	RT:48.983
Total		50049868	614360				

Analysis Report

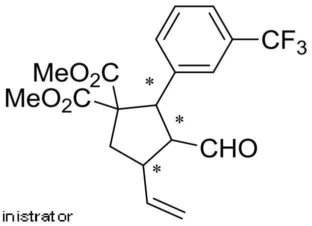

3g

<Sample Information>

Sample Name : ZHP-H-123-1
Sample ID : ZHP-H-123-1
Data Filename : ZHP-H-123-1.lcd
Method Filename : A99X-1.lcm
Batch Filename :
Vial # : 1-1
Injection Volume : 10 uL
Date Acquired : 2013/3/9 9:10:49
Date Processed : 2013/3/9 10:21:39

Sample Type : Unknown
Acquired by : System Administrator
Processed by : System Administrator

<Chromatogram>

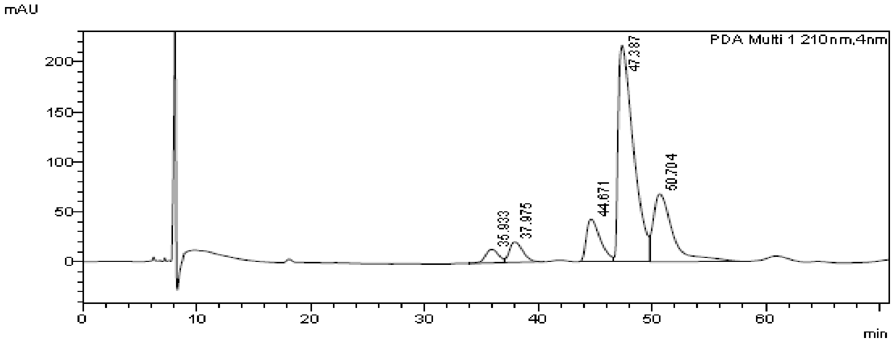

<Peak Table>

PDA Ch1 210nm

Peak#	Ret. Time	Area	Height	Conc.	Unit	Mark	Name
1	35.933	1080987	13437	3.126	%	V	RT:35.933
2	37.975	1665023	20273	4.815	%	V	RT:37.975
3	44.671	3615528	42224	10.455	%		RT:44.671
4	47.387	20136406	216302	58.228	%	V	RT:47.387
5	50.704	8084270	67588	23.377	%	V	RT:50.704
Total		34582215	359823				

SHIMADZU LabSolutions Analysis Report

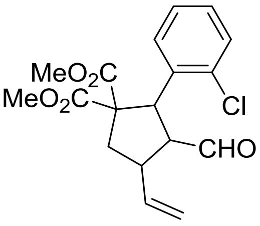

rac-**3h'**

<Sample Information>

Sample Name : zhp-h-113-3-3
Sample ID : zhp-h-113-3-3
Data Filename : zhp-h-113-3-3.lcd
Method Filename : A99X-1.lcm
Batch Filename :
Vial # : 1-1
Injection Volume : 10 uL
Date Acquired : 2013/3/20 19:40:34
Date Processed : 2013/3/20 20:30:37
Sample Type : Unknown
Acquired by : System Administrator
Processed by : System Administrator

<Chromatogram>

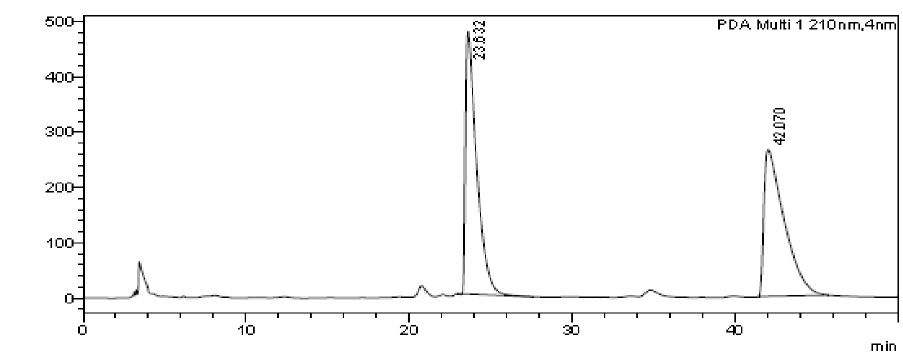

<Peak Table>

PDA Ch1 210nm

Peak#	Ret. Time	Area	Height	Conc.	Unit	Mark	Name
1	23.632	22763276	474214	49.711	%	M	RT:23.632
2	42.070	23027803	264010	50.289	%	M	RT:42.070
Total		45791080	738224				

SHIMADZU LabSolutions Analysis Report

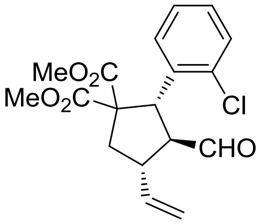

3h'

<Sample Information>

Sample Name : zhp-h-114-3-2
Sample ID : zhp-h-114-3-2
Data Filename : zhp-h-114-3-2.lcd
Method Filename : A99X-1.lcm
Batch Filename :
Vial # : 1-1
Injection Volume : 10 uL
Date Acquired : 2013/3/20 17:53:46
Date Processed : 2013/3/20 18:43:49
Sample Type : Unknown
Acquired by : System Administrator
Processed by : System Administrator

<Chromatogram>

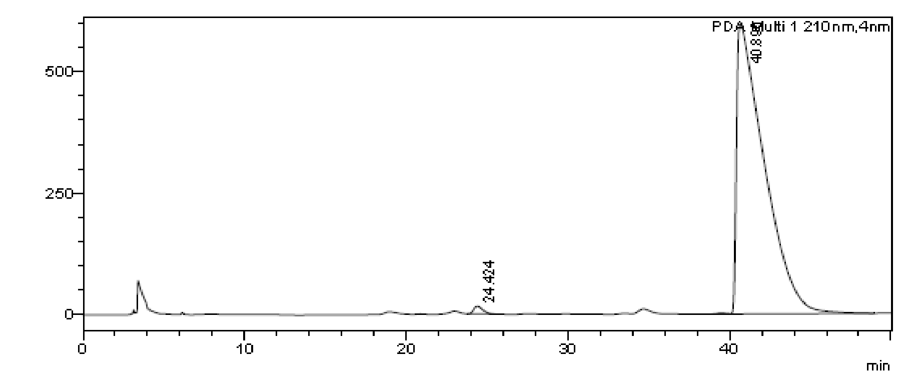

<Peak Table>

PDA Ch1 210nm

Peak#	Ret. Time	Area	Height	Conc.	Unit	Mark	Name
1	24.424	600767	13175	0.836	%	M	RT:24.424
2	40.896	71283019	579041	99.164	%		RT:40.896
Total		71883787	592216				

SHIMADZU LabSolutions **Analysis Report**

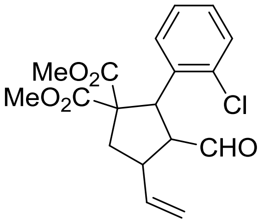

rac-**3h''**

<Sample Information>

Sample Name	: zhp-h-113-2-2		
Sample ID	: zhp-h-113-2-2		
Data Filename	: zhp-h-113-2-2.lcd		
Method Filename	: A99X-1.lcm		
Batch Filename	:		
Vial #	: 1-1	Sample Type	: Unknown
Injection Volume	: 10 uL		
Date Acquired	: 2013/3/20 16:51:57	Acquired by	: System Administrator
Date Processed	: 2013/3/20 17:42:01	Processed by	: System Administrator

<Chromatogram>

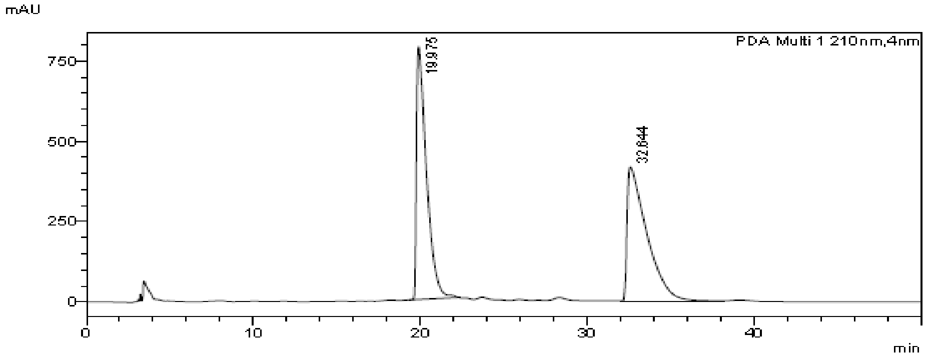

<Peak Table>

PDA Ch1 210nm

Peak#	Ret. Time	Area	Height	Conc.	Unit	Mark	Name
1	19.975	32967737	769865	49.174	%	M	RT:19.975
2	32.644	34075723	406174	50.826	%		RT:32.644
Total		67043460	1176038				

SHIMADZU LabSolutions **Analysis Report**

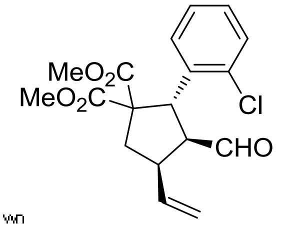

3h''

<Sample Information>

Sample Name	: zhp-h-114-2-3		
Sample ID	: zhp-h-114-2-3		
Data Filename	: zhp-h-114-2-3.lcd		
Method Filename	: A99X-1.lcm		
Batch Filename	:		
Vial #	: 1-1	Sample Type	: Unknown
Injection Volume	: 10 uL		
Date Acquired	: 2013/3/20 15:44:16	Acquired by	: System Administrator
Date Processed	: 2013/3/20 16:32:37	Processed by	: System Administrator

<Chromatogram>

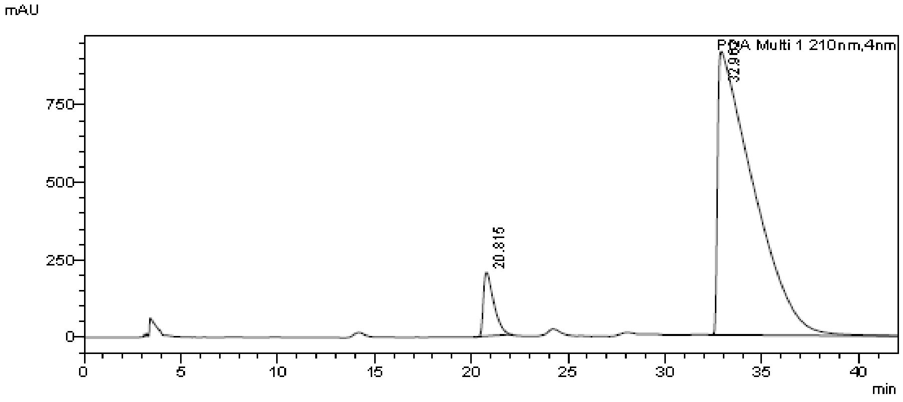

<Peak Table>

PDA Ch1 210nm

Peak#	Ret. Time	Area	Height	Conc.	Unit	Mark	Name
1	20.815	7641343	189752	6.099	%	M	RT:20.815
2	32.962	117653935	904946	93.901	%		RT:32.962
Total		125295278	1094698				

SHIMADZU LabSolutions

Analysis Report

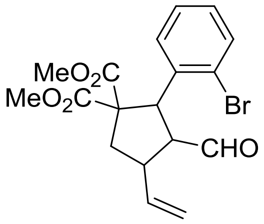

rac-**3i'**

<Sample Information>

Sample Name	: ZHP-H-147-3-1		
Sample ID	: ZHP-H-147-3-1		
Data Filename	: ZHP-H-147-3-1.lcd		
Method Filename	: A99X-1.lcm		
Batch Filename	:		
Vial #	: 1-1	Sample Type	: Unknown
Injection Volume	: 10 uL		
Date Acquired	: 2013/3/23 15:36:03	Acquired by	: System Administrator
Date Processed	: 2013/3/23 16:36:12	Processed by	: System Administrator

<Chromatogram>

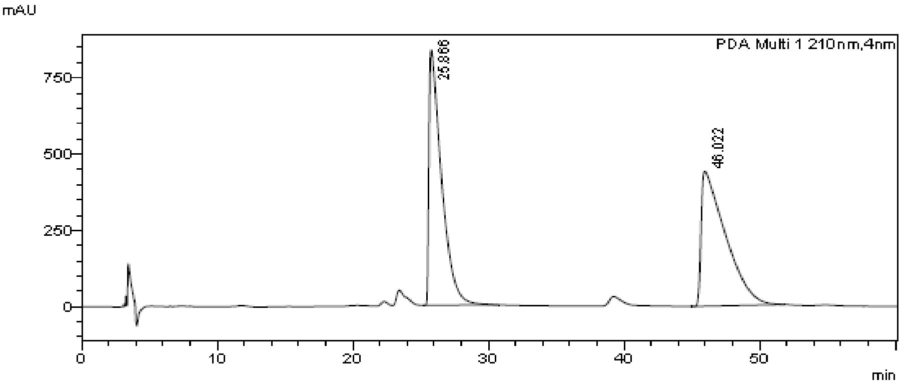

<Peak Table>

PDA Ch1 210nm

Peak#	Ret. Time	Area	Height	Conc.	Unit	Mark	Name
1	25.866	53623301	824644	49.323	%		RT:25.866
2	46.022	55094432	438882	50.677	%		RT:46.022
Total		108717734	1263526				

SHIMADZU LabSolutions

Analysis Report

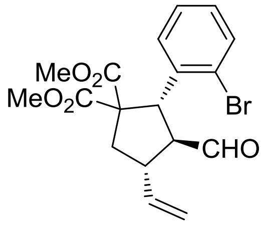

3i'

<Sample Information>

Sample Name	: ZHP-H-144-3-2		
Sample ID	: ZHP-H-144-3-2		
Data Filename	: ZHP-H-144-3-2.lcd		
Method Filename	: A99X-1.lcm		
Batch Filename	:		
Vial #	: 1-1	Sample Type	: Unknown
Injection Volume	: 10 uL		
Date Acquired	: 2013/3/23 16:46:05	Acquired by	: System Administrator
Date Processed	: 2013/3/23 17:52:39	Processed by	: System Administrator

<Chromatogram>

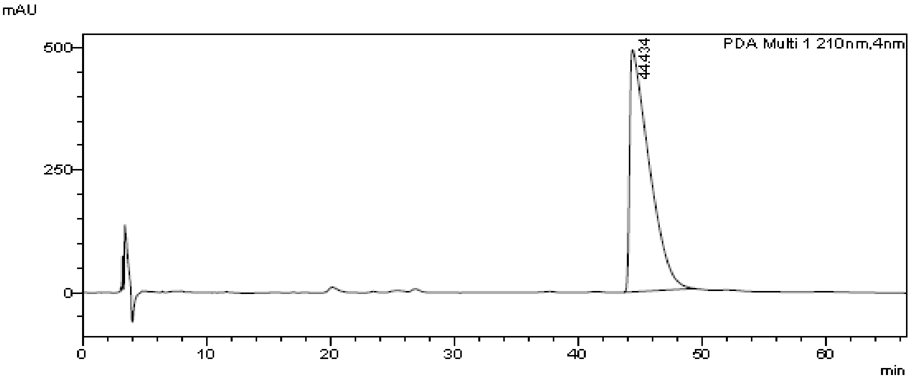

<Peak Table>

PDA Ch1 210nm

Peak#	Ret. Time	Area	Height	Conc.	Unit	Mark	Name
1	44.434	54269241	494242	100.000	%		RT:44.434
Total		54269241	494242				

SHIMADZU LabSolutions

Analysis Report

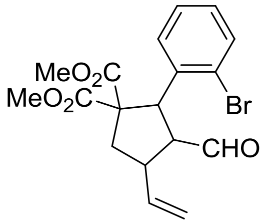

rac-**3i''**

<Sample Information>

Sample Name	: ZHP-H-147-3-1		
Sample ID	: ZHP-H-147-3-1		
Data Filename	: ZHP-H-147-3-2.lcd		
Method Filename	: A99X-1.lcm		
Batch Filename	:		
Vial #	: 1-1	Sample Type	: Unknown
Injection Volume	: 10 uL		
Date Acquired	: 2013/3/23 18:04:41	Acquired by	: System Administrator
Date Processed	: 2013/3/23 19:34:44	Processed by	: System Administrator

<Chromatogram>

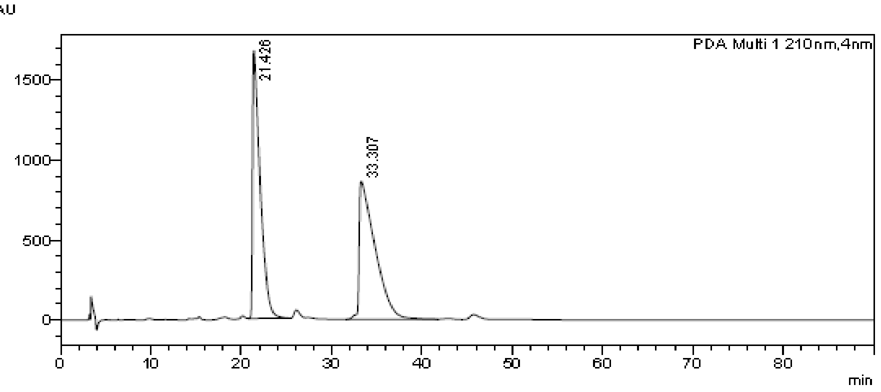

<Peak Table>

PDA Ch1 210nm

Peak#	Ret. Time	Area	Height	Conc.	Unit	Mark	Name
1	21.426	98064630	1674785	48.045	%		RT:21.426
2	33.307	106045232	862922	51.955	%		RT:33.307
Total		204109862	2537707				

SHIMADZU LabSolutions

Analysis Report

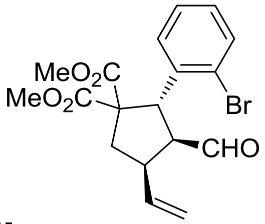

3i''

<Sample Information>

Sample Name	: ZHP-H-144-2-1		
Sample ID	: ZHP-H-144-2-1		
Data Filename	: ZHP-H-144-2-1.lcd		
Method Filename	: A99X-1.lcm		
Batch Filename	:		
Vial #	: 1-1	Sample Type	: Unknown
Injection Volume	: 10 uL		
Date Acquired	: 2013/3/23 19:47:19	Acquired by	: System Administrator
Date Processed	: 2013/3/23 20:57:23	Processed by	: System Administrator

<Chromatogram>

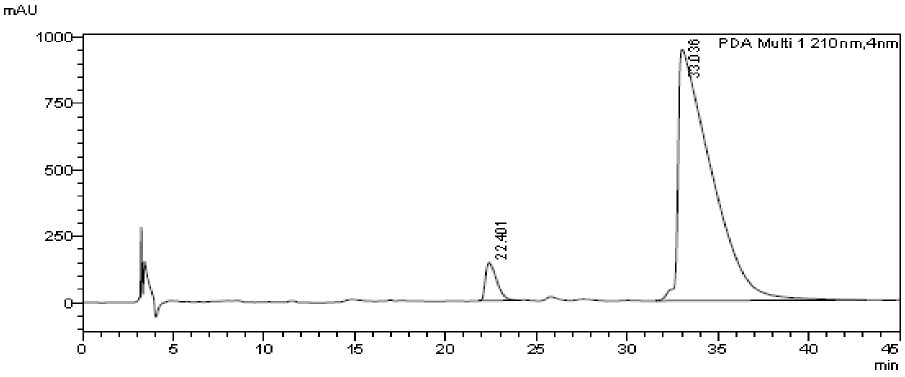

<Peak Table>

PDA Ch1 210nm

Peak#	Ret. Time	Area	Height	Conc.	Unit	Mark	Name
1	22.401	6209179	143636	4.893	%		RT:22.401
2	33.036	120679283	946415	95.107	%		RT:33.036
Total		126888463	1090051				

Analysis Report

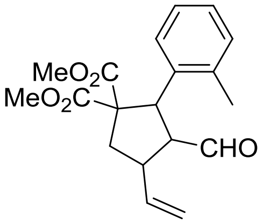

rac-**3j**

<Sample Information>

Sample Name : zhp-h-122-6
Sample ID : zhp-h-122-6
Data Filename : zhp-h-122-6.lcd
Method Filename : A99X-1.lcm
Batch Filename :
Vial # : 1-1
Injection Volume : 10 uL
Date Acquired : 2013/4/24 16:59:00
Date Processed : 2013/4/24 17:41:30

Sample Type : Unknown
Acquired by : System Administrator
Processed by : System Administrator

<Chromatogram>

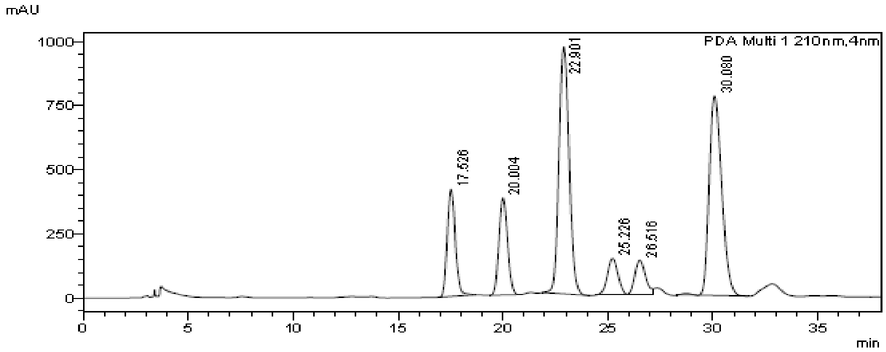

<Peak Table>

PDA Ch1 210nm

Peak#	Ret. Time	Area	Height	Conc.	Unit	Mark	Name
1	17.526	11188369	416889	11.615	%		RT:17.526
2	20.004	10740829	377341	11.151	%		RT:20.004
3	22.901	32467426	961648	33.707	%		RT:22.901
4	25.226	4877221	139482	5.063	%	M	RT:25.226
5	26.516	4687579	132768	4.867	%	M	RT:26.516
6	30.080	32361525	773342	33.597	%	M	RT:30.080
Total		96322950	2801470				

Analysis Report

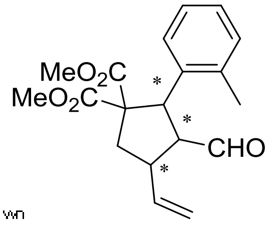

3j

<Sample Information>

Sample Name : zhp-h-121-3
Sample ID : zhp-h-121-3
Data Filename : zhp-h-121-4.lcd
Method Filename : A99X-1.lcm
Batch Filename :
Vial # : 1-1
Injection Volume : 10 uL
Date Acquired : 2013/4/24 17:48:29
Date Processed : 2013/4/24 18:55:05

Sample Type : Unknown
Acquired by : System Administrator
Processed by : System Administrator

<Chromatogram>

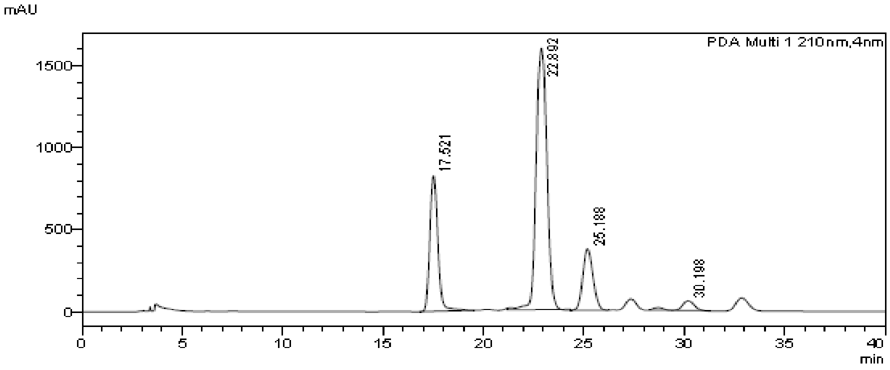

<Peak Table>

PDA Ch1 210nm

Peak#	Ret. Time	Area	Height	Conc.	Unit	Mark	Name
1	17.521	23297487	826433	23.638	%		RT:17.521
2	22.892	59209813	1592069	60.075	%	M	RT:22.892
3	25.188	13215741	373183	13.409	%		RT:25.188
4	30.198	2836885	58808	2.878	%	M	RT:30.198
Total		98559925	2850492				

SHIMADZU LabSolutions

Analysis Report

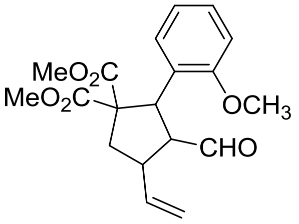

rac-**3k**

<Sample Information>

Sample Name : zhp-h-160-9
Sample ID : zhp-h-160-9
Data Filename : zhp-h-160-9.lcd
Method Filename : A99X-1.lcm
Batch Filename :
Vial # : 1-1
Injection Volume : 10 uL
Date Acquired : 2013/4/23 16:05:59
Date Processed : 2013/4/23 17:15:19

Sample Type : Unknown
Acquired by : System Administrator
Processed by : System Administrator

<Chromatogram>

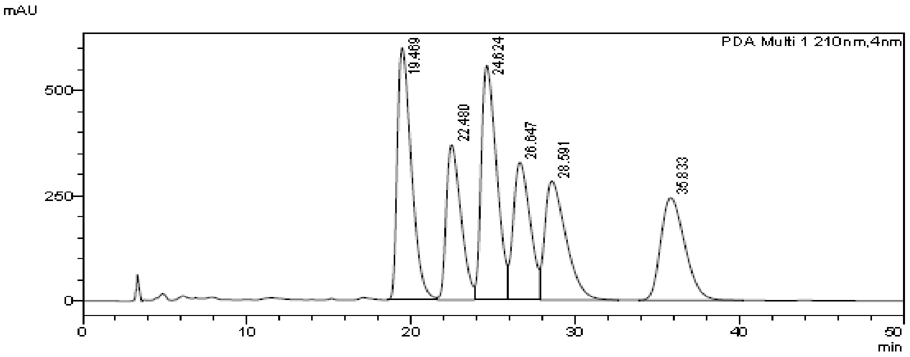

<Peak Table>

PDA Ch1 210nm

Peak#	Ret. Time	Area	Height	Conc.	Unit	Mark	Name
1	19.469	35806514	599082	21.239	%		RT:19.469
2	22.480	22939739	368073	13.607	%	V	RT:22.480
3	24.624	36369693	556599	21.573	%	V	RT:24.624
4	26.647	23402610	326353	13.881	%	V	RT:26.647
5	28.591	25288412	282215	15.000	%	V	RT:28.591
6	35.833	24782414	243064	14.700	%		RT:35.833
Total		168589383	2375386				

SHIMADZU LabSolutions

Analysis Report

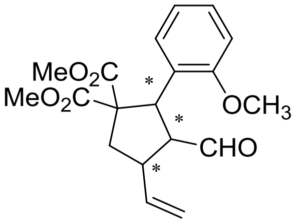

3k

<Sample Information>

Sample Name : zhp-h-161-2
Sample ID : zhp-h-161-2
Data Filename : zhp-h-161-2.lcd
Method Filename : A99X-1.lcm
Batch Filename :
Vial # : 1-1
Injection Volume : 10 uL
Date Acquired : 2013/4/23 17:27:06
Date Processed : 2013/4/23 19:40:18

Sample Type : Unknown
Acquired by : System Administrator
Processed by : System Administrator

<Chromatogram>

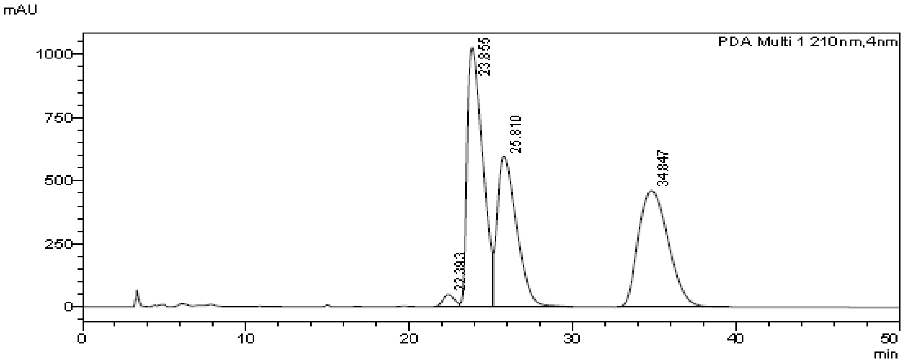

<Peak Table>

PDA Ch1 210nm

Peak#	Ret. Time	Area	Height	Conc.	Unit	Mark	Name
1	22.393	2309372	47403	1.294	%		RT:22.393
2	23.855	68326388	1024419	38.293	%	V	RT:23.855
3	25.810	48994564	594535	27.459	%	V	RT:25.810
4	34.847	58799957	460387	32.954	%		RT:34.847
Total		178430281	2126744				

SHIMADZU LabSolutions **Analysis Report**

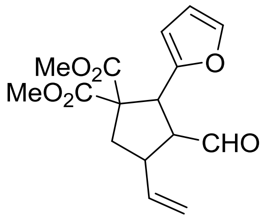

rac-**3l**

<Sample Information>

Sample Name	: zhp-h-zhen-131-1
Sample ID	: zhp-h-zhen-131-1
Data Filename	: zhp-h-zhen-131-1.lcd
Method Filename	: A99X-1.lcm
Batch Filename	:
Vial #	: 1-1
Injection Volume	: 10 uL
Date Acquired	: 2013/3/16 19:28:27
Date Processed	: 2013/3/17 19:55:33
Sample Type	: Unknown
Acquired by	: System Administrator
Processed by	: System Administrator

<Chromatogram>

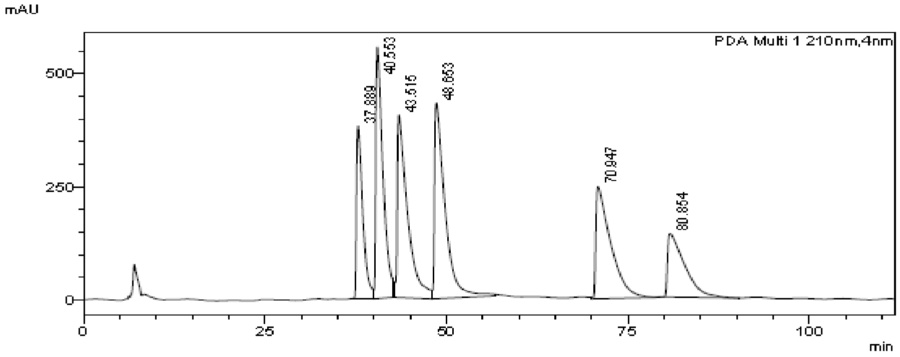

<Peak Table>

PDA Ch1 210nm

Peak#	Ret. Time	Area	Height	Conc.	Unit	Mark	Name
1	37.889	23036497	378380	11.377	%	M	RT:37.889
2	40.553	37744013	547949	18.641	%	M	RT:40.553
3	43.515	39630664	398985	19.572	%	M	RT:43.515
4	48.653	43461735	424787	21.464	%	M	RT:48.653
5	70.947	35513046	246749	17.539	%	M	RT:70.947
6	80.854	23097309	140100	11.407	%		RT:80.854
Total		202483263	2136951				

SHIMADZU LabSolutions **Analysis Report**

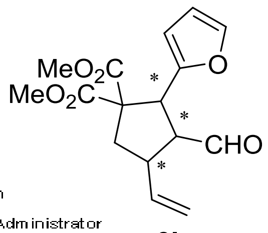

3l

<Sample Information>

Sample Name	: zhp-h-zhen-129-1
Sample ID	: zhp-h-zhen-129-1
Data Filename	: zhp-h-zhen-129-1.lcd
Method Filename	: A99X-1.lcm
Batch Filename	:
Vial #	: 1-1
Injection Volume	: 10 uL
Date Acquired	: 2013/3/17 18:04:01
Date Processed	: 2013/3/17 19:47:47
Sample Type	: Unknown
Acquired by	: System Administrator
Processed by	: System Administrator

<Chromatogram>

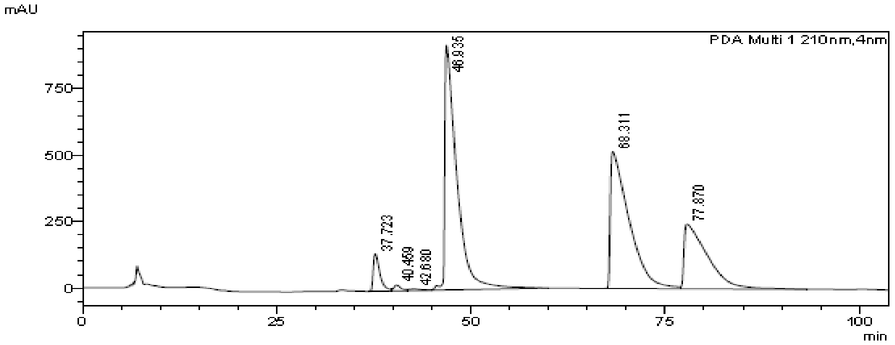

<Peak Table>

PDA Ch1 210nm

Peak#	Ret. Time	Area	Height	Conc.	Unit	Mark	Name
1	37.723	8341012	139529	3.391	%		RT:37.723
2	40.459	1342370	20895	0.546	%	V	RT:40.459
3	42.680	678659	6212	0.276	%	V	RT:42.680
4	46.935	98845418	908561	40.181	%		RT:46.935
5	68.311	85848355	513781	34.898	%		RT:68.311
6	77.870	50944143	242847	20.709	%	V	RT:77.870
Total		245999956	1831826				

SHIMADZU LabSolutions

Analysis Report

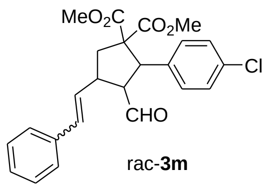

rac-**3m**

<Sample Information>

Sample Name	: ZHP-H-190-2
Sample ID	: ZHP-H-190-2
Data Filename	: ZHP-H-190-2.lcd
Method Filename	: A99X-1.lcm
Batch Filename	:
Vial #	: 1-1
Injection Volume	: 10 uL
Date Acquired	: 2013/5/21 15:37:04
Date Processed	: 2013/5/21 17:22:42

Sample Type : Unknown
Acquired by : System Administrator
Processed by : System Administrator

<Chromatogram>

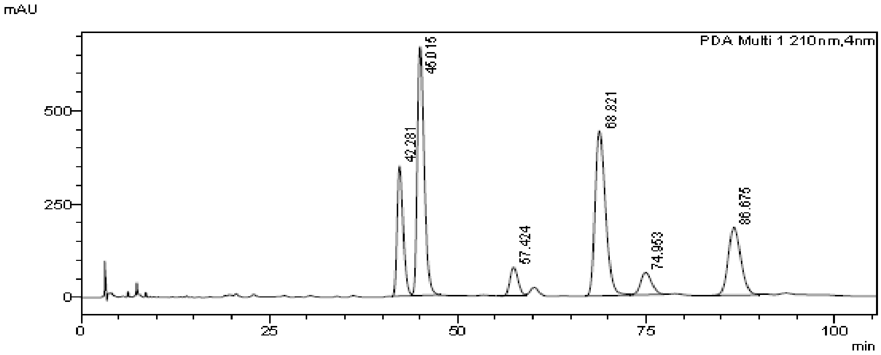

<Peak Table>

PDA Ch1 210nm

Peak#	Ret. Time	Area	Height	Conc.	Unit	Mark	Name
1	42.281	19788399	348070	14.386	%		RT:42.281
2	45.015	42176764	665565	30.661	%	V	RT:45.015
3	57.424	5526970	75759	4.018	%	M	RT:57.424
4	68.821	42578347	440748	30.953	%		RT:68.821
5	74.953	6223968	59749	4.525	%	V	RT:74.953
6	86.675	21262401	181274	15.457	%		RT:86.675
Total		137556849	1771165				

SHIMADZU LabSolutions

Analysis Report

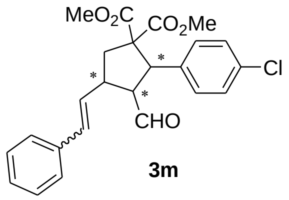

3m

<Sample Information>

Sample Name	: ZHP-H-180-1
Sample ID	: ZHP-H-180-1
Data Filename	: ZHP-H-180-1.lcd
Method Filename	: A99X-1.lcm
Batch Filename	:
Vial #	: 1-1
Injection Volume	: 10 uL
Date Acquired	: 2013/5/21 17:37:29
Date Processed	: 2013/5/21 19:42:00

Sample Type : Unknown
Acquired by : System Administrator
Processed by : System Administrator

<Chromatogram>

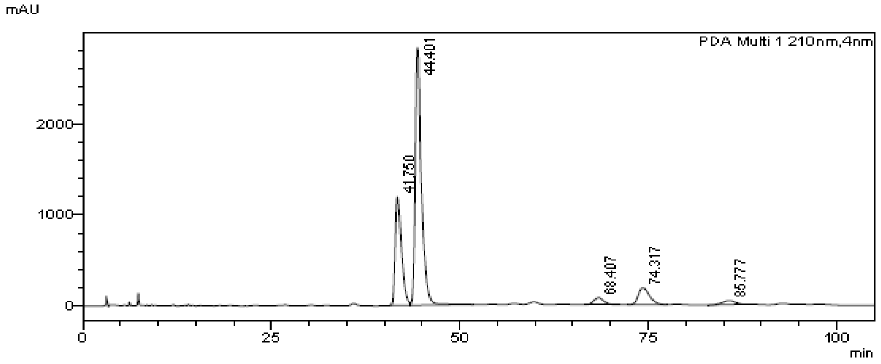

<Peak Table>

PDA Ch1 210nm

Peak#	Ret. Time	Area	Height	Conc.	Unit	Mark	Name
1	41.750	69576991	1189440	26.679	%		RT:41.750
2	44.401	159835222	2825521	61.288	%	SV	RT:44.401
3	68.407	7099309	76291	2.722	%		RT:68.407
4	74.317	19198580	184760	7.362	%		RT:74.317
5	85.777	5084026	42676	1.949	%		RT:85.777
Total		260794129	4318688				

Analysis Report

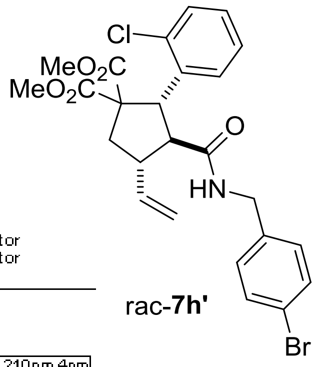

rac-**7h'**

<Sample Information>

Sample Name	: zhp-p-85-2		
Sample ID	: zhp-p-85-2		
Data Filename	: zhp-p-85-2.lcd		
Method Filename	: N.lcm		
Batch Filename	:		
Vial #	: 1-1	Sample Type	: Unknown
Injection Volume	: 10 uL		
Date Acquired	: 2013/11/26 15:12:12	Acquired by	: System Administrator
Date Processed	: 2013/11/26 17:12:15	Processed by	: System Administrator

<Chromatogram>

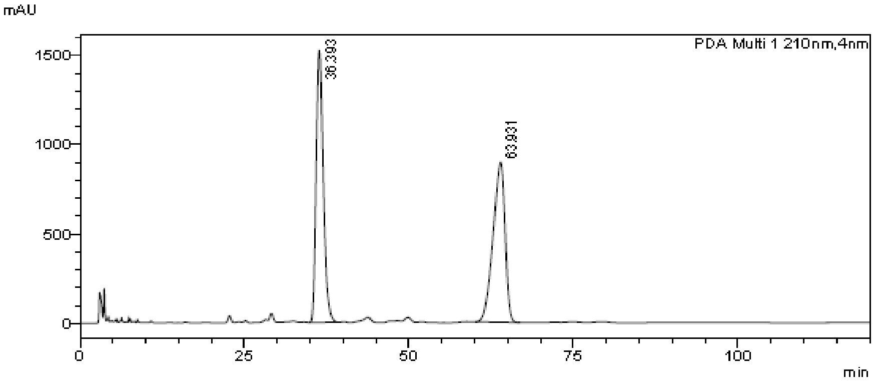

<Peak Table>

PDA Ch1 210nm

Peak#	Ret. Time	Area	Height	Conc.	Unit	Mark	Name
1	36.393	115413062	1523532	50.200	%		RT:36.393
2	63.931	114494730	895394	49.800	%		RT:63.931
Total		229907792	2418926				

Analysis Report

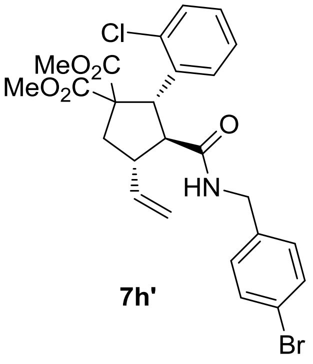

7h'

<Sample Information>

Sample Name	: zhp-p-78		
Sample ID	: zhp--p-78		
Data Filename	: zhp-p-78-AD-10%.lcd		
Method Filename	: N.lcm		
Batch Filename	:		
Vial #	: 1-1	Sample Type	: Unknown
Injection Volume	: 10 uL		
Date Acquired	: 2013/11/30 11:04:12	Acquired by	: System Administrator
Date Processed	: 2013/11/30 13:04:15	Processed by	: System Administrator

<Chromatogram>

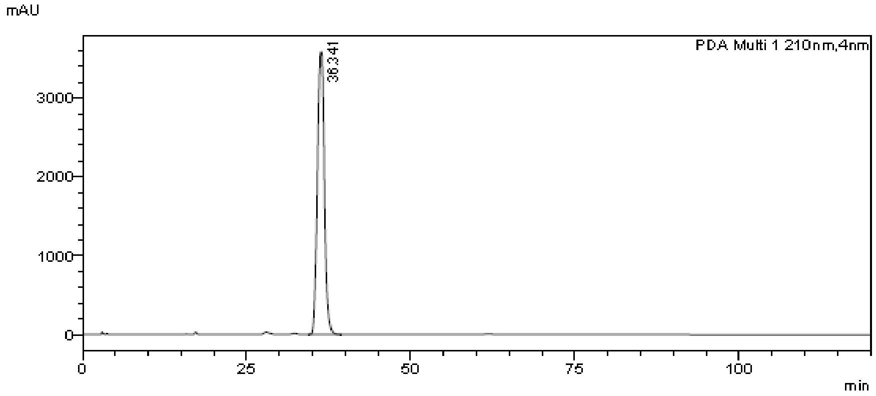

<Peak Table>

PDA Ch1 210nm

Peak#	Ret. Time	Area	Height	Conc.	Unit	Mark	Name
1	36.341	263759018	3583075	100.000	%		RT:36.341
Total		263759018	3583075				

Analysis Report

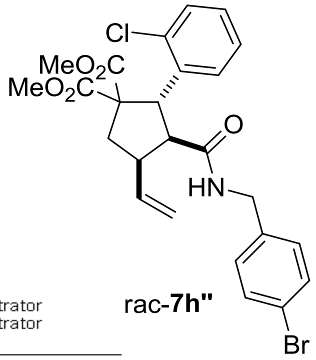

rac-7h''

<Sample Information>

Sample Name	: zhp-p-84-3		
Sample ID	: zhp-p-84-3		
Data Filename	: zhp-p-84-3.lcd		
Method Filename	: N.lcm		
Batch Filename	:		
Vial #	: 1-1	Sample Type	: Unknown
Injection Volume	: 10 uL		
Date Acquired	: 2013/12/5 17:29:52	Acquired by	: System Administrator
Date Processed	: 2013/12/5 22:03:41	Processed by	: System Administrator

<Chromatogram>

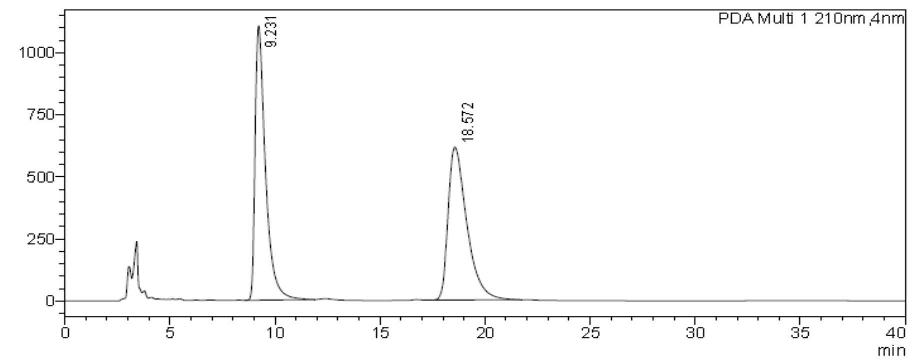

<Peak Table>

PDA Ch1 210nm

Peak#	Ret. Time	Area	Height	Conc.	Unit	Mark	Name
1	9.231	38125027	1099813	49.930	%		RT:9.231
2	18.572	38231573	615629	50.070	%		RT:18.572
Total		76356600	1715442				

Analysis Report

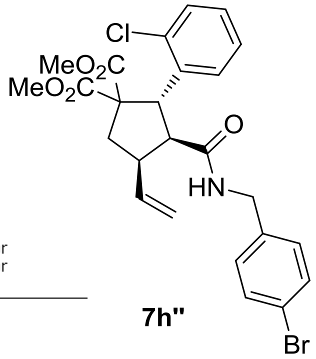

7h''

<Sample Information>

Sample Name	: zhp-p-77		
Sample ID	: zhp-p-77		
Data Filename	: zhp-p-77.lcd		
Method Filename	: N.lcm		
Batch Filename	:		
Vial #	: 1-1	Sample Type	: Unknown
Injection Volume	: 10 uL		
Date Acquired	: 2013/12/5 19:31:58	Acquired by	: System Administrator
Date Processed	: 2013/12/13 21:03:50	Processed by	: System Administrator

<Chromatogram>

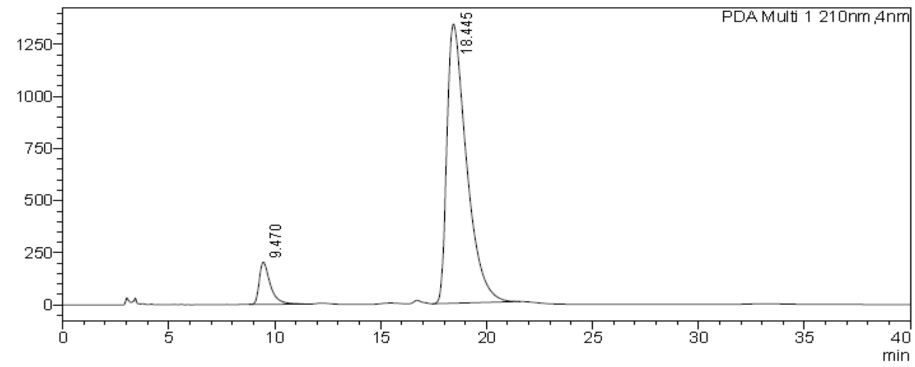

<Peak Table>

PDA Ch1 210nm

Peak#	Ret. Time	Area	Height	Conc.	Unit	Mark	Name
1	9.470	7364529	200639	7.863	%		RT:9.470
2	18.445	86298057	1332060	92.137	%		RT:18.445
Total		93662586	1532700				
